# Impact of contraceptive initiation on the cervicovaginal mucosa: results from the UMPALA multicentre, partially randomized patient-preference interventional trial

**DOI:** 10.3389/frph.2026.1882772

**Published:** 2026-10-02

**Authors:** Betty Njoroge, Nelly Mugo, Carolina Herrera, Karen Dominguez, Louise A. Ouattara, Nazita Yousefieh, Neelima Chandra, Agnieszka Kurczewska, Stephanie Nartey, Julie Lewis, Andrea R. Thurman, Matilda Saina, Raina N. Fichorova, Jacques Ravel, Reem Sharaf Alddin, Nicola Richardson-Harman, Kenneth Ngure, Gustavo F. Doncel

**Affiliations:** 1 Center for Clinical Research, Kenya Medical Research Institute, Nairobi, Kenya; 2 Department of Global Health, University of Washington, Seattle, WA, United States; 3 CONRAD, Eastern Virginia Medical School, Macon and Joan Brock Virginia Health Sciences at Old Dominion University, Norfolk, VA, United States; 4 Laboratory of Genital Tract Biology, Department of Obstetrics, Gynecology and Reproductive Biology, Brigham and Women’s Hospital and Harvard Medical School, Boston, MA, United States; 5 Center for Advanced Microbiome Research and Innovation, Institute for Genome Sciences and Department of Microbiology and Immunology, University of Maryland School of Medicine, Baltimore, MD, United States; 6 AlphaStat L.L.C., Damascus, MD, United States; 7 School of Public Health, Jomo Kenyatta University of Agriculture and Technology, Nairobi, Kenya

**Keywords:** cervicovaginal, copper intrauterine device, depot medroxyprogesterone acetate, etonogestrel, HIV, HSV-2, levonorgestrel, oestrogen

## Abstract

**Background:**

Long-acting contraceptives are widely used, providing superior efficacy and systemic safety; however, the impact of contraceptive initiation on female genital tract function and health has been understudied.

**Methods:**

Non-pregnant, HIV-uninfected women aged 18–50 years were enrolled in a dual-centre partially randomized patient-preference interventional trial (NCT04814927) and allowed to choose one of four contraceptive options, etonogestrel contraceptive implant, depot medroxyprogesterone acetate subcutaneous injection, levonorgestrel intrauterine device, and copper IUD, with a minimum target of 12 participants per arm and site. Sampling took place at baseline, 1 and 3 months after contraceptive initiation, and 1 month post-contraceptive discontinuation. Primary outcomes focused on mucosal function and health, while secondary and tertiary outcomes pertained to systemic and mucosal susceptibility to HIV-1 and HSV-2.

**Results:**

Cervicovaginal samples from 112 enrolled participants showed transient, statistically significant changes in endpoints comparing baseline vs. 1 and 3 months after contraceptive initiation. Changes in pH, epithelial thickness, E-cadherin expression, antimicrobial peptides, glycogen, and lactic acid varied with contraceptive type and were typically transient. Serum oestradiol level was correlated with cervicovaginal epithelial function and glycogen and lactic acid secretion. Contraceptive initiation did not result in detectable *ex vivo* or *in vitro* increase of HIV-1 and HSV-2 susceptibility in cellular and tissue models.

**Conclusions:**

Contraceptive initiation during the first 3 months of use induced transient and variable changes in mucosal epithelial and immune markers, not leading to persistent alterations of the vaginal milieu or increased susceptibility to viral infections during the 3 months after contraception initiation.

**Clinical Trial Registration:**

https://clinicaltrials.gov/study/NCT04814927?term=NCT04814927.&viewType=Card&rank=1, identifier NCT04814927.

## 1 Introduction

Globally, nearly half of the 100 million pregnancies that occur annually are unplanned or unintended (1). Safe, effective contraception reduces this global burden, allowing women to space pregnancies and plan their families. Current long-acting contraceptive (LAC) methods provide superior contraceptive efficacy for extended durations and include the progestin-based etonogestrel (ETG) implant, levonorgestrel (LNG) implant, depot medroxyprogesterone acetate (DMPA) injection, and LNG and copper (Cu) IUDs.

The cervicovaginal (CV) mucosal environment plays an important role in health and mucosal defence against disease and pathogens. CV changes may increase susceptibility to sexually transmitted infections, including HIV, herpes simplex virus type-2 (HSV-2), *Trichomonas vaginalis*, *Neisseria gonorrhoeae*, and *Chlamydia trachomatis* (2–4). Exogenous contraceptive hormones and Cu-IUD have been described to affect the CV environment, systemic and local inflammatory levels, and vaginal microbiome in both favourable and adverse ways (5, 6).

Most data on the potential negative impact of exogenous contraceptives have focused on oral contraceptive pills and DMPA intramuscularly administered (DMPA-IM). Multiple and conflicting reports have described an impact on the risk of HIV-1-transmission associated with hormonal contraceptive use, specifically DMPA (7–11). However, the randomized controlled trial “Evidence for Contraceptive Options and HIV Outcomes (ECHO)” found no difference on HIV-1 incidence among women in Sub-Saharan Africa using Cu-IUD, DMPA-IM, or LNG implants for up to 18 months (12). Several studies have provided valuable comparative insights into the public health impact of exogenous contraceptive methods on HIV-1 and HSV-2 incidence as well as other markers of CV health, including mucosal tissue responses, microbial function, mucosal structure, and bacterial STI-susceptibility (11, 13–16). However, no data were collected to establish the baseline CV profile across a broad range of mucosal endpoints (including characterization of the mucosal microenvironment as well as anti-HIV-1 and anti-HSV-2 activities) and understand how each contraceptive potentially modulates this baseline, or even, determine the risk factors of mucosal health alterations induced by contraceptive initiation.

In recent years, new endpoints, including molecular characterization and advanced methodologies, have enabled a better assessment of the impact of contraceptives on women's reproductive health. However, robust data on the mucosal impact of commonly used LACs remain scarce, particularly as these methods are being promoted in populations at high risk for vaginal infections.

We conducted the UMPALA trial to further assess the impact of ETG-CI, DMPA-SC, LNG-IUD, and Cu-IUD initiation on the cervicovaginal environment compared with baseline and to determine how potential changes may affect mucosal function and innate immunity against viral pathogens. All four contraceptive methods selected are safe, highly effective, and widely used. Our choice of DMPA-SC, a new lower-dose DMPA with a different pharmacokinetic profile, allows us to compare results with those of the extensively studied DMPA-IM.

## 2 Materials and methods

### 2.1 Study design and participants

Non-pregnant, HIV-uninfected women aged 18–50 years were recruited at the Clinical Research Center, Eastern Virginia Medical School (EVMS), Norfolk, USA, and the Kenya Medical Research Institute (KEMRI), Thika, Kenya. Enrolment criteria included no systemic disease, having a history of negative Pap smears, intact uterus and cervix, and being able to give written informed consent. Full eligibility and exclusion criteria are described in the protocol (NCT04814927). This was an open-label trial, and participants were enrolled into one of four arms receiving ETG-CI, DMPA-SC, LNG-IUD 52 mg, or Cu-IUD. Study initiation visits (V) consisted of screening and enrolment at (V1) with contraceptive initiation after baseline (V2) sampling. Cu-IUD and LNG-IUD were initiated within 3 weeks of V2. Follow-up visits included V3 and V4, 1 and 3 months after contraceptive initiation, respectively; and V5, 1 month after contraceptive discontinuation. Participants who chose to remain on their form of contraception were exited after V4 and did not return for Visit 5.

### 2.2 Randomization and masking

This study was registered retrospectively: the first participant was enrolled on 11 March 2021; the registration was institutionally submitted to ClinicalTrials.gov on 18 March 2021 and was publicly posted/approved on 23 March 2021, 12 days after the first enrolment. The reason for registration after enrolment began was an internal institutional process delay. Registration was initiated and submitted internally on 4 March 2021 with no changes made to the protocol, outcomes, or statistical plan between enrolment and registration. The authors acknowledge the importance of prospective trial registration and recognize that registration prior to enrolment is best practice.

Participants were enrolled and randomized 1:1:1:1 open-label arms to meet the target sample size of 12–15 evaluable participants per trial arm per site. A random allocation sequence was generated and programmed into REDCap by the study statistician, stratified by site. During the trial, recruitment became difficult and potential study participants pointed to their desire to choose the contraceptive to be used. In May 2021, a protocol amendment was approved to remove randomization and change to a partially randomized patient preference trial, allowing participants to choose their contraceptive option, still with the target of a minimum of 12 participants in each arm per site. EVMS randomized five participants prior to the amendment. All participants at KEMRI were enrolled without randomization. Laboratory personnel and data analysts were blinded until all measurements were completed.

### 2.3 Ethical approvals

Written informed consent was obtained from all participants. The study was conducted in accordance with the International Council for Harmonisation (ICH) guidelines and Good Clinical Practice consistent with the principles of the Declaration of Helsinki. Local ethics approval was received by Advarra® (Pro00045944) for the US site and KEMRI Scientific and Ethics Review Unit (SERU) (KEMRI/RES/7/3/1) as well as the National Commission for Science, Technology and Innovation (NACOSTI/P/21/12811) for the Kenya site. Independent monitoring was conducted at each site for the purposes of safety. No interim analysis was conducted.

### 2.4 Outcomes

Primary outcomes, related to mucosal function and health, included changes from baseline (V2) in free glycogen, pH, lactic acid, Nugent score, concentrations of soluble immune markers in CV secretions, characteristics of the vaginal epithelium (evaluated as epithelial thickness and numbers of layers), and density of immune cells in vaginal tissue. Changes at follow-up visits compared with V2 were indicative of changes after contraception initiation. To determine exposure to semen, we measured a prostate-specific antigen (PSA) in vaginal swabs prior to sampling.

Secondary outcomes, relative to the systemic compartment, included serum oestradiol (E2) and progesterone (P4) concentrations, and HIV-1 p24 levels in peripheral blood mononuclear cells (PBMCs) up to day 14 following *ex vivo* HIV-1_BaL_-challenge [evaluated as p24 concentration at day 14 (p24_d14_) and area-under-the-curve of p24 levels between days 3 and 14 (p24_AUC_)]. An increase or decrease of p24_d14_ and p24_AUC_ at follow-up visits compared with V2 was indicative of increased or decreased susceptibility of PBMCs to HIV-1, respectively.

Tertiary outcomes, related to mucosal susceptibility to pathogens, HIV-1_BaL_ and HSV-2 strain G, were measured by p24 concentrations in ecto-cervical tissue biopsies following *ex vivo* HIV-challenge, and anti-viral activity of CV secretions against HIV-1 and HSV-2 cell infection in *in vitro* models.

### 2.5 Sampling

Study samples were collected at baseline and subsequent visits. Blood, CV secretions obtained as CV fluid (CVF) with Dacron swabs (Puritan Medical, Guilford, ME, USA) (for Nugent score, pH, PSA, and anti-viral activity) or as CV lavage (CVL) (for glycogen, lactic acid, and soluble immune markers), as well as ecto-cervical (only from EVMS) and vaginal biopsies were collected. The samples were immediately transported to the laboratory on ice (median transit time 15 min).

### 2.6 Glycogen, lactic acid, and immune biomarker quantification in CVL

Colorimetric assays were used to measured glycogen (Glycogen assay kit, Abcam, Cambridge, MA, USA) and D- and L-lactic acid (D-/L-lactic acid kit, Megazyme International, Bray, Ireland) levels in CVLs following the manufacturer's instructions. The lower limits of quantification (LLQ) for glycogen, D-lactic, and L-lactic acid were 0.04, 0.01545, and 0.00808 mg/mL, respectively. Concentrations below each assay LLQ were expressed as half-LLQ values. Immune biomarkers were measured in the Genital Tract Laboratory at Brigham and Women's Hospital under accreditation by the College of American Pathologists as previously described (17). Regulated on Activation, Normal T Cell Expressed and Secreted (RANTES) was quantified using the Meso Scale Discovery (MSD) electrochemiluminescence platform (MSD, Gaithersburg, MD), which has been validated for accuracy and precision of cytokine recovery from vaginal fluids using international standards (18). ELISA was used for the quantitation of human β-defensin-2 (HBD2) (Phoenix Pharmaceuticals, Inc., Burlingame, CA) and the secretory leukocyte protease inhibitor (SLPI) (Quantikine, R&D Systems, Minneapolis, MN). The three biomarkers, RANTES, HBD2, and SLPI, were chosen as measures of vaginal health and safety based on established predictive values for the risk of vaginal dysbiosis and sexually transmitted infections, including HIV-1 and HSV-2 acquisition (3, 4, 16, 17).

### 2.7 Vaginal tissue analysis

For histological and immunohistochemistry evaluations, following resection, biopsies were immediately submerged in 10% buffered formalin for 24–48 h. Tissues were then stored at 4 °C in phosphate-buffered saline (PBS) for no more than five days until paraffin embedding, processing, and analysis as described previously (19). Briefly, tissue sections were stained with haematoxylin and eosin to analyse epithelial thickness and layers and with anti-CD45 (Leica biosystems, Nussloch, Germany), anti-CD3 (Leica biosystems), anti-CD8 (Lsbio, Lynnwood, WA, USA), anti-CD1a (Abcam), and anti-HLA-DR (Santacruz Biotechnology, Dallas, TX, USA). Positively stained cells were counted in five to six fields randomly selected under the microscope (Nikon E-800, Nikon, Melville, NY) to quantify immune cell density (cells/mm^2^). E-cadherin expression was reported with manual semi-quantitative scoring based on immunolabelling with an anti-E-cadherin (Abcam) (19). Five to six fields were randomly selected under the microscope for each tissue section. Negative control of staining was based on samples with no primary antibody. Scoring ranged from 0 (no staining), 1 (<10% positively stained cells), 2 (11%–50%), 3 (51%–80%), to 4 (>81%) (20). The scoring was performed blinded by a single histopathologist, with confirmation of counts in a subset of tissues by a second histopathologist. Inter-observer differences resulted in repeat blind counts by both histopathologists.

### 2.8 Isolation of peripheral blood mononuclear cells

PBMCs were isolated from blood collected only at the EVMS site and at V2, V4, and V5 in BD Vacutainer CPT™ tubes containing sodium citrate (Franklin Lakes, NJ, USA) following the manufacturer's instructions. The cells were resuspended in RPMI 1640 (Gibco, Waltham, MA, USA) supplemented (cPRMI) with 10% foetal bovine serum (FBS) (ATCC, Manassas, VA) and antibiotics (100 U of penicillin/mL, 100 μg of streptomycin/mL) (Pen/Strep) (Thermo Fisher Scientific, Waltham, MA, USA).

### 2.9 *Ex vivo* HIV-1-challenge of PBMCs and ecto-cervical tissue

Ecto-cervical biopsies, collected only at the EVMS site, were transported to the laboratory in cRPMI and immediately challenged with HIV-1_BaL_ (5 × 10^4^ TCID_50_) in the presence of IL-2 100 U/mL (Roche Diagnostics GmbH, Indianapolis, IN, USA) (21). After 2–3 h, explants were washed with PBS and cultured for 21 days in the presence of IL-2. PBMCs, collected only from EVMS, were challenged with HIV-1_BaL_ (3 × 10^3^ TCID_50_), washed with PBS the next day, and cultured for 14 days. For ecto-cervical tissue and PBMCs, two-thirds of the culture supernatant were harvested every 3 or 4 days and cultures refed with fresh medium. The extent of virus replication was determined by measuring p24 concentration in supernatants at each harvest time point following the manufacturer's instructions (Alliance HIV-1 p24 Antigen ELISA Kit, PerkinElmer, Waltham, MA, USA). Non-cumulative p24 values were extrapolated using a sigmoidal dose–response (Prism, GraphPad) and reported as p24_final−culture−day_ and p24_AUCrange−days−p24_ (22). Concentrations below the LLQ (12.5 pg/mL) were imputed as half-LLQ.

### 2.10 Innate anti-viral activity of cervicovaginal secretions in cellular models

CVFs were eluted from swabs in 1 mL of 10% FBS, 1% Pen-Strep, and 90% PBS. Activity was tested in TZM-bl cells (ATCC: The Global Bioresource Center|ATCC) (10^4^ cells/well) (23). After overnight culture, the cells were challenged with HIV-1_BaL_ (3 × 10^3^ TCID_50_) in the presence or absence of CVFs (diluted 1:5). Infectivity was measured after 48 h using the Bright-Glo Luciferase Assay System (Promega Corporation, Madison, WI, USA) following the manufacturer's instructions.

For anti-HSV-2 activity, CVFs were eluted from swabs with 400 µL of McCoy's 5A (Gibco) supplemented with 10% FBS and 1% Pen-Strep. After overnight culture of HEC-1-A cells (ATCC: The Global Bioresource Center|ATCC) (10^5^/well), CVFs were added 4 h prior to their challenge with HSV-2 strain G [2,500 plaque-forming units (pfus)]. After 1 h of incubation, the cells were washed with media and cultured for 3 days. The copy number of HSV-2 DNA in the culture supernatant was measured by using qPCR (24).

Anti-viral activities were reported as a percentage of infection vs. internal assay control of infectivity in the absence of CVF.

### 2.11 Bioanalytical methods

Serum concentrations of oestradiol, progesterone, ETG, medroxyprogesterone acetate (MPA), and LNG were measured using a Shimadzu Nexera-LCMS-8050 liquid chromatography-tandem triple quadrupole mass spectrometry (LC-MS/MS) platform (Shimadzu Scientific, Kyoto, Japan) as described previously (25). Briefly, steroids were extracted from samples using supported liquid extraction and concentrations determined by LC-MS/MS. The assay range was 9 pg/mL–10 ng/mL for all hormones except for progesterone, which was 19 pg/mL–10 ng/mL; intra- and inter-assay coefficient of variations were <10% for all assays. Sparse cross-contamination was observed in two participants: in one participant, DMPA-SC was used, in whom LNG was detected, and in one participant in the ETG-CI group, MPA was detected.

### 2.12 Statistical analysis

The planned sample size of 12 participants per arm, per site, was based on feasibility and on differences observed in similar previous mucosal safety studies. This was an exploratory study with a convenient sample to assess the impact of different types of contraceptives on multiple biomarkers. The study was conducted across two sites, enrolling 12 participants per group per site (24 participants per group across two sites). Therefore, for a fixed sample size of 24 participants per group, we had enough power (0.8) to detect a mild-to-moderate effect size (Cohen's d = 0.47) at 5% level of significance with a two-sided *p*-value for primary outcomes.

Baseline characteristics for participants in each arm were summarized using frequency and proportion for categorical variables and median and interquartile range (IqR) for continuous variables. For each endpoint at each visit, the mean change from V2% and 95% confidence interval (CI) was calculated. For each of the quantitative endpoints, to assess for change over time within each group from V2 across V3 to V4, parametric repeated measures ANOVA with a heterogeneous autoregressive covariance structure was performed if normality assumptions were met or on log-transformed data (for data that were not normally distributed and eligible for log-transformation); otherwise, a non-parametric Friedman test for repeated measures was used. For any biomarker, if the main test for repeated measures (ANOVA or Friedman) was significant, a *post hoc* (pairwise) comparison with the appropriate correction was performed to identify the pairs of visits (e.g., V4 vs. V2, V3 vs. V2, or V4 vs. V3) at which change was significantly different, and the adjusted *p*-value was presented. The least squares mean ± standard error (SE) for each visit and the difference between V2 vs. V3 and V2 vs. V4 were determined. The repeated measures ANOVA included contrasts between V3 and V4 compared with V2. The analyses were performed for each site separately and combined.

To test for the difference between the four study groups in any of the quantitative endpoints at each visit, one-way between-subjects ANOVA was used if normality assumptions were met or on log-transformed data. Then, a *post hoc* (pairwise) comparison with Bonferroni correction was performed if a significant difference was noted in the main ANOVA test for any biomarker. Otherwise, a non-parametric one-way between-subjects Kruskal Wallis test was used with a *post hoc* (pairwise) comparison as needed.

To assess the impact of the contraceptive group on the change in any quantitative endpoint at the end of contraceptive use or at 1 month of contraceptive use compared with baseline between the four study groups while adjusting for potential confounders or effect modifiers such as study site, age, race, or Nugent score, multivariable linear regression was used when its assumptions were met; otherwise, median (quantile) regression was used.

The outcome variable of these analyses was calculated as the mean difference of V4 to V2 and the mean difference of V3 to V2 for normally distributed data, and median difference was used for non-normally distributed data. The reported mean difference with SE or median difference with IqR and the associated adjusted *p*-value were based on the adjusted regression models.

For qualitative endpoints, to assess for the change over time within each group from V2 (baseline before starting the contraceptive) to V4 (at the end of contraceptive use) for each qualitative endpoint, a McNamara test for paired proportions was performed.

To test for the difference among the four study groups in any of the qualitative endpoints at each visit, a Chi-squared test for independent samples was performed if the assumptions of the test were met. Otherwise, a Fisher’s exact test was performed.

Then, multivariable binary logistic regression was used to assess for the impact of the contraceptive group on the status of each qualitative endpoint at the end of contraceptive use or at 1 month of contraceptive use compared with baseline while adjusting for potential confounders or effect modifiers such as study site, value at baseline, age, race, or Nugent score. A PSA was used as an objective measure of recent vaginal intercourse; however, other factors that could influence changes, such as sexual activity, condom use, or menstrual phase, were not measured.

Missing data, including loss to follow-up, remained as missing with no attempt to impute. No trends or patterns were identified from the missing data beyond the site difference in completing the optional V5. If outliers were detected as actual data values, a sensitivity analysis was performed to compare the results between the analysis with the actual value and the analysis with outliers being eliminated. Other sensitivity analyses were conducted to exclude PSA positives, the five randomized participants, observations with protocol deviation, or participants with hormonal contamination, and the findings remained the same.

Analyses were performed in SAS v7.4 and STATA 17. A correlation analysis was performed in R statistical software (v4.1.2; R Core Team 2021), and correlation matrices between endpoints were constructed using Pearson correlation tests with two-tailed *p*-values.

### 2.13 Role of the funding source

Funders had no role in the processes of data collection, data analysis, or data interpretation. In addition, USAID had no role in the study design or writing of the report. All authors had full access to all the data in the study and had final responsibility for the decision to submit for publication.

## 3 Results

### 3.1 Demographics and safety

Between March 2021 and July 2022, 194 women were screened, of whom 82 did not meet the eligibility criteria, leaving 112 enrolled participants, 52 at the EVMS and 60 at the KEMRI (Figure 1). Two participants withdrew from the study. KEMRI participants who attended V2 prior to contraceptive initiation had different baseline characteristics (Table 1) from those at the EVMS in terms of age [median (IQR): 27 (23–36) at the KEMRI vs. 40 (28–45) at the EVMS, *p* < 0.001], BMI, with more overweight and obese women at the EVMS (*p* < 0.001), and race (56.9% were Caucasian at the EVMS; *p* < 0.001). One participant was discontinued from the study after she reported a positive pregnancy test result at V3 and another after V3 due to IUD expulsion. Five participants did not complete the study because they were lost to follow-up. Five participants were excluded from specific data points because of the use of trial-excluding medications (three at V3 and two at all visits for HIV-1 and HSV-2 endpoints). Two participants missed V4. Twenty-three participants voluntarily chose to discontinue the product and completed V5, mostly from the EVMS site (Figure 1, Supplementary Table S1). Because of the majority of participants continuing with the chosen contraceptive, V5 results were not powered for statistical analysis. Positive PSA test results, indicative of residual semen, were obtained in 9.82% of participants at V2, 14.28% at V3, 6.25% at V4, and in none of the 23 participants who returned to V5 after contraceptive discontinuation (Supplementary Table S2). The enrolled participants tested negative for *Neisseria gonorrhoea*, *Chlamydia trachomatis*, and *Trichomonas vaginalis*. No severe adverse events were reported for any of the contraceptives tested (Supplementary Table S3).

**Figure 1:**
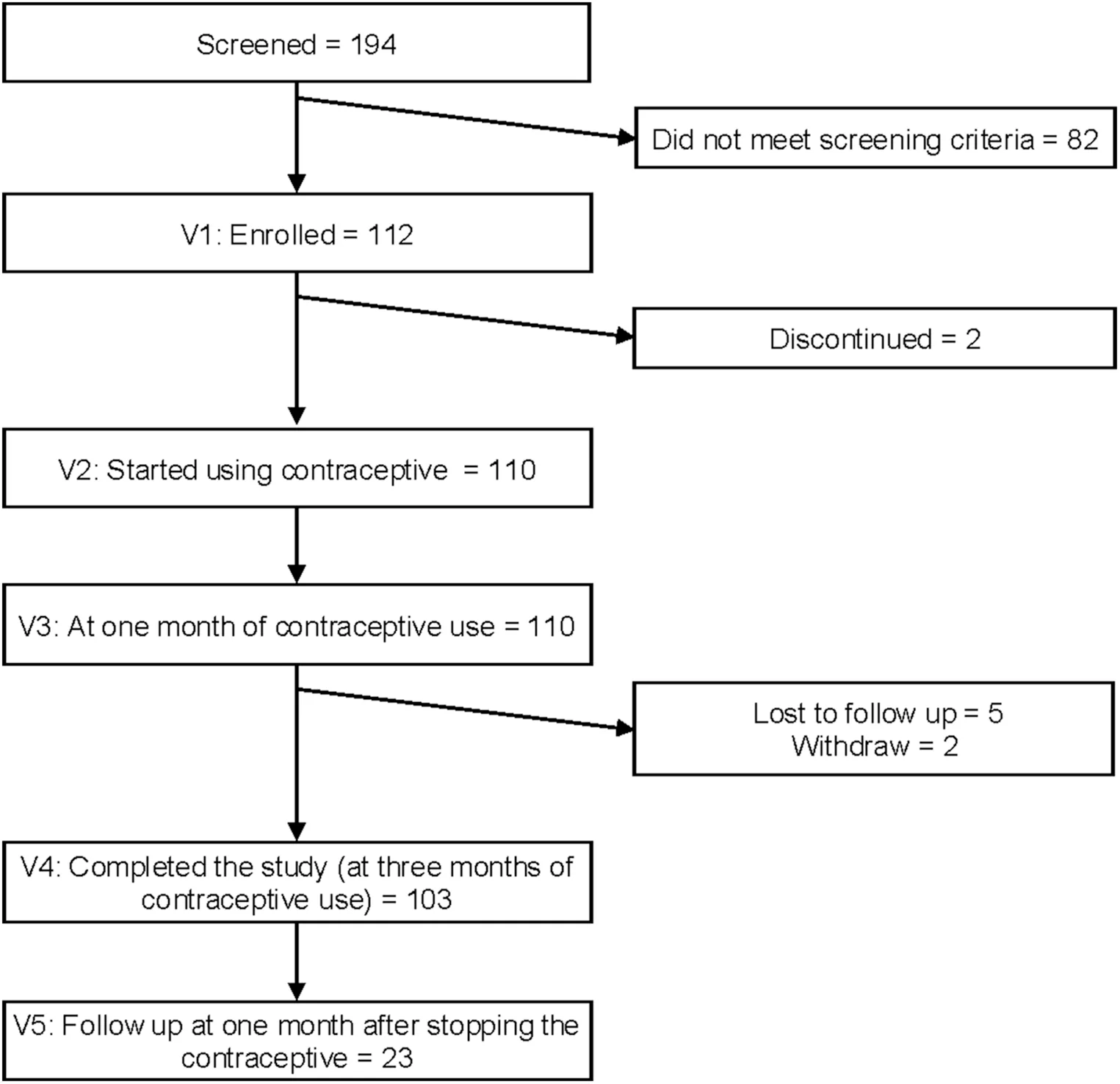
Recruitment of trial participants and follow-up.

**Table 1:** Baseline characteristics of UMPALA trial participants.^a^

Characteristics	Total	EVMS	KEMRI	*p*-value
Participants per site, *N* (%)		51 (46.36)	59 (53.64)	
Age, median (IqR)	29.5 (25–41)	40 (28–45)	27 (23–36)	<0.001^b^
Ethnicity,^c^ *N* (%)				N/A
Hispanic	_	5 (9.8)	_	
Non-Hispanic	_	46 (90.20)	_	
Race				<0.001^d^
African	59 (53.64)	0 (0.00)	59 (100.00)	
African American	22 (20.00)	22 (43.14)	0 (0.00)	
White	29 (26.36)	29 (56.86)	0 (0.00)	
BMI, *N* (%)^e^				<0.001^d^
Normal weight	36 (32.73)	7 (13.73)	29 (49.15)	
Overweight	34 (30.91)	16 (31.37)	18 (30.15)	
Obesity	40 (36.36)	28 (54.90)	12 (20.34)	
Education level, *N* (%)				<0.001^d^
Less than high school	17 (15.45)	0 (0.00)	17 (28.81)	
High school/GED	30 (27.27)	9 (17.65)	21 (35.59)	
Some college	33 (30·00)	16 (31.37)	17 (28.81)	
Associate's degree	10 (9·09)	10 (19.61)	0 (0.00)	
Bachelor's degree	13 (11.82)	9 (17.65)	4 (6.78)	
Master's degree	6 (5.45)	6 (11.76)	0 (0.00)	
Doctoral degree	1 (0.91)	1 (1.96)	0 (0.00)	
Marital status, *N* (%)				<0.001^d^
Never married	53 (48.18)	25 (49.02)	28 (47.46)	
Married	29 (26.36)	17 (33.33)	12 (20.34)	
Separated	16 (14.55)	1 (1.96)	15 (25.42)	
Divorced	8 (7.27)	8 (15.69)	0 (0.00)	
Widowed	4 (3.64)	0 (0.00)	4 (6.78)	

a Characteristics for the 110 participants who attended visit 2.

b *p*-value calculated using a non-parametric Kruskal Wallis test.

c Ethnicity of participants at the EVMS site.

d *p*-value calculated using a Chi-squared test.

e *p*-value calculated using a non-parametric Fisher's exact test. BMI range (kg/m^2^): normal weight between 18.5 and <25; overweight between 25 and <30; obesity ≥ 30. N/A, not applicable.

### 3.2 Systemic PK

For hormonal contraceptives, serum levels of 0.36 ± 0.022 ng/mL ETG in the ETG-CI group, 0.31 ± 0.035 ng/mL MPA in the DMPA-SC group, and 0.28 ± 0.056 ng/mL LNG in the LNG-IUD group were detected at V4 (Supplementary Figure S1E, Supplementary Table S4). Decreases of systemic E2 and P4 levels compared with V2 were observed with ETG-CI ([E2] = 45.44 ± 46.04 pg/mL, *p* < 0.001 at V3, [E2] = 61.06 ± 71.95 pg/mL, *p* = 0.043 at V4; [P4] = 0.30 ± 0.52 pg/mL, *p* = 0.226 at V3, [P4] = 0.16 ± 0.10 pg/mL, *p* = 0.006 at V4) and DMPA-SC ([E2] = 38.08 ± 36.81 pg/mL, *p* = 0.003 at V3, [E2] = 51.04 ± 37.00 pg/mL, *p* = 0.307 at V4; [P4] = 0.16 ± 0.14 pg/mL, *p* = 0.008 at V3, [P4] = 0.13 ± 0.076 pg/mL, *p* < 0.001 at V4) (Figures 2A,B and Supplementary Table S5). For the 23 participants who attended V5, changes in E2 and P4 levels after discontinuation of contraceptives varied for each participant (Supplementary Figure S2).

**Figure 2:**
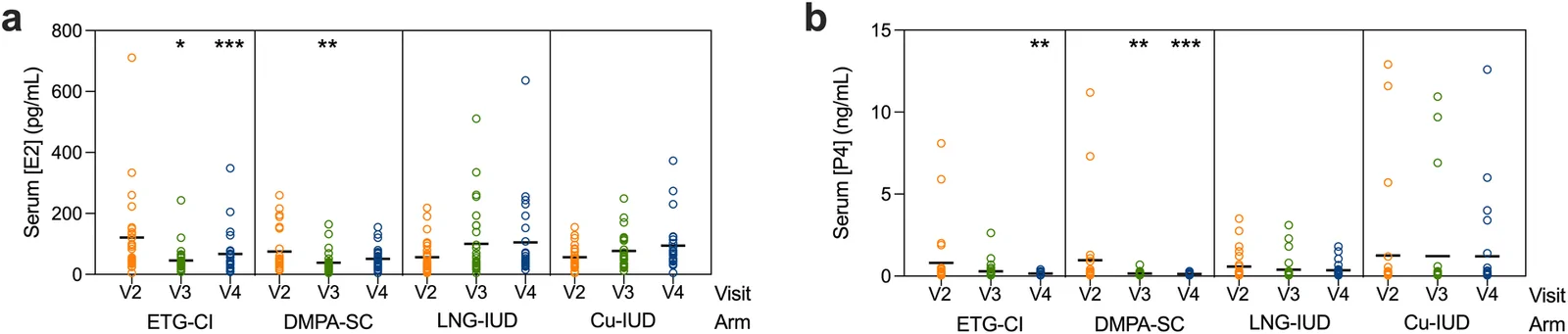
Serum levels of estradiol and progesterone. Concentrations of E2 **(a)** and P4 **(b)** at each visit in each trial arm are shown for both sites combined. * Indicates statistical difference vs. V2.

### 3.3 Endpoints for each contraceptive

#### 3.3.1 ETG-CI

Among the primary outcomes of mucosal function measured, ETG-CI initiation induced decreased CV secretion of the immune marker SLPI compared with baseline levels (LSMs difference=−0.3076 ± 0.1369, *p* = 0.033 at V3, LSMs difference=−0.2879 ± 0.1193, *p* = 0.024 at V4). Considering individual sites, SLPI modulation reached significance at V4 among EVMS participants (LSMs difference=−0.3309 ± 0.1524, *p* = 0.049) (Figure 3g, Supplementary Figure S3M, N, Supplementary Table S6).

Associated with mucosal function, transient changes in vaginal tissue were also observed. ETG-CI use induced an initial decrease of vaginal epithelial thickness (LSMs difference=0.81 ± 1.08, *p* = 0.021) and number of layers at V3 (LSMs difference=0.84 ± 1.05, *p* = 0.001), followed by an increase of both thickness (*p* < 0.001) and layers (*p* < 0.001) at V4 compared with V3, returning to levels similar to those measured at V2 (thickness: LSMs difference=1.09 ± 1.08, *p* = 0.352; number of layers: LSMs difference=1.05 ± 1.0, *p* = 0.723) (Figure 4, Supplementary Figure S5, Supplementary Table S7). However, with this contraceptive, opposite patterns of expression were observed for the tight-junction protein E-cadherin, with a slight increase of the E-cadherin score at V3 (*p* = 0.045) and a decrease at V4 (*p* = 0.045), and for the levels of epithelial CD1a ^+^ cells, which significantly decreased at V4 (LSMs difference=0.76 ± 1.11, *p* = 0.019) (Figure 4; Supplementary Figure S5, Supplementary Table S7).

**Figure 3:**
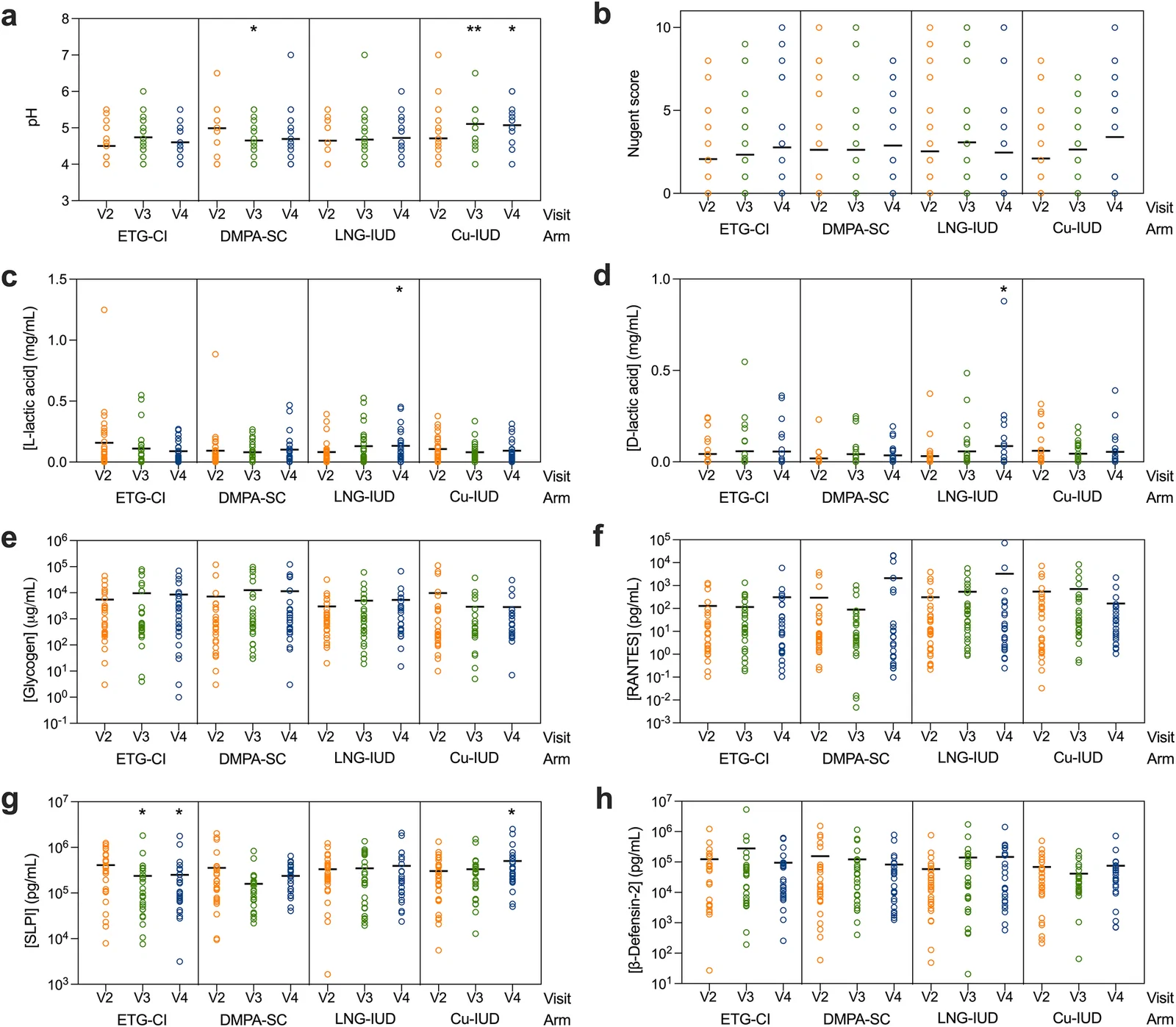
Vaginal pH, Nugent score, and levels of L-lactic acid, D-lactic acid, glycogen, RANTES, SLPI, and β-defensin-2 in cervicovaginal secretions. Concentrations of pH (**a**), Nugent score (**b**), L-lactic acid (**c**), D-lactic acid (**d**), glycogen (**e**), RANTES (**f**), SLPI (**g**), and β-defensin-2 (**h**) from each participant are shown at each visit per trial arm for both sites combined. * Indicates statistical difference vs. V2.

With regard to the effect of CVF on *in vitro* cellular models of infection by viral STI pathogens, overall, no significant changes in infection levels for both HIV-1_BaL_ and HSV-2 strain G were observed with CVFs from participants using ETG-CI (Figure 5; Supplementary Figure 10, Supplementary Table S9). In an *ex vivo* ecto-cervical tissue explant infection model, the susceptibility of biopsies to HIV-1_BaL_ was not altered by ETG-CI initiation (Figure 5, Supplementary Figures S10, 11, Supplementary Table S9, 11). In contrast, systemically, a significant decrease in the *ex vivo* susceptibility to HIV-1_BaL_ was measured in PBMCs isolated from participants using ETG-CI, with lower p24_AUCd3−d14_ values at V4 compared with V2 (LSMs difference=−0.6354 ± 0.2532, *p* = 0.026) (Supplementary Figure 1^1^, Supplementary Table S10).

**Figure 4:**
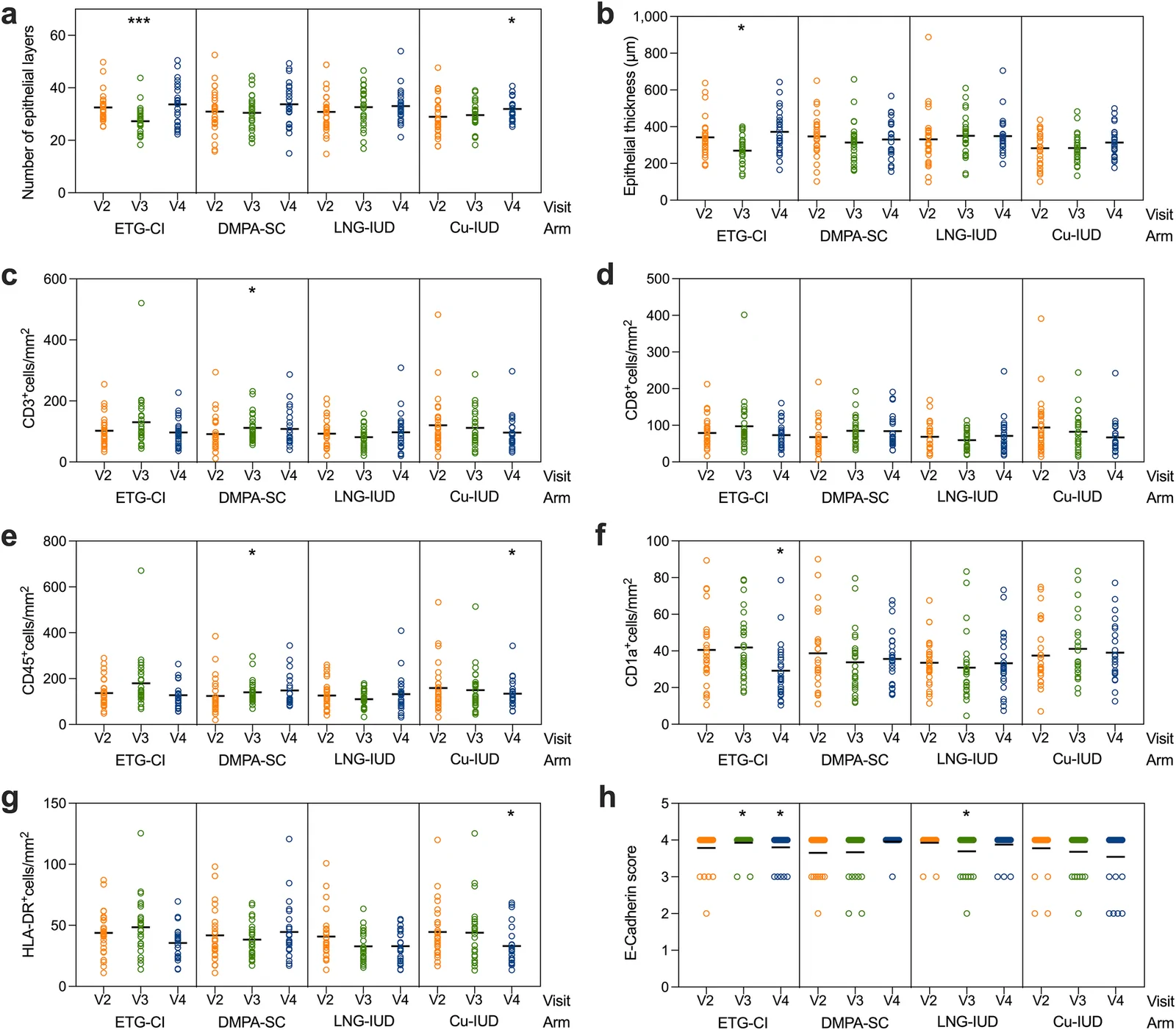
Immunohistochemical analysis of vaginal epithelium. Number of layers (**a**) and thickness of epithelium (**b**), as well as immune cell densities, CD3^+^ cells (**c**), CD8^+^ cells (**d**), CD45^+^ cells (**e**), CD1a^+^ cells (**f**), HLA-DR+ cells (**g**); and tight junction evaluation, via E-cadherin scoring (**h**), were assessed for each participant at each visit per trial arm. Data shown are for both sites combined. * Indicates statistical difference vs. V2.

No consistent changes for any endpoints measured were observed among the participants who discontinued ETG-CI use (Supplementary Figures S4, 8, 9, 12).

#### 3.3.2 DMPA-SC

For primary outcomes, 1 month after DMPA-SC initiation (V3), a significant decrease (*p* = 0.036) in CV pH was observed, which was not fully restored after 3 months of contraceptive use (V4) (Figure 3a, Supplementary Table S6). This pH modulation was not associated with changes in Nugent score nor in lactic acid or glycogen levels.

**Figure 5:**
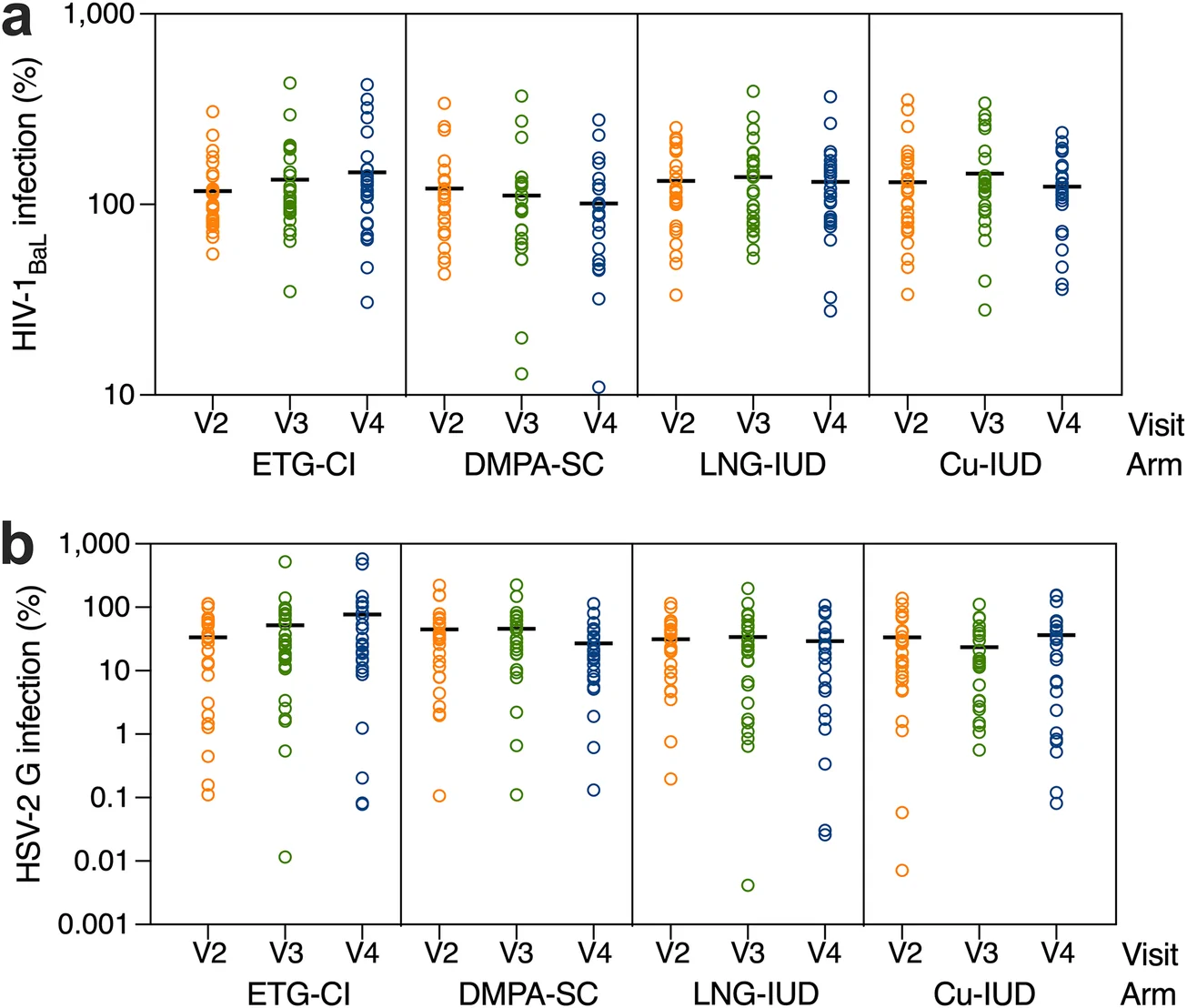
Impact of CVFs on *in vitro* HIV-1_BaL_ and HSV-2 strain G infection levels of cellular models. Infection levels of TZM-bl cells for HIV-1_BaL_ (**a**), and HEC-1A cells for HSV-2 strain G (**b**), in the presence of CVFs from participants at each visit per trial arm.

At the immunohistochemical level, DMPA-SC was linked to a progressively increased number of CD45 ^+^ cells and CD3 ^+^ cells in the vaginal lamina propria, reaching overall significance after 3 months of use (CD45^+^: *p* = 0.026; CD3^+^: *p* = 0.042 at V4) mainly driven by significant differences among KEMRI participants for CD45^+^: *p* = 0.037; CD3^+^: *p* = 0.014) (Supplementary Figures S6, 7, Supplementary Table S8).

No changes in viral mucosal and systemic susceptibility were observed during the first 3 months post-DMPA-SC initiation (Figure 5, Supplementary Figures S10, 11, Supplementary Tables S10, 11).

Among the participants who discontinued DMPA-SC, no consistent impact of contraceptive cessation was observed for the different endpoints measured (Supplementary Figures S4, 8, 9, 12).

#### 3.3.3 LNG-IUD

With regard to primary outcomes associated with mucosal function, the use of LNG-IUD for 3 months was, for both sites combined, linked to increased mucosal secretion levels of L- and D-lactic acid (Figure 3c,d, Supplementary Table S6), with no significant impact on Nugent scores. The levels of RANTES in CVFs from the participants of both sites combined did not show any differences upon LNG-IUD initiation. However, site-specific changes were observed for this chemokine at V4 (EVMS: LSMs difference=0.9887 ± 0.3690, *p* = 0.025; KEMRI: LSMs difference=−0.8174 ± 0.3498, *p* = 0.035) (Supplementary Figure S3M, N, Supplementary Table S6). LNG-IUD was the only contraceptive that induced a trend toward increased HBD2 levels after 3 months of use (V4), which reached significance only among KEMRI participants (LSMs difference=0.8975 ± 0.2107, *p* < 0.001) (Supplementary Figure S3P, Supplementary Table S6).

The initiation of this hormonal contraceptive did not induce CV immunohistochemical changes.

LNG-IUD did not affect the *ex vivo* and *in vitro* susceptibility to HIV-1 or HSV-2 in the cellular and tissue models used (Figure 5, Supplementary Figures S10, 11, Supplementary Tables S10, 11).

With very few participants choosing not to continue using LNG-IUD, at V5, no discontinuation effect could be observed for each of the endpoints measured (Supplementary Figures S4, 8, 9, 12).

#### 3.3.4 Cu-IUD

Compared with V2, among the primary outcomes of mucosal function, CV pH increased with Cu-IUD initiation (*p* = 0.004 at V3, *p* = 0.044 at V4) especially among EVMS participants with *p* = 0.008 at both visits (Figure 3a, Supplementary Figure S3A, Supplementary Table S6). Increased SLPI levels were also observed after 3 months of Cu-IUD use (LSMs difference=0.2661 ± 0.1088, *p* = 0.022 at V4) (Figure 3g, Supplementary Table S6). For individual sites, significance at V4 was reached only among KEMRI participants (LSMs difference=0.2988 ± 0.1211, *p* = 0.027) (Supplementary Figure 3K, L, Supplementary Table S6).

An evaluation of vaginal tissue immunohistochemical properties showed that, at V4, a significant decrease in epithelial CD45^+^ cell density (LSMdif=0.88 ± 1.12, *p* = 0.044) and a decreasing trend in the density of CD8^+^  T cells (LSMdif=0.75 ± 1.14, *p* = 0.162) were linked to a significant increase in the number of epithelial layers (LSMdif=1.10 ± 1.05, *p* = 0.048) with no observed significant changes in thickness (Figure 4, Supplementary Figure S5, Supplementary Table S7). This Cu-IUD-induced decrease on epithelial immune cells was also observed in the lamina propria with an overall trend towards a decrease in CD45^+^ (LSMdif=0.75 ± 1.12, *p* = 0.124), HLA-DR^+^ (LSMdif=0.77 ± 1.11, *p* = 0.125), and CD8^+^ cell density (LSMdif=0.63 ± 1.16, *p* = 0.090).

No significant increases of *ex vivo* and *in vitro* susceptibility to HIV-1 or HSV-2 were observed within the first 3 months of Cu-IUD initiation (Figure 5, Supplementary Figures S10, 11, Supplementary Tables S10, 11).

For participants who decided not to continue using Cu-IUD after V4, individual changes were observed for the endpoints measured 1 month after contraceptive discontinuation (V5) (Supplementary Figures S4, 8, 9, 12).

### 3.4 Correlation analysis of endpoints

With no specific contraceptive inducing significant changes across all endpoints analysed, we performed a correlation analysis between all endpoints focused at each visit after contraception initiation independently of the contraceptive used (Figure 6, Supplementary Figure S1^3^, Supplementary Table S1^1^), rather than per visit and study group (Supplementary Figure S1^4^). For all visits, *in vitro* HIV-1 infection levels were significantly positively correlated with vaginal pH (at V2: *p* < 0.0001, *r* = 0·4259; V3: *p* = 0.0451, *r* = 0.196; V4: *p* = 0.0173, *r* = 0.2387) and Nugent score (at V2: *p* < 0.0001, *r* = 0.4132; V3: *p* = 0.0001, *r* = 0.3717; V4: *p* < 0.0001, *r* = 0.4575). Interestingly, HIV-1 levels were significantly inversely correlated with CV levels of glycogen (at V2: *p* = 0.0443, *r* = −0.1939; V3: *p* = 0.011, *r* = −0.2484; V4: *p* = 0.0119, *r* = −0.2544) and L- (at V2: *p* = 0.0046, *r* = −0.2697; V3: *p* = 0.0257, *r* = −0.2177; V4: *p* = 0.0018, *r* = −0.3098) and D-lactic acid (at V2: *p* = 0.017, *r* = −0.2282; V3: *p* = 0.0316, *r* = −0.2099; V4: *p* = 0.0029, *r* = −0.2963). At baseline, *in vitro* HSV-2 infection levels directly correlated with vaginal epithelial thickness (*p* = 0.0458, *r* = 0.1944) and inversely correlated with vaginal L-lactic acid (*p* = 0.0259, *r* = −0.2143). However, the statistical significance of these correlations was lost with contraceptive initiation. Serum E2 levels directly correlated with SLPI levels in CVLs at each visit (at V2: *p* < 0.0001, *r* = 0.3893; V3: *p* = 0.0055, *r* = 0.2692; V4: *p* = 0.0392, *r* = 0.2066). Vaginal epithelium thickness and number of epithelial layers directly correlated with serum E2 levels, without reaching statistical significance, and significantly inversely correlated with the density of epithelial immune cells (Figure 6, Supplementary Figure S1^3^, Supplementary Table S1^1^). Significant positive correlations between vaginal SLPI levels and L-lactic acid levels were observed at baseline and after contraception initiation (at V2: *p* = 0.0047, *r* = 0.2686; V3: *p* < 0.0001, *r* = 0.3974; V4: *p* = 0.0001, *r* = 0.3768). Similarly, we found direct correlations between the SLPI and D-lactic acid (at V3: *p* < 0.0001, *r* = 0.5218; V4: *p* = 0.0013, *r* = 0.3163) or HBD2 (at V3: *p* = 0.0292, *r* = 0.213; V4: *p* = 0.0004, *r* = 0.3458) levels but reaching statistical significance only at V3 and V4. RANTES levels in CVLs directly correlated with vaginal pH, reaching significance at baseline and after 1 month of contraceptive use (V3) (at V2: *p* = 0.0002, *r* = 0.3481; V3: *p* = 0.0111, *r* = 0.247; V4: *p* = 0.1239, *r* = 0.1549).

**Figure 6:**
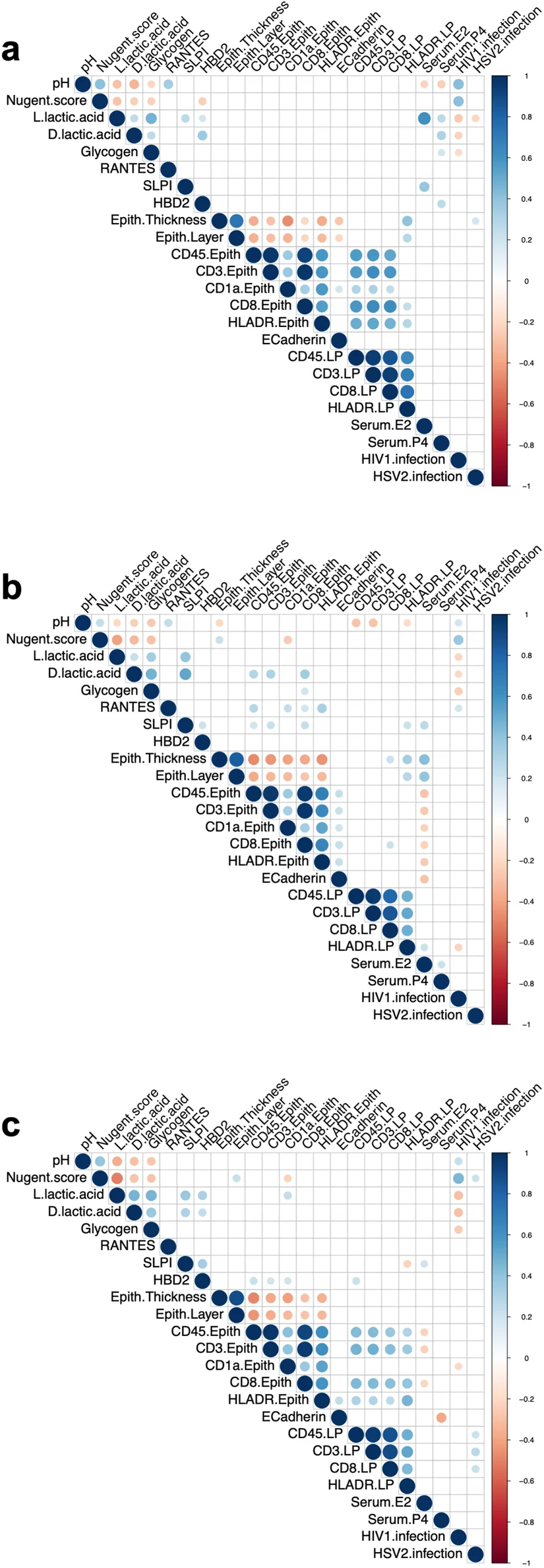
A correlogram of UMPALA endpoints. Significant (*p* < 0.05) correlations between endpoints measured at **(a)** V2, **(b)** V3, or **(c)** V4 independently of the contraceptive used. The colour intensity of positive (blue) and negative (red) correlation coefficients shown in the sidebar is proportional to the correlation coefficient. The circle size is proportional to the statistical significance of the correlation.

## Discussion

UMPALA was an open-label trial that included four approved contraceptives in Kenya and the United States, aiming to assess the impact of contraceptive use on the lower genital tract of women after 1 and 3 months of product initiation. Although transient biological changes were observed in certain endpoints during the first 3 months following initiation, contraceptives did not persistently alter the markers of mucosal function and health analysed in this study (Figure 7). Furthermore, contraceptive initiation was not associated with detectable increases in *ex vivo* or *in vitro* HIV-1 and HSV-2 susceptibility over the first 3 months of use based on the cell- and tissue-based models employed in this study, including *ex vivo* (performed with PBMCs and ecto-cervical biopsies collected at the EVMS) and *in vitro* assays (conducted with samples from both sites).

**Figure 7:**
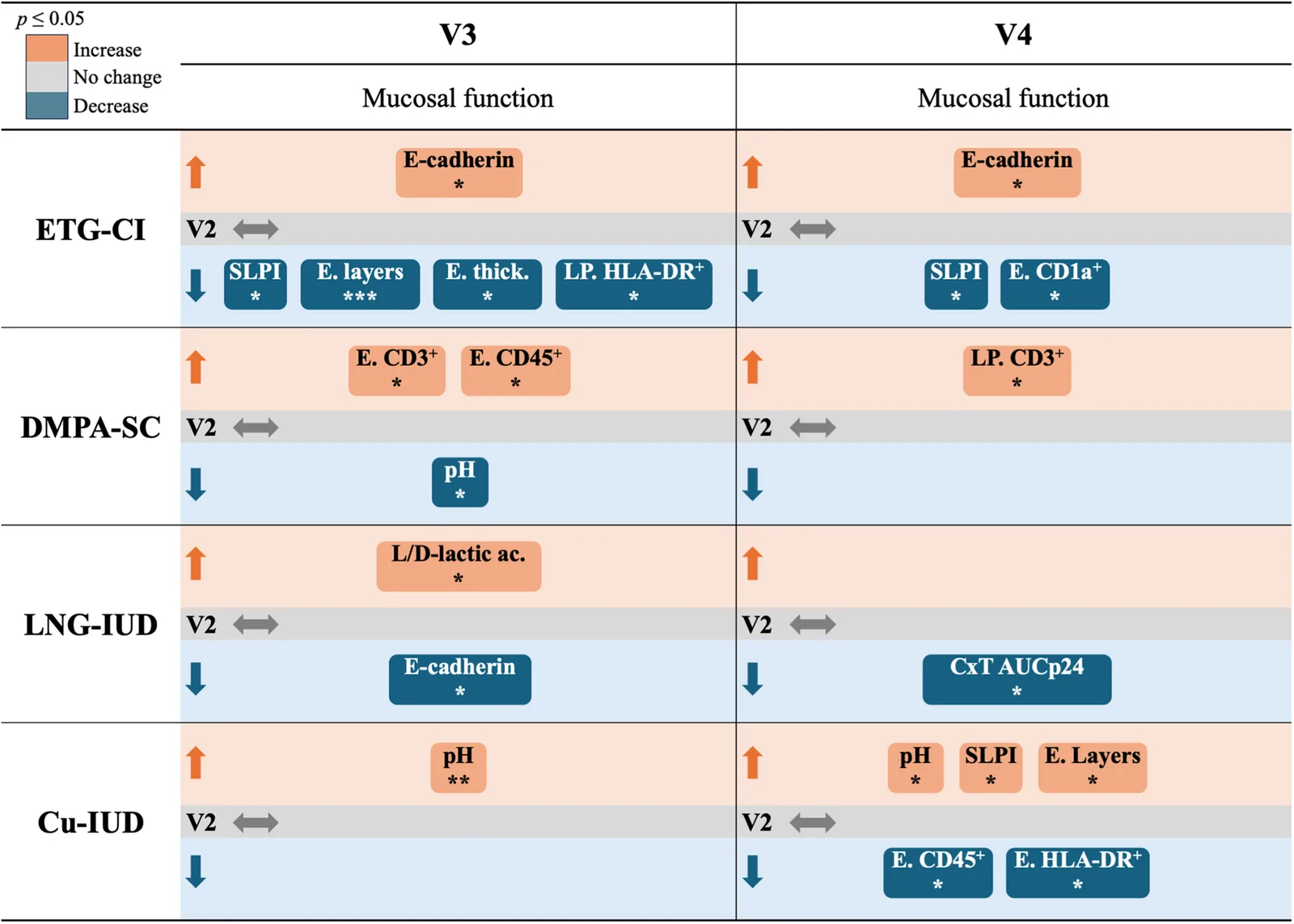
Effect of contraceptives on the cervicovaginal compartment. Statistically significant increased (orange) or decreased (blue) endpoints are shown for each contraceptive at V3 and V4 compared with V2 (grey: no change). E, epithelium; LP, lamina propria; CxT, ecto-cervical tissue. * Indicates statistical difference vs. V2. Endpoints not significantly modulated are not shown.

Previous trials have described CV inflammatory responses linked to the initiation of certain contraceptives, especially DMPA-IM (11, 26–30). Mucosal inflammatory changes and changes in antimicrobial peptides (AMPs) such as the SLPI were also reported in two sub-studies of women from the large ECHO trial conducted in Sub-Saharan Africa, specifically in those randomized to Cu-IUD compared with LNG implant (27, 30). However, in that same trial, these two contraceptives and DMPA-IM did not differ in susceptibility to HIV-1 acquisition (27). The trial did not have a no-contraceptive control arm and therefore could not clearly establish whether the contraceptives increased the risk of transmission over baseline. A cross-sectional study performed among Kenyan and South African women showed that the use of DMPA-IM was associated with an increase in RANTES levels in CV secretions (26). Majority DMPA-IM use was associated with increased RANTES and decreased HBD2, and majority combined oral contraception use was associated with increased SLPI in the Hormonal Contraception -HIV study in Uganda and Zimbabwe (29). RANTES and AMPs, the SLPI and HBD2, were associated with the modulation of susceptibility to HIV-1 and HSV-2 in the same study (16, 29). In contrast, inflammatory responses, including increase of RANTES CV secretion levels, were not observed with DMPA-IM initiation within the Zim CHIC trial conducted in Zimbabwe (11). In that same study, RANTES was elevated among Cu-IUD users 1-month post-initiation, but this increase was not maintained at the 6th month. In the CONRAD A10–114 trial, conducted in the US and Dominican Republic, injectable DMPA-IM, in general, induced a CV anti-inflammatory response and decreased secreted levels of the SLPI six weeks after contraception initiation (28). In the UMPALA trial, conducted in Kenya and the US and reported here, the initiation of DMPA-SC did not modulate mucosal secretion levels of RANTES, HBD2, or the SLPI within the short period of 3-month use. However, the SLPI showed a sustained decrease at 1 and 3 months after ETG-CI initiation and an increase after 3 months of Cu-IUD use when the Kenyan and US sites were pooled together. This statistically sustained increase of the SLPI was not associated with an *in vitro* increase in HIV-1 or HSV-2 susceptibility, suggesting that, despite reaching statistical significance, the change was not biologically impactful. Statistical significance for RANTES and HBD2 changes was reached only when each site was analysed independently with opposite directions observed by site. This could be due to the different median ages of the two cohorts, with younger participants at the KEMRI, or interaction with other unexamined factors specific to their geographical locations and backgrounds.

Few studies have assessed the impact of contraceptives on the CV mucosal tissue, and most of them have focused on injectable DMPA-IM (13, 28, 31). In general, these studies report no changes in vaginal epithelium thickness or number of epithelial layers, with increased immune cell density in epithelium and lamina propria, as well as enhanced cervical mucosal barrier function during initial months after DMPA initiation. Bunjun et al. reported an increase in CV Th17-like cells in women using DMPA-IM compared with those using Cu-IUD or LNG-IUD (13). However, no changes in cervical HIV-target cells were observed with any of the contraceptives tested in the Zim CHIC study (11). No changes in CV HIV target cells were reported in the CHIME study either comparing DMPA-IM, ETG-CI, and LNG-IUD (32). In a US cohort, after 1 year of DMPA-IM use, a decreased number of CD1a^+^ cells and infiltrating CD3^+^ T cells in vaginal biopsies was reported (33). In contrast, an analysis of ecto-cervical biopsies from a DMPA-experienced cohort from Kenya indicated impaired epithelium integrity with the migration of CD4^+^ T cells from the lamina propria towards the epithelium (34). In our multicentre UMPALA trial, we observed a progressive increase of CD45^+^ cells and CD3^+^ T cells in the vaginal lamina propria of women in the DMPA-SC group, reaching significance after 3 months (V4) (CD45^+^: *p* = 0.026; CD3^+^: *p* = 0.042). Changes in the vaginal epithelium of biopsies from Cu-IUD users were observed only at V4, while ETG-CI initiation first decreased but later restored the number of epithelial layers and thickness. Changes in the epithelium correlated with changes in SLPI levels.

None of the contraceptive methods tested in this trial significantly increased CV *ex vivo* susceptibility to HIV-1 or HSV-2 in comparison with the other contraceptives or baseline, which, in part, aligns with the results from the ECHO trial (12). Further evaluation of the mucosal environment during the UMPALA trial revealed that CV lactic acid levels measured at each visit, and independently of the contraceptive group, correlated positively with SLPI levels and negatively with *in vitro* HIV-1_BaL_ infection in the presence of CVFs, confirming the protective role of lactic acid reported in the literature (35). The CV environment profiles of UMPALA participants with mucosal L- and D-lactic acid levels in top tertiles at V3 and V4, and top tertile mucosal concentrations for SLPI and HBD2, were associated with HIV-1 infection levels of TZM-bl cells in the low tertiles, i.e., they were protective. None of the contraceptives tested induced a significant decrease in CV lactic acid, which further supports the ECHO's *in vivo,* and this study’s *ex vivo*, results showing no significant impact on CV susceptibility to HIV-1 or HSV-2. Furthermore, the transient pH increase observed with Cu-IUD at V3 (*p* = 0.004), and at V4 with less significance (*p* = 0.044), is a possible consequence of inconsistent microbiome modulation as reported in previous studies during Cu-IUD initiation (30, 36, 37). However, this pH increase was not associated with changes in HIV-1 or HSV-2 susceptibility in this group. Transient biological changes of the CV environment have also been reported with LNG-IUD initiation (38).

Serum E2 levels showed a positive correlation with SLPI, confirming previous findings in the hypoestrogenic CV environment of post-menopausal women (19, 39). This raises the question about whether the conflicting results among trials evaluating the effect of DMPA and other progestins may be linked to their effects on serum E2 levels. Compared with serum E2 levels following DMPA-SC administered during the UMPALA trial, DMPA-IM has been shown to induce greater decreases in the first months after contraception initiation (40, 41). Based on the findings and correlations described above, we speculate that the effect of contraceptives on the CV environment may be mediated by their impact on systemic and local E2 levels, which, in turn, may be associated with epithelial function and glycogen and AMP (e.g., SLPI) secretion (42). Both glycogen and AMPs regulate the growth of lactobacilli and a polydisperse anaerobic microbiota, a balance that determines whether a dysbiotic state such as bacterial vaginosis may be established (42–44). A higher estrogenic environment stimulates epithelial differentiation and integrity, as well as function, increasing the secretion of glycogen and AMPs, growth of lactobacilli, and production of lactic acid and protons, which maintain the vaginal pH at low physiological levels (42). This CV milieu has been associated with vaginal health and decreased susceptibility to vaginal infections (42).

Key strengths of the study design are the inclusion of baseline sampling for each participant to assess mucosal changes induced by each contraceptive, enrolment of two separate populations of women from the US and Kenya, the use of *ex vivo* models to evaluate susceptibility to HIV-1 and HSV-2, and measurement of multiple CV endpoints before and after contraceptive initiation.

This study had some limitations. First, soon after starting randomization, it was changed to partial randomization with patient preference as women strongly preferred choosing their contraceptive. This might lead to selection bias. Contraceptive choice has been reported in other studies, and in this study, it did not affect the conclusions, as demonstrated by sensitivity analyses and the lack of significant baseline differences among the study arms. Second, this was an open-label study where only laboratory staff was blinded. Laboratory endpoints, however, were the most important ones in this study. Third, the inclusion age range was broad, resulting in a younger cohort in one site, Kenya, compared with the other one, US, and therefore, it was not generalizable to all populations. Fourth, a targeted proteomic analysis was conducted instead of a broad panel of pro- and anti-inflammatory cytokines/chemokines being measured. Fifth, *ex vivo* HIV-1_BaL_ susceptibility of PBMCs and ecto-cervical tissue explants was evaluated only at the EVMS site because of logistic restrictions in the Kenyan site, and PBMCs were not collected for this endpoint at V3. Furthermore, to avoid possible complications linked to biopsy collection, an immunohistochemical analysis was performed in vaginal tissue, while the *ex vivo* HIV-1_BaL_ challenge was implemented on ecto-cervical biopsies. Sixth, susceptibility to HIV-1 and HSV-2 was assessed with *ex vivo* and *in vitro* models instead of clinical endpoints. Hence, future trials will need to clinically evaluate the impact of transient changes during the first months after contraceptive initiation on HIV/HSV acquisition. In addition, with a limited number of participants, mostly at the EVMS site, deciding to discontinue contraceptives after 3 months of use, no conclusion could be drawn on the impact of contraceptive cessation on the mucosal environment. Finally, this study was designed to assess mucosal responses to contraceptive use during a limited period following initiation, the first 3 months of use; hence, larger studies are needed to evaluate responses to long-term contraceptive use.

In an era where contraceptive uptake is essential to prevent the high levels of unintended pregnancies worldwide, side effects and health risk around contraception are still influencing the choice and use of contraceptives. Overall, the UMPALA trial showed that contraceptive initiation induced transient alterations in selected cervicovaginal biomarkers without evidence of sustained mucosal disruption or increased viral susceptibility during the first 3 months through surrogate models of *in vitro* infection, which is of particular importance in populations at high risk of HIV-1 and HSV-2.

## Data Availability

The raw data supporting the conclusions of this article will be made available by the authors upon request, without undue reservation.
