# Supplementary material for: Impact of contraceptive initiation on the cervicovaginal mucosa: results from the UMPALA multicentre, partially randomized patient-preference interventional trial
Source: Front Reprod Health. 2026 Oct 2;8:1882772. doi: 10.3389/frph.2026.1882772 (PMC13672642; doi:10.3389/frph.2026.1882772)
Supplement: Supplementary file 1 [file Supplementaryfile1.pdf]

frontiers

Supplementary Material

1 Supplementary Figures and Tables

1.1 Supplementary Figures

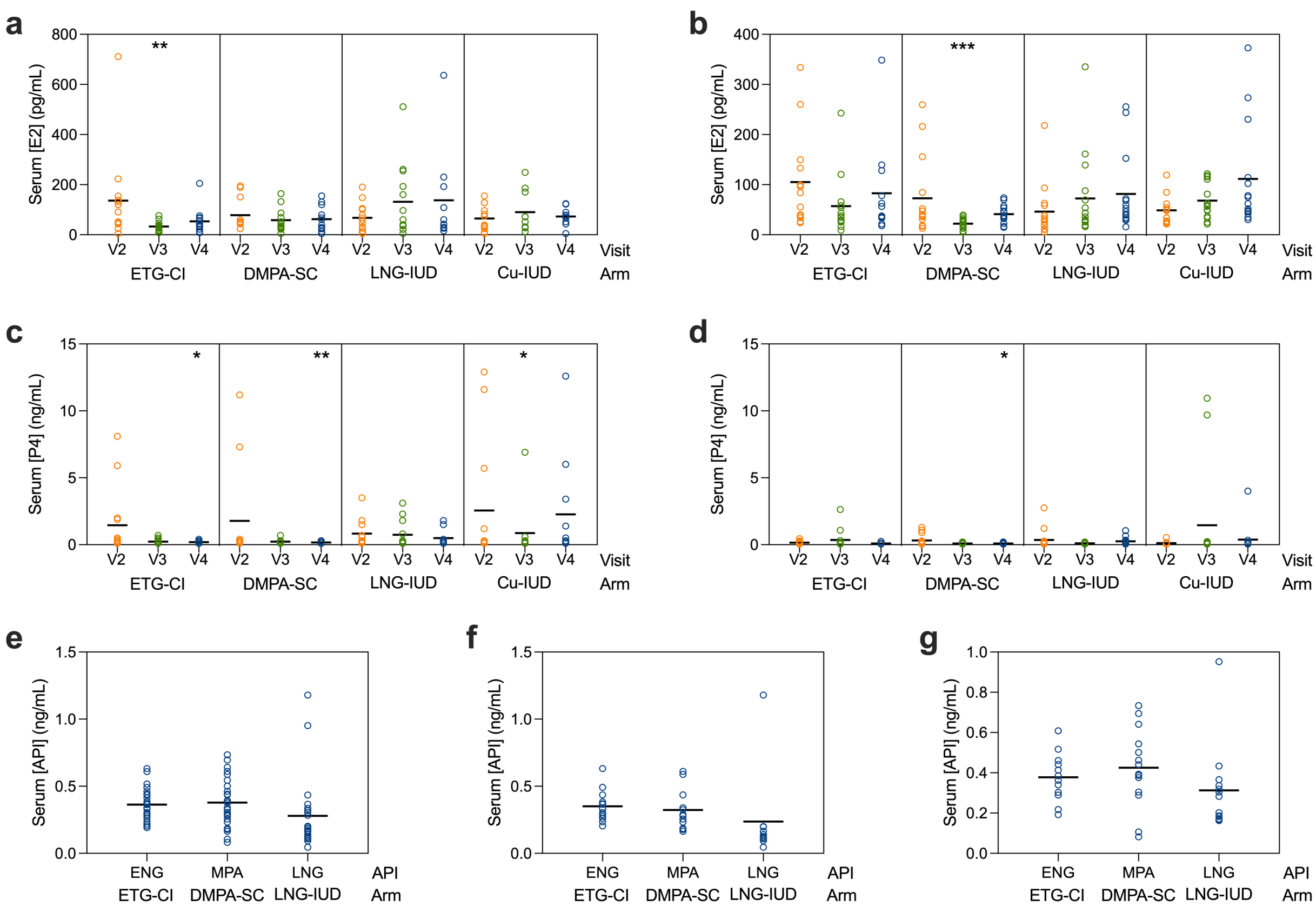

Supplementary Figure S1. Serum levels of estradiol, progesterone and active pharmaceutical ingredient (API). Concentrations of E2, and P4 at each visit in each trial arm are shown for EVMS (**a**, **c**), and KEMRI (**b**, **d**). API levels corresponding to each arm including a hormonal contraceptive and measured at visit 4 (3 months post-contraceptive initiation), are shown for both sites combined (**e**), EVMS (**f**), and KEMRI (**g**). * Indicates statistical difference vs. V2.

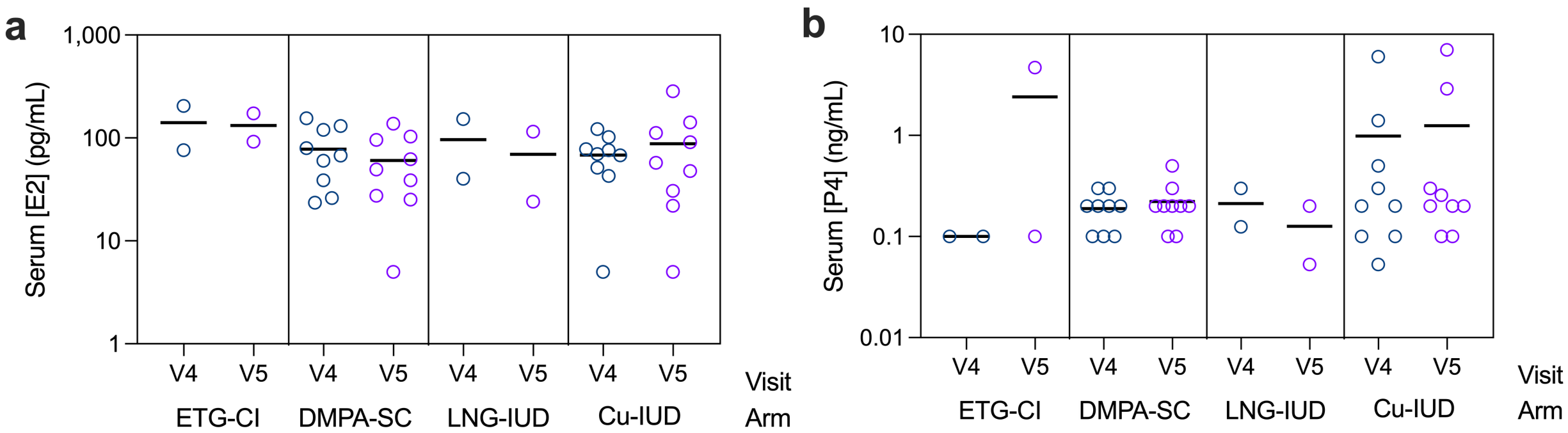

Supplementary Figure S2. Serum levels of estradiol, progesterone after contraceptive discontinuation. Concentrations of E2 (**a**), and P4 (**b**) at V5 are shown for each trial arm.

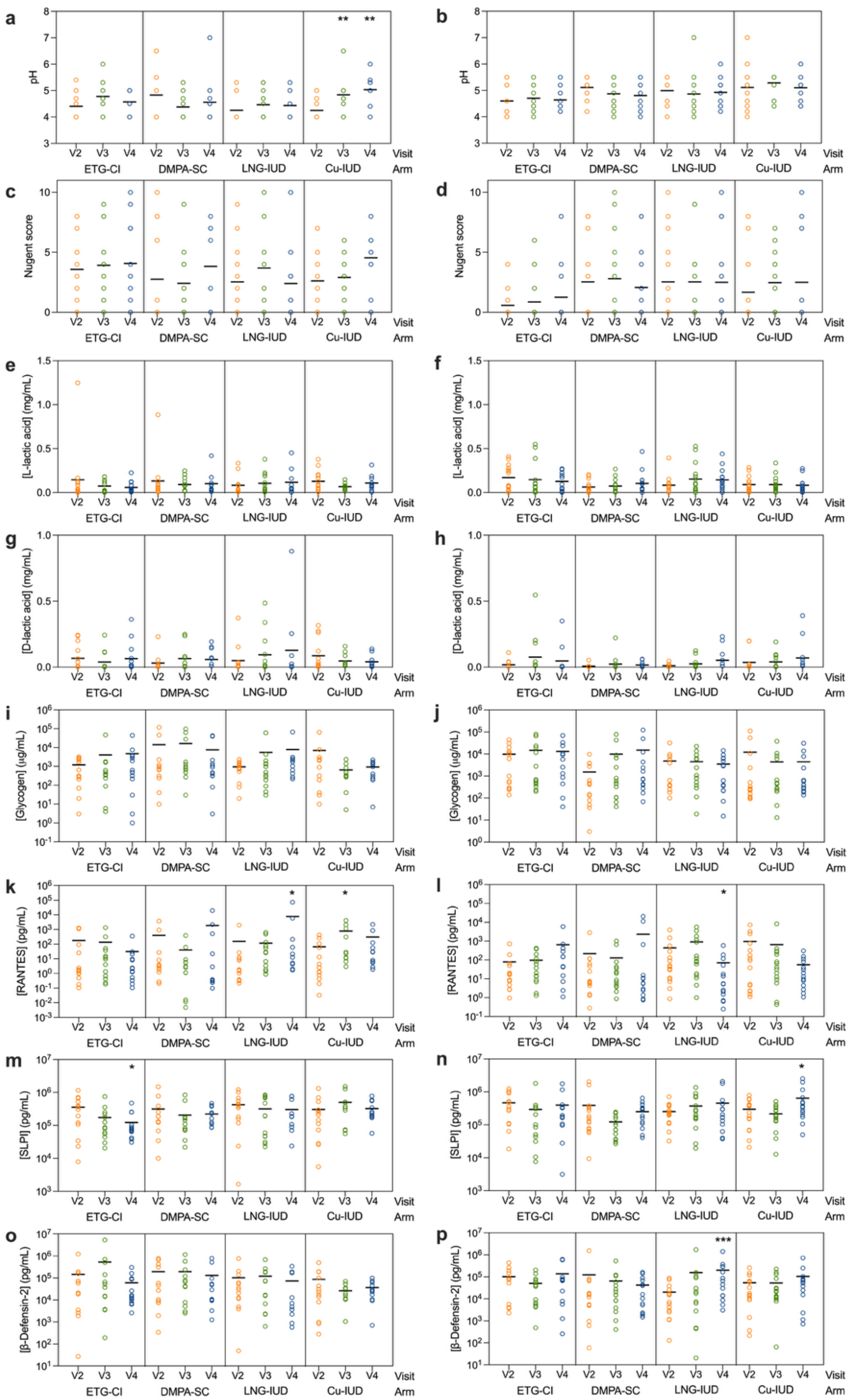

Supplementary Figure S3. Characterization of cervicovaginal environment per trial site. Vaginal pH, Nugent score, and levels of L-lactic acid, D-lactic acid, glycogen, RANTES, SLPI, and β-defensin-2 in cervicovaginal secretions were measured for EVMS (**a**, **c**, **e**, **g**, **i**, **k**, **m**, **o**, respectively) and KEMRI (**b**, **d**, **f**, **h**, **j**, **l**, **n**, **p**, respectively) participants at each visit. * Indicates statistical difference vs. V2.

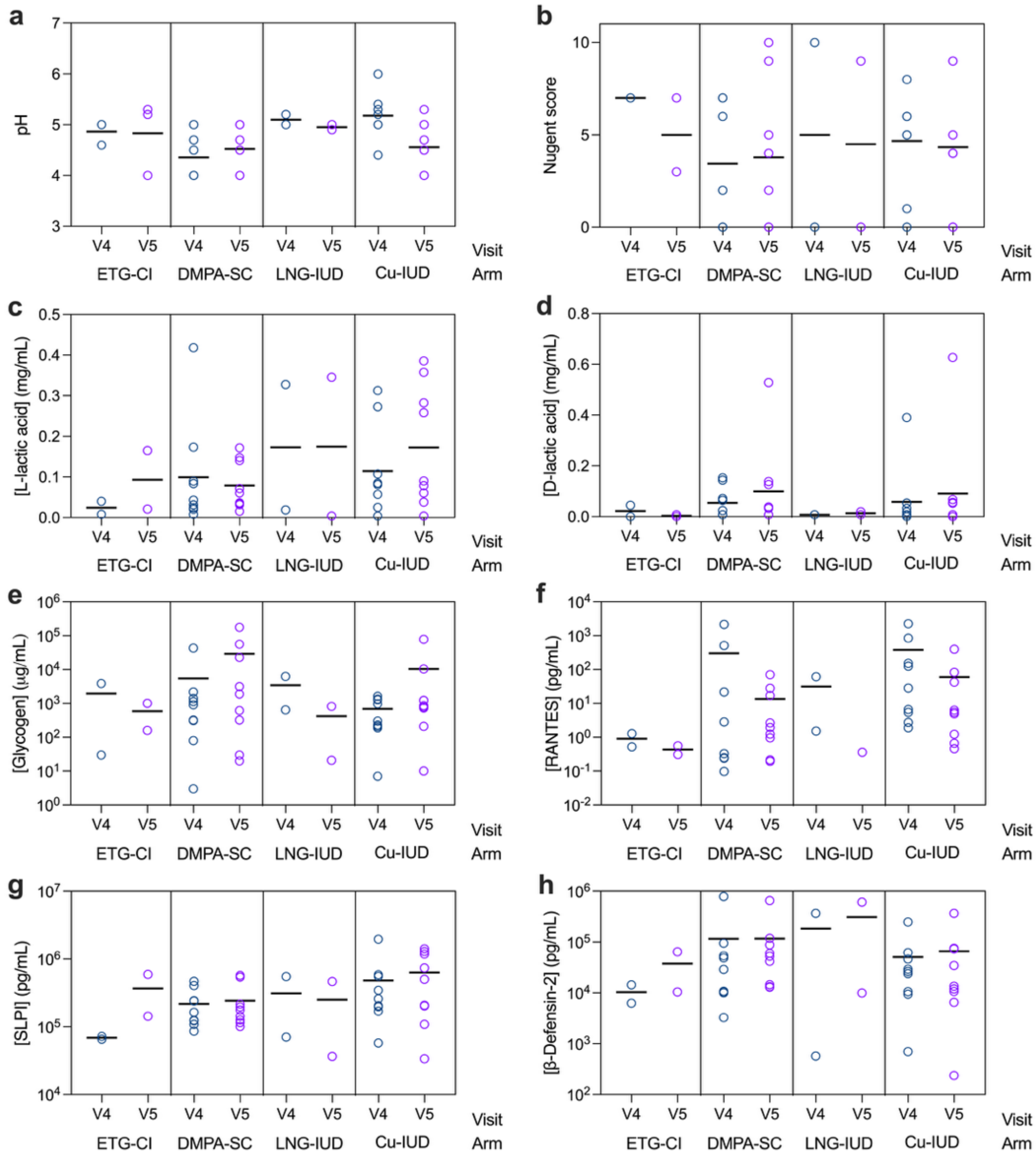

Supplementary Figure S4. Characterization of cervicovaginal environment for participants who discontinued contraceptive use. Vaginal pH (**a**), Nugent score (**b**), and levels of L-lactic acid (**c**), D-lactic acid (**d**), glycogen (**e**), RANTES (**f**), SLPI (**g**), and β-defensin-2 (**h**) in cervicovaginal secretions are shown for participants who attended V4 and V5 per trial arm.

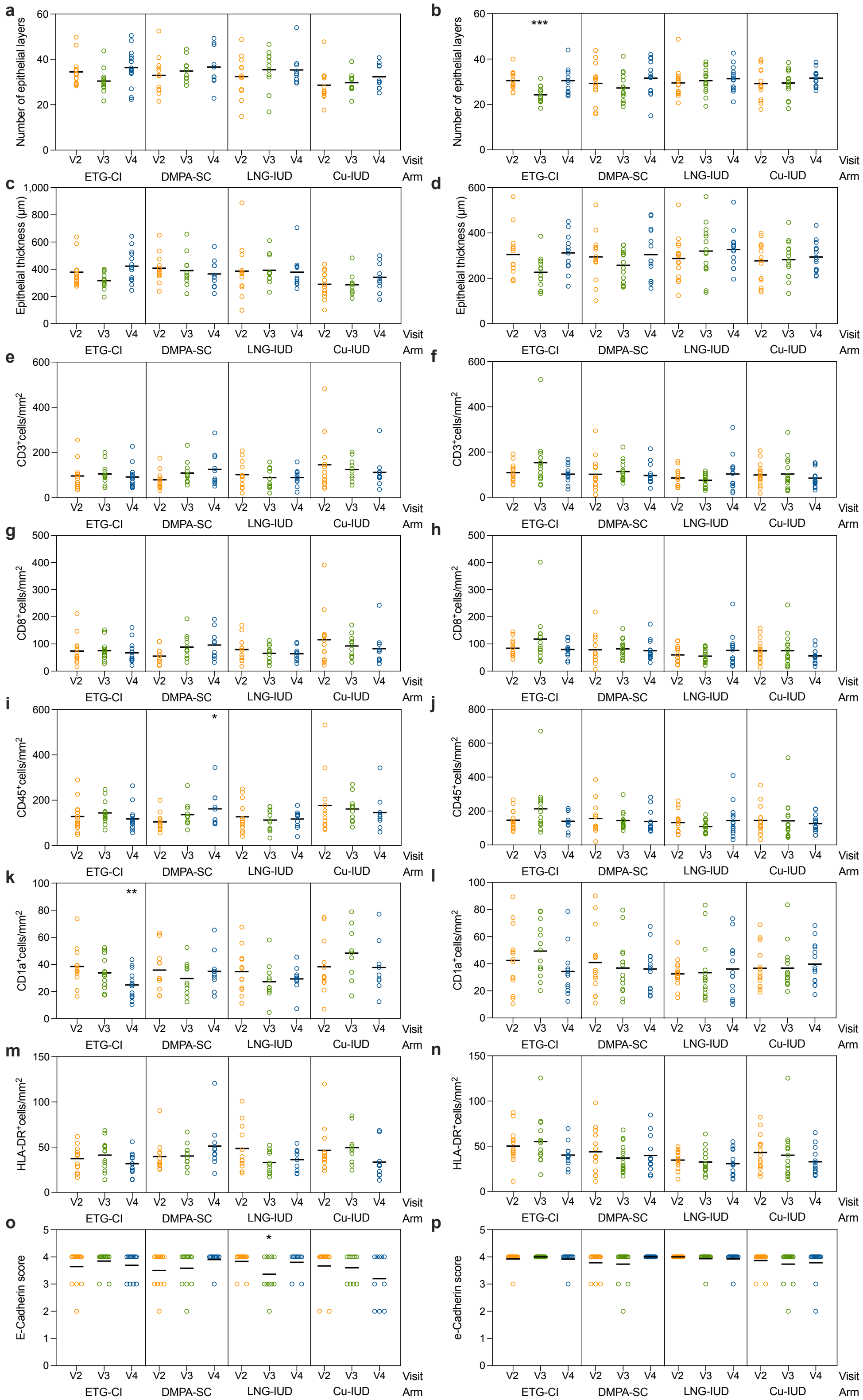

Supplementary Figure S5. Immunohistochemical analysis of vaginal epithelium per trial site. Number of layers and thickness of epithelium, as well as immune cell densities and e-cadherin score were measured for EVMS (**a**, **c**, **e**, **g**, **i**, **k**, **m**, **o**, respectively) and KEMRI (**b**, **d**, **f**, **h**, **j**, **l**, **n**, **p**, respectively) participants at each visit and trial arm. * Indicates statistical difference vs. V2.

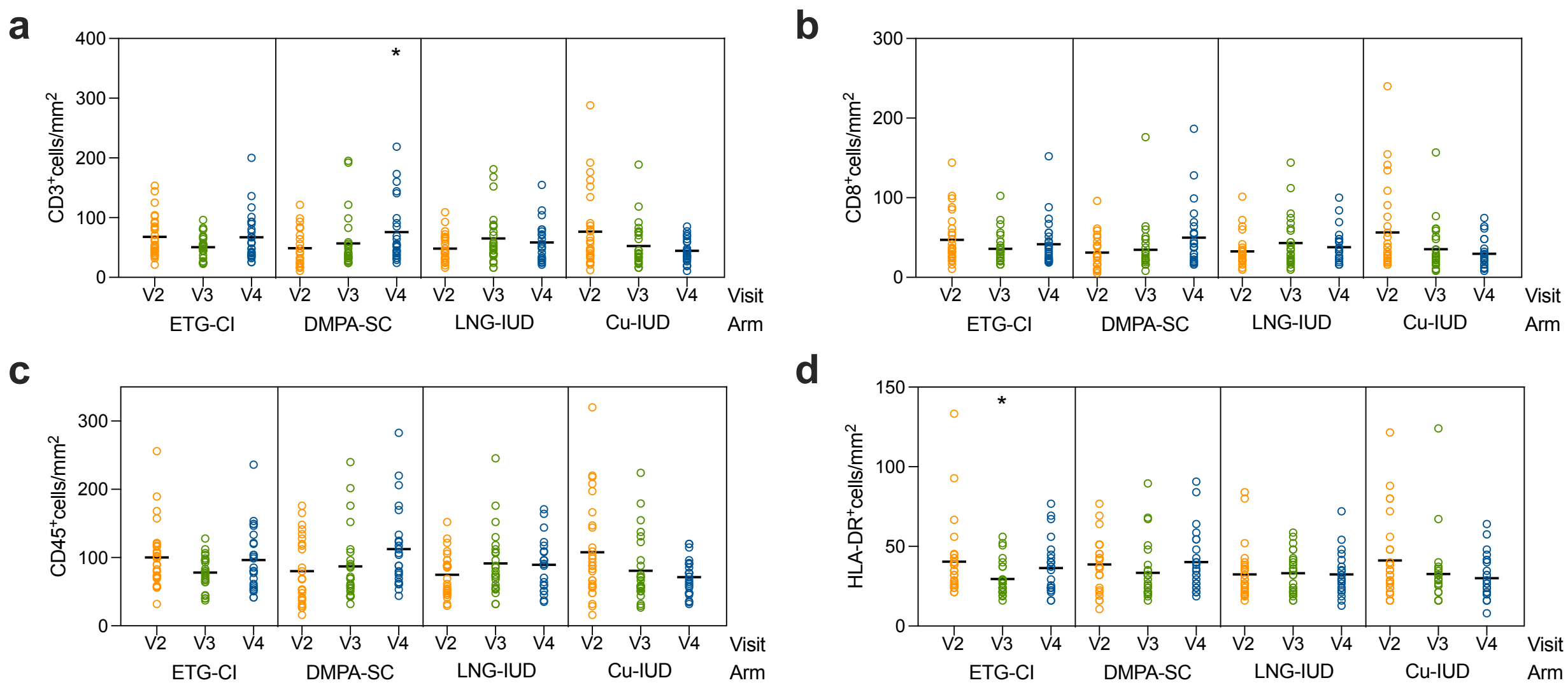

Supplementary Figure S6. Immunohistochemical analysis of vaginal lamina propria. Immune cell densities were measured for participants at each visit per trial arm. * Indicates statistical difference vs. V2.

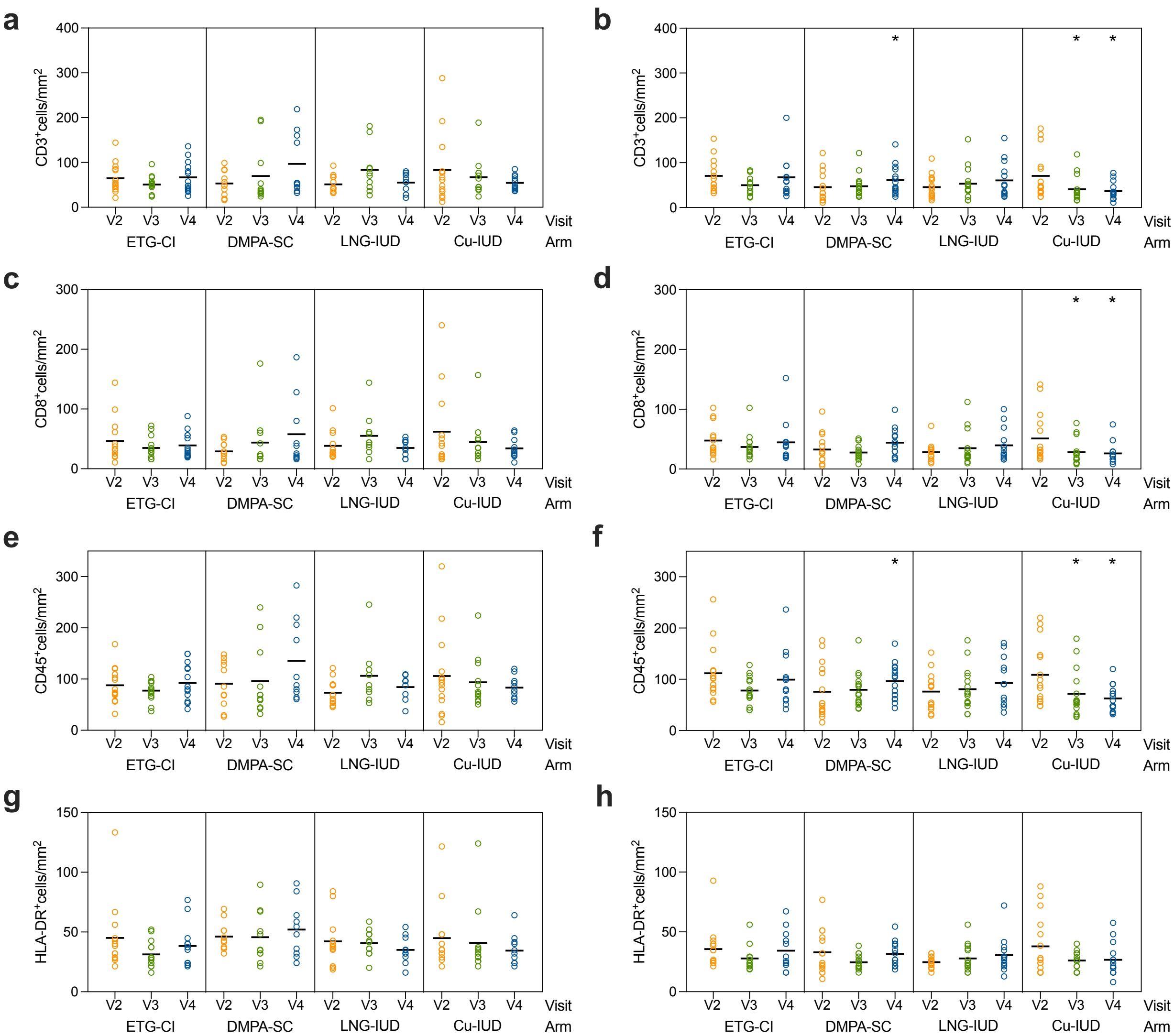

Supplementary Figure S7. Immunohistochemical analysis of vaginal lamina propria per trial site. Immune cell densities were measured for EVMS (**a**, **c**, **e**, **g**) and KEMRI (**b**, **d**, **f**, **h**) participants at each visit and trial arm. * Indicates statistical difference vs. V2.

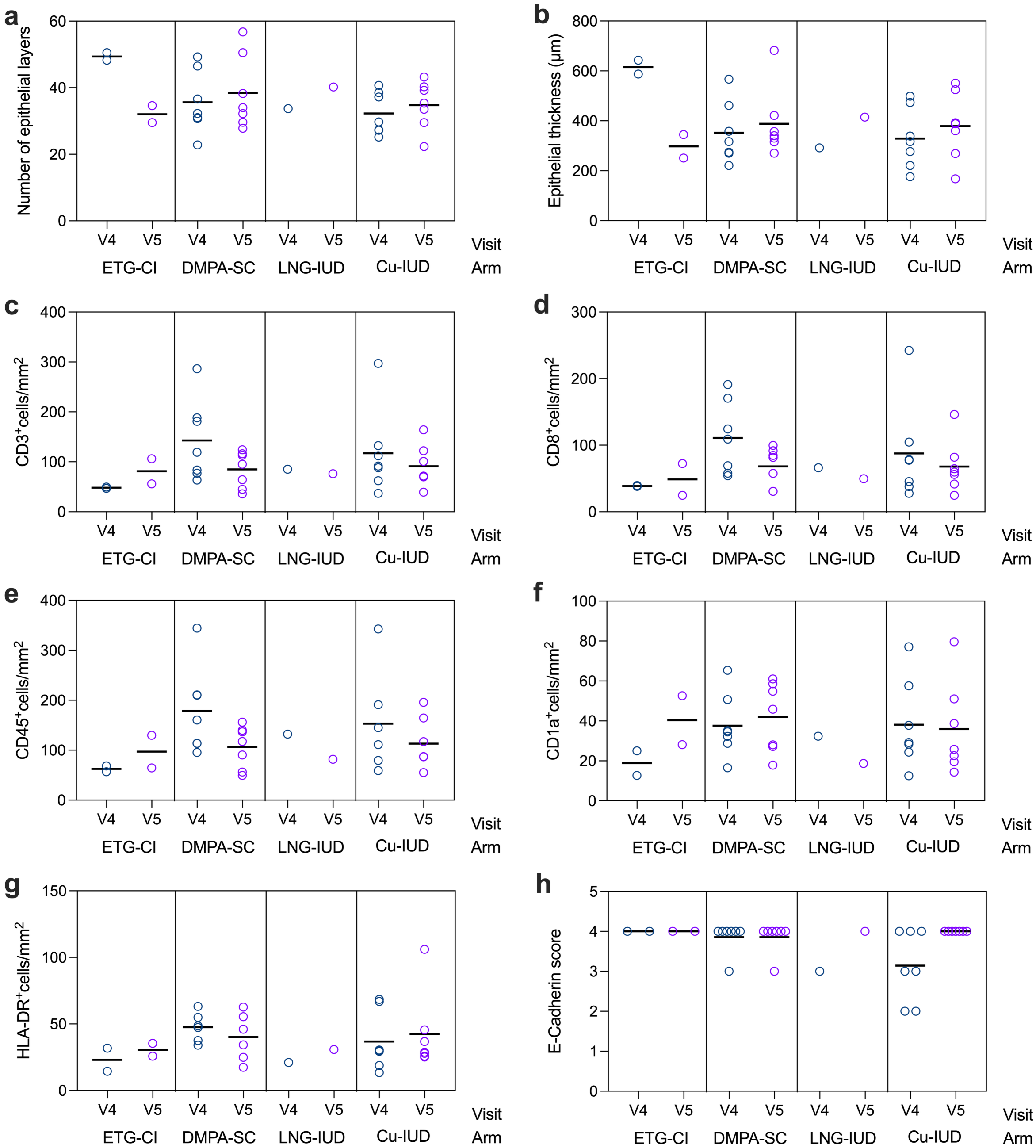

Supplementary Figure S8. Immunohistochemical analysis of vaginal epithelium for participants who discontinued contraceptive use. Number of layers (**a**) and thickness of epithelium (**b**) , as well as immune cell densities (**c**, **d**, **e**, **f**, and **g**) and e-cadherin score (**h**) are shown for participants who attended V4 and V5 per trial arm.

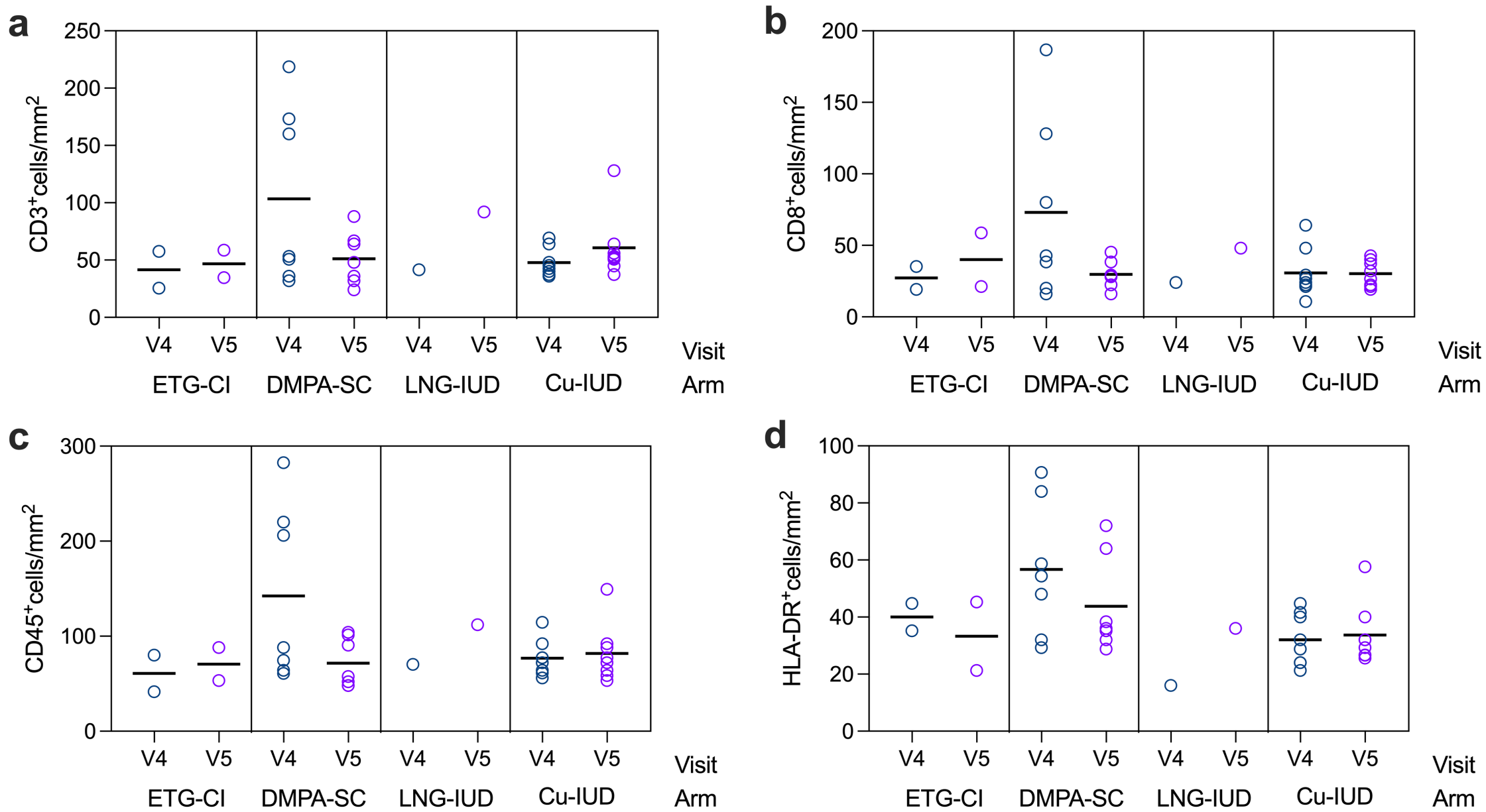

Supplementary Figure S9. Immunohistochemical analysis of vaginal lamina propria for participants who discontinued contraceptive use. Immune cell densities were measured for participants who attended V4 and V5 per trial arm.

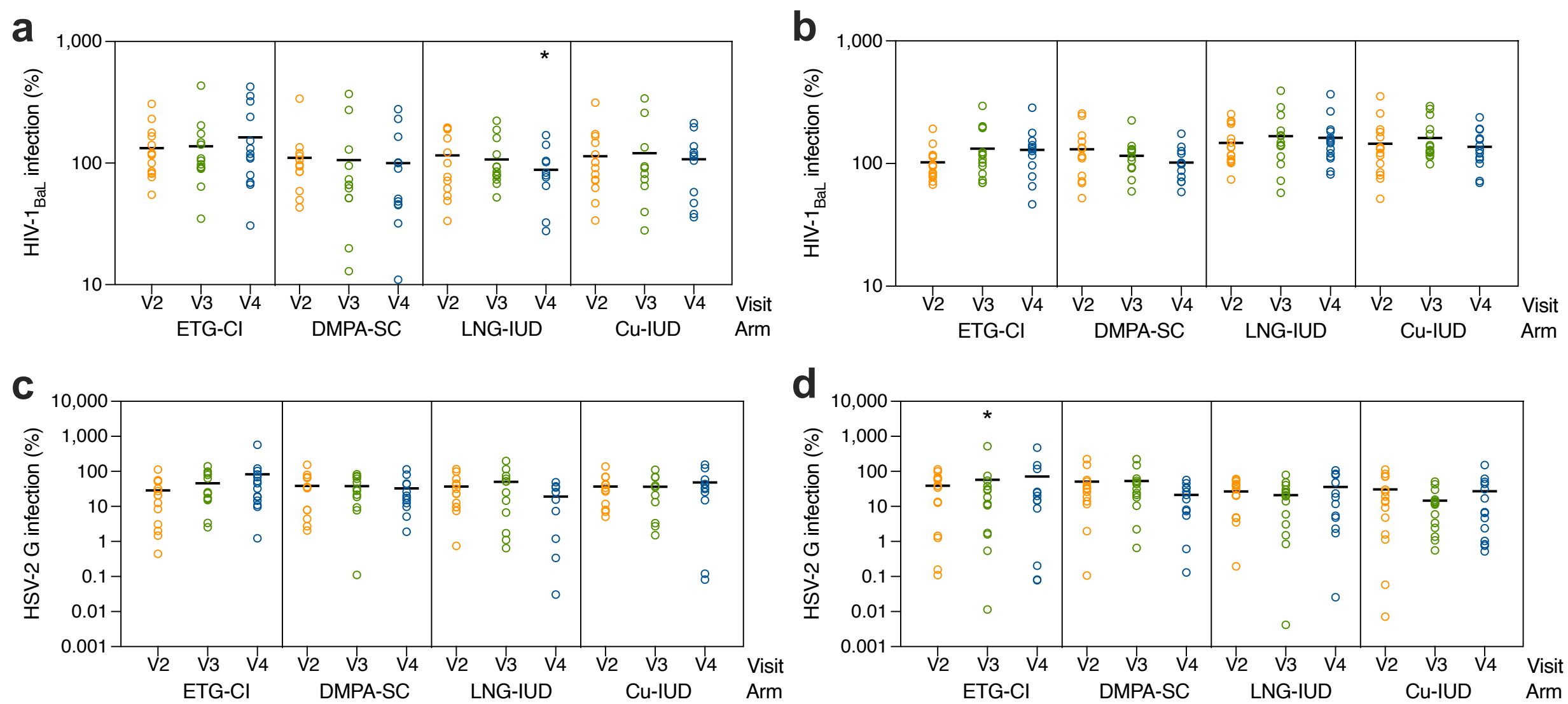

Supplementary Figure S10. Impact of CVFs on *in vitro* HIV-1_{BaL} and HSV-2 strain G infection levels of cellular models per trial site. Infection levels of TZM-bl cells for HIV-1_{BaL} and HEC-1A cells for HSV-2 strain G, in the presence of CVFs from participants at each visit per trial arm from EVMS (**a**, **c**) and KEMRI (**b**, **d**). * Indicates statistical difference vs. V2.

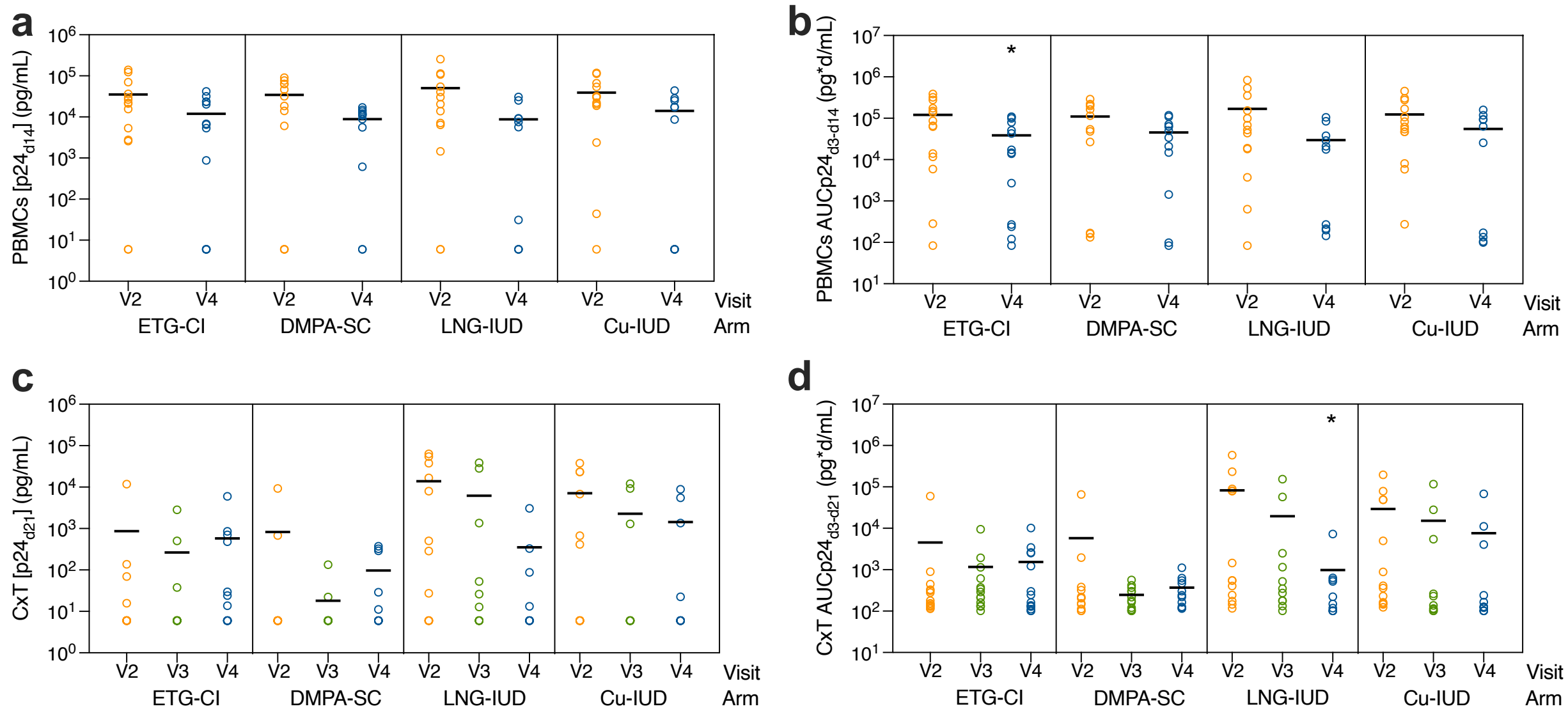

Supplementary Figure S11. *Ex vivo* susceptibility of ecto-cervical biopsies and PBMCs to HIV-1$_{\mathrm{BaL}}$. Infection levels after *ex vivo* exposure of PBMCs (**a**, **b**) and cervical tissue biopsy (**c**, **d**) from EVMS to HIV-1$_{\mathrm{BaL}}$ cells for 14 or 21 days, respectively. Levels of p24 in culture supernatants are shown as concentration at the last day of culture (**a**, **c**) and as area under the curve during the culture period starting at the first harvesting time point (day 3) (**b**, **d**). * Indicates statistical difference vs. V2.

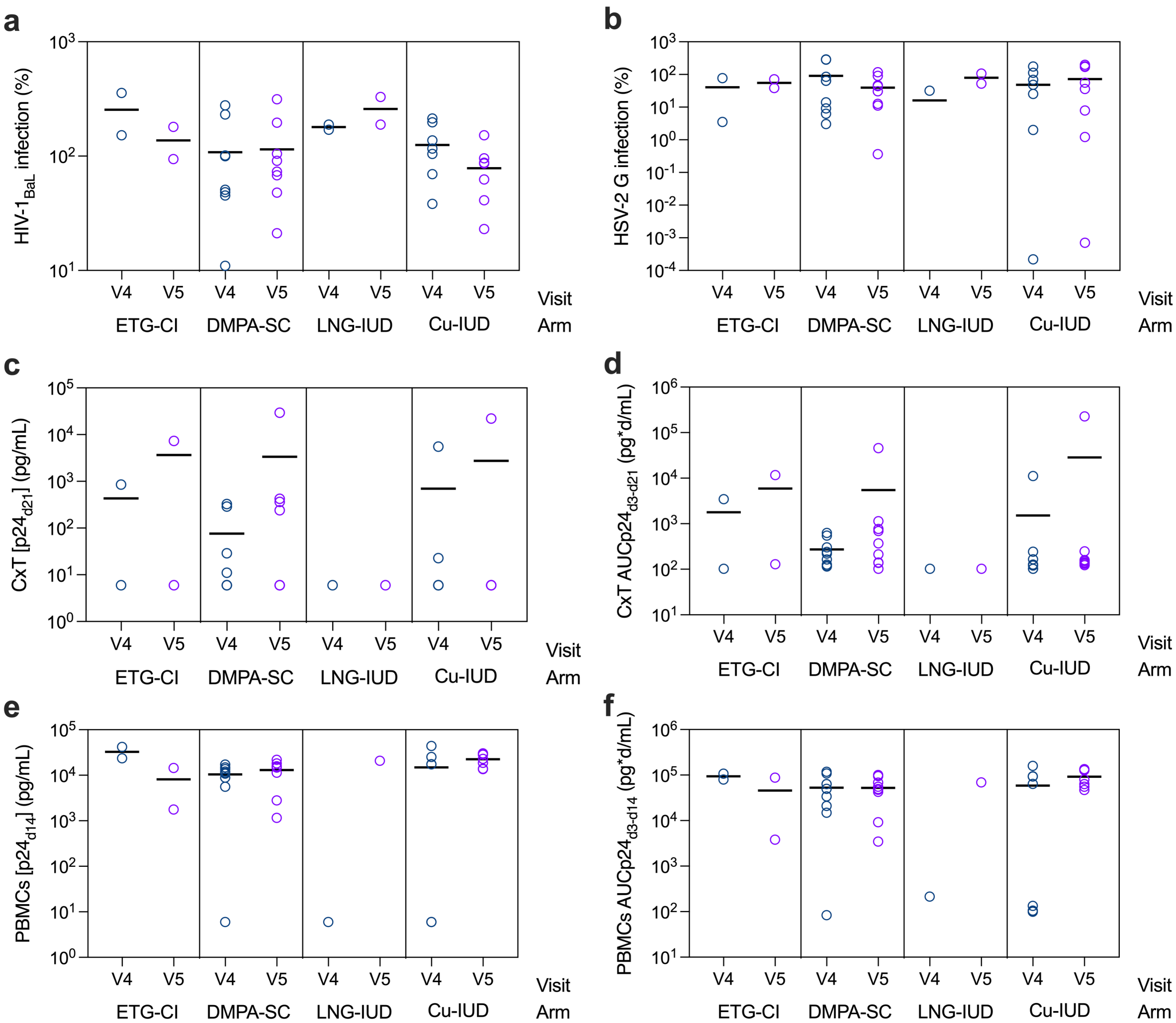

Supplementary Figure 12. *In vitro* and *ex vivo* susceptibility to viral pathogens in participants who discontinued contraceptive use. Infection levels are shown for samples from participants attending V4 and V5 per trial arm. Infection (%) of TZM-bl cells with HIV-1_{BaL} (**a**) and HEC-1A cells with HSV-2 strain G (**b**), in the presence of CVFs. Infection levels after *ex vivo* exposure of cervical tissue biopsy (**c**, **d**) and PBMCs (**e**, **f**) to HIV-1_{BaL} cells for 14 or 21 days, respectively. Levels of p24 in culture supernatants are shown as concentration at the last day of culture (**c**, **e**) and as area under the curve during the culture period starting at the first harvesting time point (day 3) (**d**, **f**).

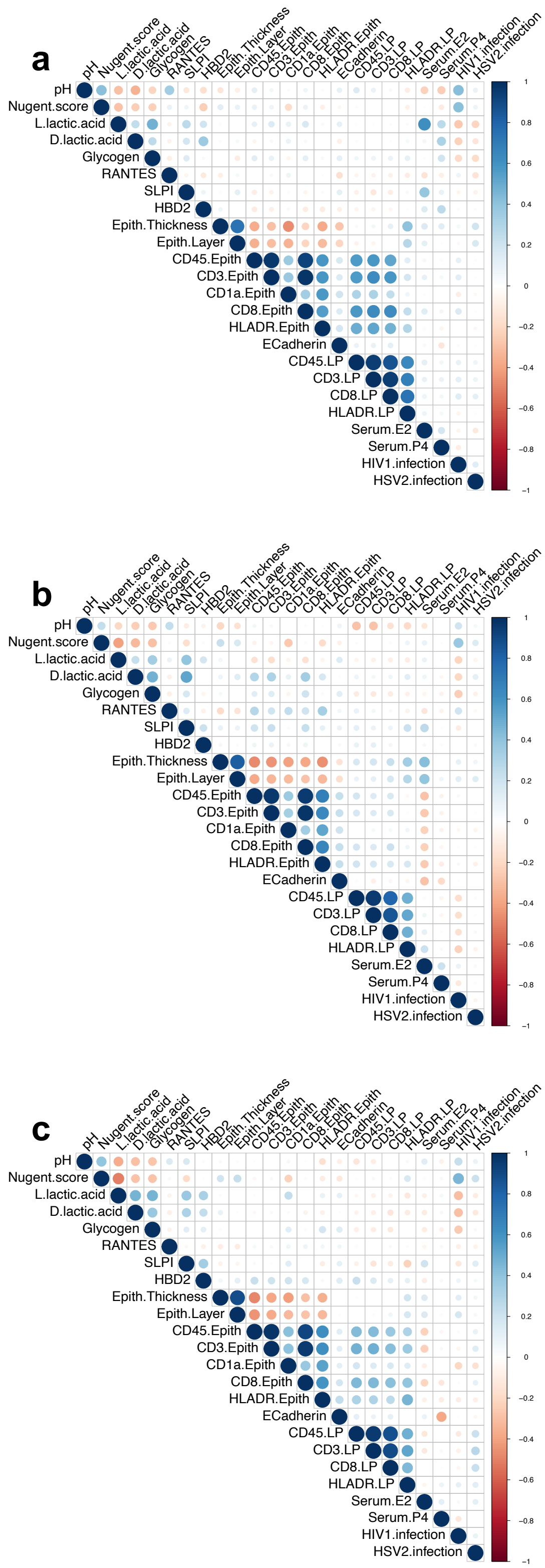

Supplementary Figure S13. Correlogram of UMPALA endpoints. Correlations between endpoints measured at (**a**) V2, (**b**) V3, or (**c**) V4 independently of contraceptive used. Color intensity of positive (blue) and negative (red) correlation coefficients shown in sidebar is proportional to correlation coefficient. Circle size is proportional to statistical significance of correlation.

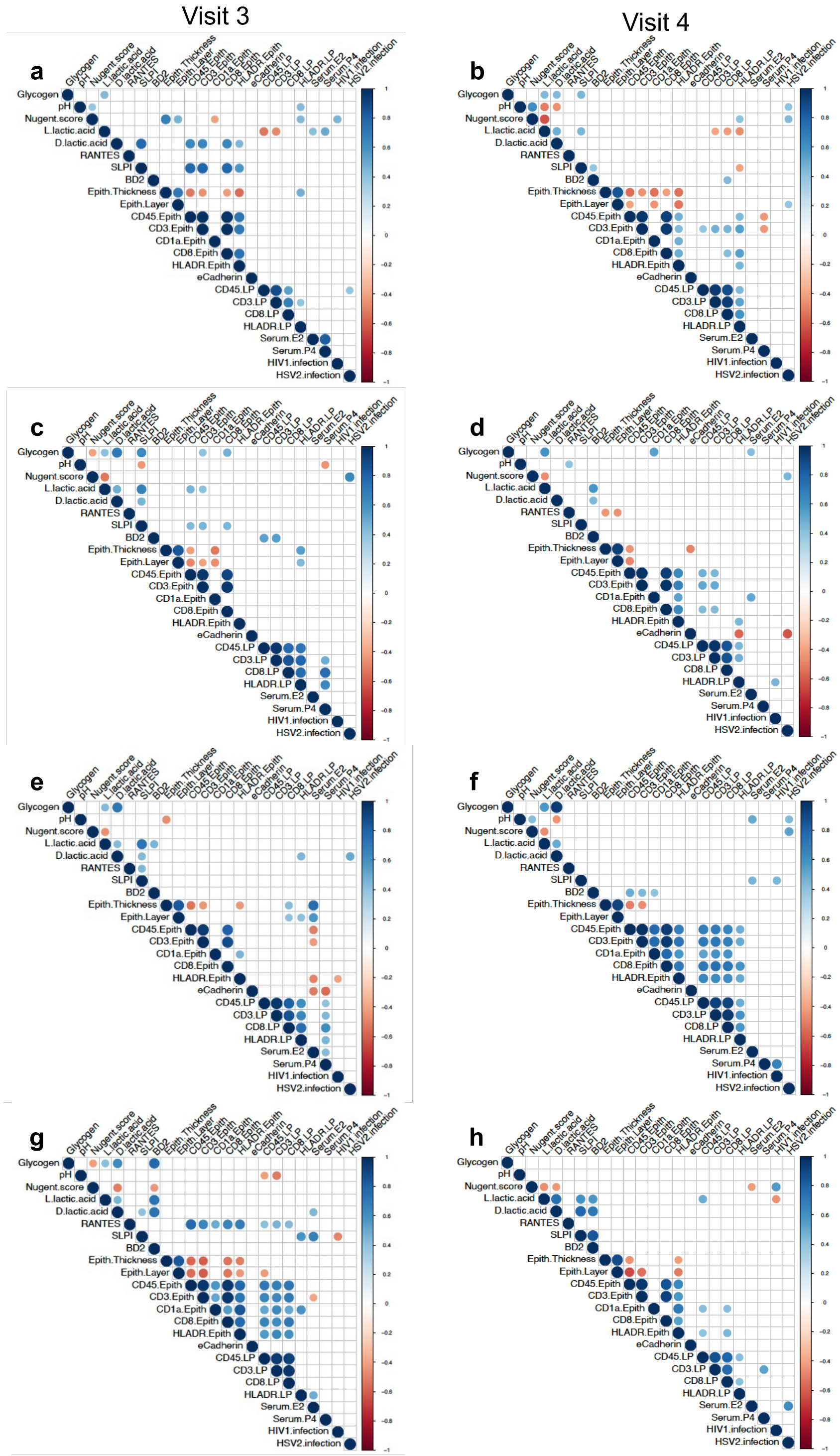

Supplementary Figure S14. Correlogram of UMPALA endpoints per trial arm. Significant ($p < 0.05$) correlations between endpoints measured at (**a**, **c**, **e**, **g**) V3 and (**b**, **d**, **f**, **h**) V4 for participants receiving (**a**, **b**) ETG-CI, (**c**, **d**) DMPA-SC, (**e**, **f**) LNG-IUD, or (**g**, **h**) Cu-IUD independently of clinical site. Color intensity of positive (blue) and negative (red) correlation coefficients shown in sidebar is proportional to correlation coefficient. Circle size is proportional to statistical significance of correlation.

1.2 Supplementary Tables

Supplementary Table S1. Characteristics of UMPALA trial participants who discontinued contraceptive use.

	Visit 5 participants
**Total, N (%[1])**	23 (79.09)
**Participants per site, N (%)**	
EVMS	21 (91.30)
KEMRI	2 (8.70)
**Contraceptive, N (%)**	
ETG-CI	2 (8.70)
DMPA-SC	9 (39.13)
LNG-IUD	2 (8.70)
Cu-IUD	10 (43.48)
**Age, median (IqR)**	40 (28 – 45)
Age (EVMS)	41 (31 – 45)
Age (KEMRI)	24 (20 – 28)
**Ethnicity[2], N (%)**	
Hispanic	2 (9.52)
Non-Hispanic	19 (90.48)
**Race, N (%)**	
African	2 (8.70)
African American	7 (30.43)
White	14 (60.87)
**Marital Status, N (%)**	
Never Married	8 (34.78)
Married	13 (56.52)
Divorced	2 (8.70)
**Education, N (%)**	
High school/GED	4 (17.39)
Some college	8 (34.78)
Associate's degree	4 (17.39)
Bachelor's degree	5 (21.74)
Master's degree	2 (8.70)
**BMI, N (%)**	
Normal Weight	5 (21.74)
Overweight	7 (30.43)
Obesity	11 (47.83)

[1]% calculated vs. the 110 participants enrolled at V2. Rest of %, calculated vs. participants attending V5. [2]Ethnicity of participants at EVMS site.

Supplementary Table S2. Positive PSA assay results.

	Number of participants with positive PSA[1]		
**Visit**	**Total**	**EVMS**	**KEMRI**
V2	11	2	9
V3	16	6	10
V4	7	2	5
V5[2]	0	0	0

[1]PSA results were reported as binary: 0: negative, 1: positive.
[2]For the 23 participants who attended V5.

Supplementary Table S3. Listing of adverse events.

Type of adverse event/severity	Frequency of adverse events									
	ETG-CI		DMPA-SC		LNG-IUD		Cu-IUD		Total	
	Total[1]	Related[2]	Total[1]	Related[2]	Total[1]	Related[2]	Total[1]	Related[2]	Total[1]	Related[2]
Abdominal pain	0	0	1	0	2	1	3	2	6	3
Mild	0	0	1	0	1	1	3	2	5	3
Moderate	0	0	0	0	1	0	0	0	1	0
Menstrual changes	3	3	2	2	5	5	7	7	17	17
Mild	2	2	2	2	4	4	6	6	14	14
Moderate	1	1	0	0	1	1	1	1	3	3
UTI	2	0	3	0	4	0	5	0	14	0
Mild	2	0	3	0	3	0	4	0	12	0
Moderate	0	0	0	0	1	0	1	0	2	0
Vaginal spotting/bleeding	15	14	9	9	10	10	6	2	40	35
Mild	12	11	7	7	8	8	3	0	30	26
Moderate	3	3	2	2	2	2	3	2	10	9
Vaginitis - bacterial/yeast	9	0	9	0	18	1	12	0	48	1
Mild	9	0	9	0	16	1	12	0	46	1
Moderate	0	0	0	0	2	0	0	0	2	0
Other	10	0	7	0	6	1	4	0	27	1
Mild	8	0	7	0	5	0	2	0	22	0
Moderate	2	0	0	0	1	1	2	0	5	0
Total adverse events/contraceptive	39	17	31	11	45	18	37	11	152	57

[1]Total refers all adverse events (related and not related to contraceptive used) reported per group; [2]Related refers to adverse events related to the contraceptive used.

Supplementary Table S4. Serum levels of active pharmaceutical ingredient (API) in hormonal contraceptives arms at visit 4.

	API (trial arm)					
	ETG (ETG-CI)		MPA (DMPA-SC)		LNG (LNG-IUD)	
**Clinical sites**	**Mean (95% CI)**	**p-value***	**Mean (95% CI)**	**p-value***	**Mean (95% CI)**	**p-value***
EVMS and KEMRI	0.36 (0.32, 0.41)		0.38 (0.31, 0.45)		0.28 (0.16, 0.4)	
EVMS	0.35 (0.29, 0.42)	0.465	032 (0.23, 0.42)	< 0.001	0.24 (-0.0021, 0.48)	0.054
KEMRI	0.38 (0.3, 0.45)		0.43 (0.31, 0.54)		0.31 (0.18, 0.44)	

*p-values calculated for each API between clinical sites.

Supplementary Table S5. Levels of oestradiol and progesterone in serum: comparisons between contraceptive trial arms.

Reference visit	Comparator visit	Mean Reference (95% CI)	Mean Comparator (95% CI)	LSM Reference (S.E.)	LSM Comparator (S.E.)	LSMs Difference (S.E.)	p-value
**Outcome = [Oestradiol] (pg/mL)***							
**EVMS and KEMRI**							
ETG-CI V2	ETG-CI V3	120.8 (67.07, 174.5)	45.44 (27.23, 63.65)	77.79 (1.18)	34.12 (1.18)	0.433 (1.25)	<0.001
	ETG-CI V4		67.06 (38, 96.12)		47.22 (1.18)	0.673 (1.25)	0.043
DMPA-SC V2	DMPA-SC V3	75.25 (48.03, 102.5)	38.08 (23.52, 52.64)	54.04 (1.18)	27.13 (1.18)	0.507 (1.24)	0.003
	DMPA-SC V4		51.04 (36.1, 65.99)		39.71 (1.18)	0.725 (1.25)	0.307
LNG-IUD V2	LNG-IUD V3	56.11 (34.94, 77.28)	100.0 (54.28, 145.8)	35.85 (1.18)	57.03 (1.18)	1.578 (1.24)	0.229
	LNG-IUD V4		105.0 (47.78, 162.3)		63.68 (1.19)	1.590 (1.25)	0.105
Cu-IUD V2	Cu-IUD V3	56.44 (41.48, 71.41)	76.91 (53.07, 100.8)	45.28 (1.18)	59.77 (1.19)	1.310 (1.25)	0.335
	Cu-IUD V4		94.63 (60.58, 128.7)		70.57 (1.19)	1.540 (1.25)	0.054
**EVMS**							
ETG-CI V2	ETG-CI V3	136.3 (34.79, 237.9)	32.78 (21.22, 44.35)	78.87 (1.30)	28.02 (1.31)	0.335 (1.25)	0.002
	ETG-CI V4		53.44 (25.73, 81.16)		40.44 (1.30)	0.518 (1.40)	0.057
DMPA-SC V2	DMPA-SC V3	78.09 (38.58, 117.6)	58.09 (27.78, 88.4)	61.09 (1.33)	41.77 (1.33)	0.507 (1.24)	0.460
	DMPA-SC V4		62.39 (30.84, 93.95)		42.62 (1.33)	0.735 (1.41)	0.510
LNG-IUD V2	LNG-IUD V3	67.65 (33.65, 101.6)	131.7 (44.31, 219)	43.44 (1.31)	71.32 (1.31)	1.530 (1.37)	0.440
	LNG-IUD V4		137.9 (2.104, 273.8)		71.82 (1.37)	1.240 (1.44)	0.535
Cu-IUD V2	Cu-IUD V3	65.11 (35.34, 94.87)	90.08 (31.6, 148.6)	47.22 (1.33)	60.36 (1.37)	1.280 (1.43)	0.851
	Cu-IUD V4		73.06 (50.41, 95.71)		59.37 (1.35)	1.17 (1.44)	0.730
**KEMRI**							
ETG-CI V2	ETG-CI V3	105.2 (52.36, 158)	57.19 (22.58, 91.8)	76.73 (1.22)	40.96 (1.22)	0.468 (1.35)	0.085
	ETG-CI V4		82.95 (24.27, 141.6)		56.58 (1.24)	0.769 (1.37)	0.416
DMPA-SC V2	DMPA-SC V3	72.97 (31.01, 114.9)	22.07 (16.27, 27.88)	48.99 (1.21)	19.22 (1.21)	0.424 (1.33)	<0.001
	DMPA-SC V4		41.32 (30.92, 51.71)		37.37 (1.22)	0.769 (1.34)	0.638
LNG-IUD V2	LNG-IUD V3	46.11 (16.8, 75.43)	72.64 (25.57, 119.7)	30.36 (1.21)	46.99 (1.21)	1.640 (1.33)	0.394
	LNG-IUD V4		81.54 (36.31, 126.8)		58.44 (1.22)	2.060 (1.34)	0.070
Cu-IUD V2	Cu-IUD V3	48.94 (34.69, 63.18)	68.13 (48.82, 87.45)	43.79 (1.21)	59.38 (1.21)	1.340 (1.32)	0.170
	Cu-IUD V4		111.6 (51.06, 172.1)		80.84 (1.22)	1.910 (1.33)	0.059
**Outcome = [Progesterone] (ng/mL)§**							

**EVMS and KEMRI**							
ETG-CI V2	ETG-CI V3	0.81 (0.097, 1.5)	0.30 (0.093, 0.5)	0.218 (0.1, 0.35)	0.103 (0.1, 0.3)	-0.012 (-0.2, 0.057)	0.226
	ETG-CI V4		0.16 (0.11, 0.2)		0.100 (0.086, 0.228)	-0.010 (-0.216, 0)	0.006
DMPA-SC V2	DMPA-SC V3	0.97 (-0.0084, 1.9)	0.16 (0.1, 0.21)	0.200 (0.1, 0.4)	0.1 (0.071, 0.2)	-0.1 (-0.2, 0.002)	0.008
	DMPA-SC V4		0.13 (0.095, 0.16)		0.1 (0.064, 0.2)	-0.12 (-0.2, 0.03)	<0.001
LNG-IUD V2	LNG-IUD V3	0.58 (0.23, 0.92)	0.40 (0.11, 0.69)	0.153 (0.076, 0.65)	0.120 (0.079, 0.201)	0 (-0.2, 0.1)	0.560
	LNG-IUD V4		0.35 (0.16, 0.54)		0.2 (0.1, 0.346)	0 (-0.134, 0.086)	0.524
Cu-IUD V2	Cu-IUD V3	1.3 (-0.025, 2.5)	1.2 (-0.04, 2.5)	0.13 (0.086, 0.3)	0.1 (0.059, 0.2)	-0.018 (-0.1, 0.001)	0.193
	Cu-IUD V4		1.2 (0.05, 2.4)		0.103 (0.053, 0.334)	-0.014 (-0.101, 0.1)	0.633
**EVMS**							
ETG-CI V2	ETG-CI V3	1.5 (0.033, 2.9)	0.23 (0.11, 0.35)	0.30 (0.20, 1.90)	0.10 (0.10, 0.30)	-0.2 (-1.7, 0)	0.076
	ETG-CI V4		0.20 (0.14, 0.26)		0.20 (0.10, 0.30)	-0.1 (-1.8, 0)	0.029
DMPA-SC V2	DMPA-SC V3	1.8 (-0.51, 4.1)	0.23 (0.12, 0.34)	0.30 (0.20, 0.40)	0.20 (0.10, 0.30)	-0.1 (-0.2, -0.05)	0.113
	DMPA-SC V4		0.17 (0.12, 0.22)		0.15 (0.10, 0.20)	-0.2 (-0.2, -0.1)	0.007
LNG-IUD V2	LNG-IUD V3	0.83 (0.22, 1.4)	0.74 (0.13, 1.3)	0.30 (0.10, 1.5)	0.20 (0.10, 0.80)	0 (-0.5, 0.1)	0.719
	LNG-IUD V4		0.49 (0.045, 0.94)		0.20 (0.10, 0.40)	-0.1 (-1.3, 0.1)	0.258
Cu-IUD V2	Cu-IUD V3	2.6 (-0.21, 5.3)	0.87 (-0.65, 2.4)	0.25 (0.20, 3.45)	0.15 (0.10, 0.30)	-0.1 (-4.7, 0)	0.031
	Cu-IUD V4		2.3 (-0.36, 4.9)		0.30 (0.10, 3.40)	-0.1 (-5.6, 1.3)	0.668
**KEMRI**							
ETG-CI V2	ETG-CI V3	0.16 (0.091, 0.23)	0.36 (-0.046, 0.77)	0.12 (0.06, 0.26)	0.10 (0.09, 0.16)	0.002 (-0.08, 0.057)	0.999
	ETG-CI V4		0.10 (0.052, 0.15)		0.08 (0.05, 0.12)	-0.008 (-0.089, 0.021)	0.189
DMPA-SC V2	DMPA-SC V3	0.32 (0.094, 0.55)	0.095 (0.065, 0.13)	0.15 (0.08, 0.28)	0.07 (0.05, 0.15)	-0.062 (-0.15, 0.024)	0.073
	DMPA-SC V4		0.090 (0.056, 0.12)		0.06 (0.05, 0.10)	-0.105 (-0.23, -0.023)	0.033
LNG-IUD V2	LNG-IUD V3	0.36 (-0.046, 0.76)	0.11 (0.073, 0.14)	0.08 (0.05, 0.24)	0.09 (0.05, 0.16)	0 (-0.101, 0.072)	0.677
	LNG-IUD V4		0.25 (0.085, 0.41)		0.15 (0.08, 0.29)	0.015 (-0.058, 0.107)	0.691
Cu-IUD V2	Cu-IUD V3	0.12 (0.053, 0.19)	1.5 (-0.54, 3.5)	0.09 (0.05, 0.13)	0.09 (0.05, 0.17)	0 (-0.058, 0.085)	0.783
	Cu-IUD V4		0.38 (-0.23, 0.98)		0.06 (0.05, 0.15)	-0.007 (-0.037, 0.046)	0.927

LSM: Least Squares Mean. *LSM were calculated using log_{10}mean values for each analyte.
[§]Median and Interquartile Range were reported instead of LSM and S.E.

Supplementary Table S6. Characteristics of cervicovaginal secretions: comparison between trial arms.

Reference visit	Comparator visit	Mean Reference (95% CI)	Mean Comparator (95% CI)	LSM Reference (S.E.)	LSM Comparator (S.E.)	LSMs Difference (S.E.)	p-value
**Outcome = pH[§]**							
**EVMS and KEMRI**							
ETG-CI V2	ETG-CI V3	4.50 (4.3, 4.7)	4.74 (4.55, 4.93)	4.5 (4, 4.8)	4.7 (4.4, 5)	0 (-0.2, 0.6)	0.172
	ETG-CI V4		4.60 (4.43, 4.78)		4.6 (4.2, 5)	0 (-0.3, 0.5)	0.556
DMPA-SC V2	DMPA-SC V3	4.99 (4.67, 5.3)	4.65 (4.43, 4.88)	5.2 (4, 5.5)	4.6 (4, 5.2)	0 (-0.7, 0)	0.036
	DMPA-SC V4		4.69 (4.4, 4.98)		4.5 (4, 5.2)	0 (-1, 0)	0.088
LNG-IUD V2	LNG-IUD V3	4.65 (4.42, 4.88)	4.68(4.42, 4.94)	4.6 (4, 5.2)	4.6 (4, 5.1)	0 (-.45, 0.5)	0.977
	LNG-IUD V4		4.72 (4.49, 4.95)		4.6 (4.4, 5.2)	0 (-0.3, 0.5)	0.996
Cu-IUD V2	Cu-IUD V3	4.71 (4.42, 5)	5.11 (4.87, 5.34)	4.6 (4, 5.2)	5.2 (4.7, 5.5)	0.3 (0, 0.9)	0.004
	Cu-IUD V4		5.07 (4.86, 5.28)		5.2 (4.9, 5.3)	0.3 (0, 1)	0.044
**EVMS**							
ETG-CI V2	ETG-CI V3	4.40 (4.12, 4.68)	4.78 (4.46, 5.09)	4.2 (4, 4.7)	4.7 (4.5, 5)	0 (0, 0.5)	0.086
	ETG-CI V4		4.57 (4.3, 4.85)		4.6 (4, 5)	0 (-0.7, 0.5)	0.328
DMPA-SC V2	DMPA-SC V3	4.83 (4.13, 5.54)	4.38 (4.06, 4.69)	4 (4, 6)	4 (4, 4.8)	0 (-0.8, 0)	0.125
	DMPA-SC V4		4.56 (4.01, 5.11)		4.2 (4, 4.8)	0 (-0.6, 0)	0.375
LNG-IUD V2	LNG-IUD V3	4.25 (3.96, 4.55)	4.46 (4.15, 4.77)	4 (4, 4)	4.5 (4, 4.7)	0 (0, 0.5)	0.094
	LNG-IUD V4		4.43 (4.07, 4.79)		4.5 (4, 5)	0 (0, 0.5)	0.312
Cu-IUD V2	Cu-IUD V3	4.25 (4, 4.49)	4.84 (4.34, 5.34)	4 (4, 4.6)	4.8 (4.5, 5)	0.6 (0.2, 1)	0.008
	Cu-IUD V4		4.69 (4.69, 5.38)		5 (5, 5.3)	0.5 (0, 1.3)	0.008
**KEMRI**							
ETG-CI V2	ETG-CI V3	4.6 (4.28, 4.92)	4.71 (4.45, 4.96)	4.6 (4.2, 5.2)	4.6 (4.4, 5.2)	-0.1 (-0.3, 0.6)	0.680
	ETG-CI V4		4.64 (4.38, 4.9)		4.6 (4.4, 4.9)	-0.2 (-0.3, 0.3)	0.739
DMPA-SC V2	DMPA-SC V3	5.11 (4.89, 5.33)	4.87 (4.57, 5.17)	5.2 (4.9, 5.5)	4.9 (4.4, 5.5)	0 (-0.7, 0)	0.148
	DMPA-SC V4		4.81 (4.48, 5.13)		4.9 (4.2, 5.2)	-0.1 (-1, 0)	0.156
LNG-IUD V2	LNG-IUD V3	4.99 (4.73, 5.25)	4.87 (4.44, 5.29)	5.2 (4.6, 5.2)	4.6 (4.4, 5.2)	-0.3 (-0.8, 0.9)	0.609
	LNG-IUD V4		4.93 (4.64, 5.22)		4.9 (4.4, 5.2)	-0.15 (-0.6, 0.6)	0.541
Cu-IUD V2	Cu-IUD V3	5.11 (4.7, 5.53)	5.29 (5.07, 5.51)	5.2 (4.6, 5.5)	5.5 (5.2, 5.5)	0.3 (0, 0.6)	0.201
	Cu-IUD V4		5.10 (4.8, 5.4)		5.2 (4.6, 5.2)	0 (-0.3, 0.3)	0.951
**Outcome = Nugent score[§]**							

**EVMS and KEMRI**							
ETG-CI V2	ETG-CI V3	2.07 (1.05, 3.09)	2.33 (1.19, 3.47)	1 (0, 4)	1 (0, 4)	0 (-1, 2)	0.439
	ETG-CI V4		2.77 (1.38, 4.16)		1 (0, 7)	0 (0, 2)	0.283
DMPA-SC V2	DMPA-SC V3	2.63 (1.19, 4.07)	2.63 (1.4, 3.86)	0 (0, 7)	1 (0, 4)	0 (-2, 2)	0.872
	DMPA-SC V4		2.88 (1.58, 4.19)		1.5 (0, 7)	0 (-1, 0)	0.808
LNG-IUD V2	LNG-IUD V3	2.54 (1.32, 3.76)	3.07 (1.75, 4.39)	1 (0, 4.5)	2 (0, 4.5)	0 (-2, 2)	0.923
	LNG-IUD V4		2.46 (1.05, 3.87)		1 (0 -4)	0 (-1, 3)	0.908
Cu-IUD V2	Cu-IUD V3	2.11 (1.05, 3.16)	2.64 (1.74, 3.54)	0 (0, 4)	3 (0, 4)	0 (0, 2)	0.120
	Cu-IUD V4		3.40 (2.05, 4.75)		4 (0, 6)	0 (0, 4)	0.175
**EVMS**							
ETG-CI V2	ETG-CI V3	3.57 (1.91, 5.23)	3.92 (2.12, 5.73)	3.5 (1, 5)	4 (1, 5)	1 (-1, 2)	0.599
	ETG-CI V4		4.07 (1.95, 6.19)		3.5 (0 -7)	0.5 (-1, 2)	0.462
DMPA-SC V2	DMPA-SC V3	2.75 (0.221, 5.28)	2.42 (0.715, 4.12)	0 (0, 7)	1.5 (0.5, 4)	0 (-2.5, 1)	0.812
	DMPA-SC V4		3.83 (1.67, 6)		4 (0, 7)	0 (-0.5, 2)	0.484
LNG-IUD V2	LNG-IUD V3	2.54 (0.766, 4.31)	3.69 (1.47, 5.92)	1 (0, 4)	2 (1, 5)	0 (-2, 3)	0.613
	LNG-IUD V4		2.40 (0.158, 4.64)		1 (0, 4)	0 (-1, 3)	0.906
Cu-IUD V2	Cu-IUD V3	2.62 (0.969, 4.26)	2.90 (1.62, 4.18)	1.5 (0, 5)	3 (2, 4)	1 (-2, 2)	0.609
	Cu-IUD V4		4.55 (3, 6.09)		5 (4, 6)	1 (-1, 4)	0.129
**KEMRI**							
ETG-CI V2	ETG-CI V3	0.571 (-0.097, 1.24)	0.857 (-0.226, 1.94)	0 (0, 1)	0 (0, 0)	0 (0, 0)	0.781
	ETG-CI V4		1.25 (-0.356, 2.86)		0 (0, 3)	0 (0, 0)	0.500
DMPA-SC V2	DMPA-SC V3	2.53 (0.605, 4.46)	2.80 (0.857, 4.74)	0 (0, 7)	1 (0, 5)	0 (-1, 2)	0.656
	DMPA-SC V4		2.07 (0.355, 3.79)		0.5 (0, 4)	0 (-3, 0)	0.422
LNG-IUD V2	LNG-IUD V3	2.53 (0.639, 4.43)	2.53 (0.784, 4.28)	1 (0, 5)	1 (0, 4)	0 (-2, 1)	0.687
	LNG-IUD V4		2.50 (0.427, 4.57)		0.5 (0, 4)	0 (-5, 3)	0.860
Cu-IUD V2	Cu-IUD V3	1.67 (0.148, 3.19)	2.47 (1.11, 3.82)	0 (0, 4)	2 (0, 4)	0 (0, 3)	0.180
	Cu-IUD V4		2.50 (0.367, 4.63)		0.5 (0 -7)	0 (-4, 1)	0.547
**Outcome = [L-Lactic acid] (mg/mL) §**							
**EVMS and KEMRI**							
ETG-CI V2	ETG-CI V3	0.158 (0.063, 0.253)	0.111 (0.053, 0.169)	0.074 (0.024, 0.234)	0.060 (0.014, 0.142)	0 (-0.141, 0.075)	0.831
	ETG-CI V4		0.089 (0.053, 0.125)		0.040 (0.015, 0.170)	-0.001 (-0.082, 0.015)	0.290
DMPA-SC V2	DMPA-SC V3	0.093 (0.026, 0.16)	0.081 (0.050, 0.111)	0.041 (0.017, 0.097)	0.053 (0.019, 0.124)	0.002 (-0.026, 0.054)	0.887
	DMPA-SC V4		0.102 (0.054, 0.15)		0.045 (0.032, 0.140)	0.011 (-0.041, 0.086)	0.680

LNG-IUD V2	LNG-IUD V3	0.082 (0.044, 0.12)	0.131 (0.074, 0.187)	0.054 (0.025, 0.094)	0.071 (0.024, 0.197)	0.024 (-0.028, 0.098)	0.068
	LNG-IUD V4		0.133 (0.078, 0.187)			0.029 (-0.002, 0.119)	0.028
Cu-IUD V2	Cu-IUD V3	0.106 (0.066, 0.147)	0.081 (0.049, 0.112)	0.075 (0.019, 0.186)	0.067 (0.039, 0.094)	-0.012 (-0.081, 0.020)	0.220
	Cu-IUD V4		0.093 (0.059, 0.127)		0.074 (0.040, 0.104)	0 (-0.071, 0.033)	0.701
**EVMS**							
ETG-CI V2	ETG-CI V3	0.145 (-0.041, 0.332)	0.073 (0.029, 0.116)	0.037 (0.010, 0.123)	0.025 (0.013, 0.141)	-0.005 (-0.015, 0.019)	0.813
	ETG-CI V4		0.058 (0.021, 0.094)		0.029 (0.013, 0.083)	-0.001 (-0.018, 0.021)	0.855
DMPA-SC V2	DMPA-SC V3	0.131 (-0.023, 0.285)	0.091 (0.039, 0.142)	0.055 (0.022, 0.109)	0.077 (0.020, 0.146)	-0.004 (-0.042, 0.021)	0.733
	DMPA-SC V4		0.101 (0.028, 0.174)		0.063 (0.028, 0.141)	0.009 (-0.049, 0.057)	0.970
LNG-IUD V2	LNG-IUD V3	0.081 (0.019, 0.144)	0.104 (0.035, 0.173)	0.032 (0.027, 0.090)	0.051 (0.014, 0.188)	0.023 (-0.024, 0.081)	0.414
	LNG-IUD V4		0.115 (0.014, 0.217)		0.049 (0.036, 0.258)	0.029 (-0.014, 0.118)	0.102
Cu-IUD V2	Cu-IUD V3	0.126 (0.055, 0.198)	0.066 (0.039, 0.094)	0.106 (0.042, 0.198)	0.062 (0.041, 0.77)	-0.049 (-0.109, 0.002)	0.105
	Cu-IUD V4		0.107 (0.049, 0.165)		0.084 (0.057, 0.161)	-0.005 (-0.187, 0.074)	0.465
**KEMRI**							
ETG-CI V2	ETG-CI V3	0.171 (0.088, 0.255)	0.146 (0.036, 0.256)	0.152 (0.029, 0.278)	0.063 (0.023, 0.144)	0.018 (-0.211, 0.075)	0.855
	ETG-CI V4		0.126 (0.060, 0.192)		0.092 (0.027, 0.194)	-0.025 (-0.133, 0.010)	0.216
DMPA-SC V2	DMPA-SC V3	0.062 (0.024, 0.101)	0.073 (0.031, 0.115)	0.029 (0.012, 0.089)	0.043 (0.018, 0.091)	0.008 (-0.026, 0.055)	0.580
	DMPA-SC V4		0.103 (0.030, 0.176)		0.045 (0.033, 0.140)	0.011 (-0.041, 0.109)	0.521
LNG-IUD V2	LNG-IUD V3	0.083 (0.030, 0.136)	0.154 (0.059, 0.248)	0.055 (0.025, 0.104)	0.092 (0.037, 0.210)	0.030 (-0.035, 0.133)	0.101
	LNG-IUD V4		0.145 (0.0738, 0.216)		0.118 (0.055, 0.177)	0.038 (-0.0003, 0.173)	0.144
Cu-IUD V2	Cu-IUD V3	0.089 (0.039, 0.139)	0.090 (0.039, 0.141)	0.064 (0.018, 0.132)	0.067 (0.024, 0.126)	-0.003 (-0.056, 0.042)	0.934
	Cu-IUD V4		0.082 (0.035, 0.129)		0.061 (0.033, 0.101)	0.009 (-0.056, 0.033)	0.737
**Outcome = [D-Lactic acid] (mg/mL)[§]**							
**EVMS and KEMRI**							
ETG-CI V2	ETG-CI V3	0.043 (0.015, 0.071)	0.058 (0.011, 0.106)	0.008 (0.008, 0.040)	0.008 (0.008, 0.042)	0 (0, 0)	0.862
	ETG-CI V4		0.057 (0.014, 0.099)		0.008 (0.008, 0.045)	0 (-0.008, 0.008)	0.650
DMPA-SC V2	DMPA-SC V3	0.018 (0.001, 0.036)	0.042 (0.013, 0.072)	0.008 (0.008, 0.008)	0.008 (0.008, 0.051)	0 (0, 0.024)	0.063
	DMPA-SC V4		0.035 (0.014, 0.057)		0.008 (0.008, 0.058)	0.004 (0, 0.017)	0.051
LNG-IUD V2	LNG-IUD V3	0.031 (0.003, 0.059)	0.057 (0.014, 0.101)	0.008 (0.008, 0.019)	0.008 (0.008, 0.040)	0 (0, 0.013)	0.067
	LNG-IUD V4		0.086 (0.008, 0.165)		0.008 (0.008, 0.087)	0 (0, 0.056)	0.027
Cu-IUD V2	Cu-IUD V3	0.060 (0.023, 0.097)	0.045 (0.023, 0.067)	0.008 (0.008, 0.065)	0.008 (0.008, 0.040)	0 (-0.014, 0.026)	0.748
	Cu-IUD V4		0.055 (0.018, 0.092)		0.008 (0.008, 0.054)	0 (-0.012, 0.019)	0.879

**EVMS**							
ETG-CI V2	ETG-CI V3	0.067 (0.013, 0.121)	0.039 (-0.005, 0.083)	0.008 (0, 0.126)	0.008 (0, 0.008)	0 (-0.008, 0)	0.337
	ETG-CI V4		0.065 (0.002, 0.128)		0.012 (0.008, 0.067)	0 (-0.008, 0.016)	0.828
DMPA-SC V2	DMPA-SC V3	0.031 (-0.01, 0.072)	0.066 (0.009, 0.122)	0.008 (0.008, 0.017)	0.019 (0.008, 0.081)	0.009 (0, 0.039)	0.031
	DMPA-SC V4		0.058 (0.015, 0.102)		0.016 (0.008, 0.108)	0.004 (0, 0.037)	0.164
LNG-IUD V2	LNG-IUD V3	0.05 (-0.014, 0.114)	0.094 (0.0003, 0.187)	0.008 (0.008, 0.031)	0.008 (0.008, 0.096)	0.008 (-0.007, 0.045)	0.039
	LNG-IUD V4		0.127 (-0.070, 0.324)		0.008 (0, 0.087)	0 (-0.007, 0.056)	0.500
Cu-IUD V2	Cu-IUD V3	0.087 (0.015, 0.158)	0.047 (0.008, 0.085)	0.028 (0.008, 0.191)	0.020 (0.008, 0.086)	-0.010 (-0.159, 0.026)	0.322
	Cu-IUD V4		0.041 (0.009, 0.073)		0.019 (0.008, 0.054)	-0.007 (-0.140, 0.008)	0.258
**KEMRI**							
ETG-CI V2	ETG-CI V3	0.019 (0.002, 0.036)	0.076 (-0.011, 0.163)	0.008 (0.008, 0.008)	0.008 (0.008, 0.042)	0 (0, 0.042)	0.250
	ETG-CI V4		0.047 (-0.019, 0.113)		0.008 (0.008, 0.008)	0 (0, 0)	0.812
DMPA-SC V2	DMPA-SC V3	0.008 (0.001, 0.015)	0.024 (-0.007, 0.055)	0.008 (0, 0.008)	0.008 (0, 0.008)	0 (0, 0.008)	0.609
	DMPA-SC V4		0.016 (0.004, 0.028)		0.008 (0.008, 0.018)	0.004 (0, 0.010)	0.185
LNG-IUD V2	LNG-IUD V3	0.012 (0.005, 0.018)	0.026 (0.003, 0.048)	0.008 (0.008, 0.018)	0.008 (0.008, 0.023)	0 (-0.008, 0.008)	0.656
	LNG-IUD V4		0.052 (0.004, 0.099)		0.008 (0.008, 0.097)	0.004 (0, 0.071)	0.023
Cu-IUD V2	Cu-IUD V3	0.036 (-0.004, 0.076)	0.039 (0.008, 0.071)	0.008 (0.008, 0.021)	0.008 (0.008, 0.089)	0 (0, 0.044)	0.602
	Cu-IUD V4		0.067(-0.001,0.141)		0.018 (0.008, 0.058)	0.009 (0, 0.50)	0.170
**Outcome = [Glycogen] (µg/mL)***							
**EVMS and KEMRI**							
ETG-CI V2	ETG-CI V3	5505 (1497, 9513)	9632 (1313, 17951)	980.70 (1.52)	738.09 (1.53)	0.82 (1.57)	0.751
	ETG-CI V4		8577 (1892, 15263)		1046.99 (1.54)	1.09 (1.62)	0.690
DMPA-SC V2	DMPA-SC V3	7211 (-2255, 16678)	12704 (2330, 23078)	508.83 (1.53)	1295.67 (1.53)	2.41 (1.55)	0.266
	DMPA-SC V4		11430 (180, 22680)		1089.94 (1.56)	1.88 (1.62)	0.372
LNG-IUD V2	LNG-IUD V3	3018 (621, 5415)	5028 (179, 9878)	893.28 (1.52)	866.45 (1.53)	0.91 (1.55)	0.848
	LNG-IUD V4		5317 (-388, 11021)		1172.50 (1.57)	1.50 (1.62)	0.278
Cu-IUD V2	Cu-IUD V3	9815 (-334, 19964)	2902 (-286, 6090)	687.83 (1.53)	463.05 (1.56)	0.70 (1.58)	0.859
	Cu-IUD V4		2829 (20.2, 5639)		687.83 (1.53)	1.16 (1.64)	0.694
**EVMS**							
ETG-CI V2	ETG-CI V3	1210 (500, 1920)	4108 (-3760, 11976)	407.05 (1.83)	227.05 (1.87)	0.42 (1.90)	0.625
	ETG-CI V4		4739 (-1999, 11477)		465.11 (1.83)	0.76 (1.91)	0.687
DMPA-SC V2	DMPA-SC V3	14323 (-8100, 36747)	16209 (-3863, 36281)	864.33 (1.92)	1636.14 (1.93)	2.06 (1.88)	0.656
	DMPA-SC V4		7534 (-2591, 17659)		734.80 (1.92)	1.02 (1.95)	0.821

LNG-IUD V2	LNG-IUD V3	944 (529, 1359)	5581 (-4507, 15669)	557.23 (1.87)	591.36 (1.87)	1.36 (1.87)	0.801
	LNG-IUD V4		7864 (-6917, 22645)		1411.95 (2.05)	3.98 (2.01)	0.378
Cu-IUD V2	Cu-IUD V3	6992 (-4699, 18682)	649 (128, 1169)	684.86 (1.92)	283.58 (2.05)	0.47 (2.03)	0.359
	Cu-IUD V4		934 (430, 1438)		488.43 (1.98)	0.72 (2.02)	0.612
**KEMRI**							
ETG-CI V2	ETG-CI V3	9801 (2040, 17561)	14761 (-175, 29696)	2362.78 (2.76)	2295.55 (1.76)	1.15 (1.91)	0.860
	ETG-CI V4		13056 (17.3, 26094)		2698.15 (1.84)	1.51 (2.06)	0.784
DMPA-SC V2	DMPA-SC V3	1521 (67.6, 2975)	9900 (-2157, 21957)	333.04 (1.72)	1075.06 (1.72)	2.75 (1.85)	0.307
	DMPA-SC V4		15027 (-6112, 36166)		1568.42 (1.79)	3.16 (2.02)	0.168
LNG-IUD V2	LNG-IUD V3	4815 (322, 9308)	4515 (741, 8290)	1344.66 (1.72)	1235.34 (1.76)	0.80 (1.88)	0.785
	LNG-IUD V4		3497 (992, 6001)		996.99 (1.76)	0.74 (1.95)	0.802
Cu-IUD V2	Cu-IUD V3	12073 (-4942, 29089)	4404 (-1018, 9827)	690.21 (1.72)	642.09 (1.72)	0.93 (1.83)	0.558
	Cu-IUD V4		4433 (-914, 9779)		878.65 (1.79)	1.83 (1.99)	0.453
**Outcome = [RANTES] (pg/mL)**							
**EVMS and KEMRI**							
ETG-CI V2	ETG-CI V3	129 (1.15, 257)	116 (9.62, 222)	0.9038 (0.2037)	1.1715 (0.1996)	0.2677 (0.3121)	0.3985
	ETG-CI V4		310 (-151, 772)		1.0604 (0.2396)	0.1566 (0.3163)	0.6245
DMPA-SC V2	DMPA-SC V3	295 (-58.7, 648)	89 (-3.17, 181)	0.9724 (0.2156)	0.7615 (0.2472)	-0.2108 (0.2888)	0.4719
	DMPA-SC V4		2117 (-209, 4443)		0.9535 (0.3177)	-0.01885 (0.2832)	0.9475
LNG-IUD V2	LNG-IUD V3	309 (-15, 634)	538 (54.1, 1022)	1.1412 (0.2256)	1.7253 (0.2026)	0.5841 (0.2982)	0.0605
	LNG-IUD V4		3283 (-2873, 9438)		1.1880 (0.2653)	0.04685 (0.3110)	0.8815
Cu-IUD V2	Cu-IUD V3	546 (-37.9, 1130)	705 (-54.7, 1466)	1.2453 (0.2610)	1.7054 (0.2221)	0.4601 (0.3163)	0.1576
	Cu-IUD V4		165 (-27.4, 358)		1.3404 (0.1725)	0.09515 (0.2981)	0.7522
**EVMS**							
ETG-CI V2	ETG-CI V3	180 (-69.3, 429)	136 (-85.2, 358)	0.6184 (0.3424)	0.8486 (0.3328)	0.2302 (0.5083)	0.6580
	ETG-CI V4		30.7 (-22.8, 84.2)		0.4182 (0.2549)	-0.2003 (0.4631)	0.6725
DMPA-SC V2	DMPA-SC V3	395 (-302, 1092)	40 (-31, 111)	0.8397 (0.3685)	0.1478 (0.4307)	-0.6918 (0.5047)	0.1978
	DMPA-SC V4		1862 (-1720, 5445)		0.6577 (0.5257)	-0.1820 (0.3249)	0.5867
LNG-IUD V2	LNG-IUD V3	157 (-173, 487)	117 (-3.94, 238)	0.4861 (0.3000)	1.3618 (0.2626)	0.8757 (0.4102)	0.0541
	LNG-IUD V4		7779 (-8289, 23846)		1.4748 (0.4630)	0.9887 (0.3690)	0.0252
Cu-IUD V2	Cu-IUD V3	67.4 (-11.2, 146)	784 (-204, 1772)	0.6578 (0.3497)	1.8749 (0.3712)	1.2170 (0.5050)	0.0351
	Cu-IUD V4		305 (-158, 768)		1.4762 (0.3074)	0.8184 (0.4888)	0.1268

**KEMRI**							
ETG-CI V2	ETG-CI V3	79 (-29.9, 188)	96.8 (16.4, 177)	1.1891 (0.2065)	1.4651 (0.2124)	0.2760 (0.3829)	0.4839
	ETG-CI V4		637 (-415, 1688)		1.8039 (0.3123)	0.6148 (0.4075)	0.1554
DMPA-SC V2	DMPA-SC V3	215 (-185, 614)	128 (-35.4, 292)	1.0785 (0.2611)	1.2525 (0.2211)	0.1740 (0.3098)	0.5833
	DMPA-SC V4		2336 (-1165, 5837)		1.1890 (0.3965)	0.1105 (0.4497)	0.8096
LNG-IUD V2	LNG-IUD V3	441 (-127, 1010)	903 (-0.766, 1807)	1.7089 (0.2585)	2.0404 (0.2851)	0.3315 (0.4285)	0.4520
	LNG-IUD V4		70.8 (-20.2, 162)		0.8915 (0.2721)	-0.8174 (0.3498)	0.0352
Cu-IUD V2	Cu-IUD V3	961 (-141, 2064)	653 (-532, 1838)	1.7544 (0.3384)	1.6026 (0.2862)	-0.1518 (0.3553)	0.6756
	Cu-IUD V4		55.6 (6.42, 105)		1.2428 (0.2008)	-0.5117 (0.3232)	0.1353
**Outcome = [SLPI] (pg/mL)**							
**EVMS and KEMRI**							
ETG-CI V2	ETG-CI V3	410476 (266962, 553990)	236403 (94383, 378423)	5.3557 (0.1096)	5.0481 (0.1082)	-0.3076 (0.1369)	0.0330
	ETG-CI V4		249357 (92391, 406322)		5.0677 (0.1100)	-0.2879 (0.1193)	0.0237
DMPA-SC V2	DMPA-SC V3	357290 (150287, 564292)	159023 (89054, 228991)	5.2156 (0.1094)	5.0210 (0.07631)	-0.1947 (0.1149)	0.1021
	DMPA-SC V4		237984 (174304, 301663)		5.2742 (0.06273)	0.05853 (0.1251)	0.6441
LNG-IUD V2	LNG-IUD V3	333625 (221275, 445974)	347803 (210921, 484685)	5.3100 (0.1081)	5.2582 (0.1068)	-0.05180 (0.1141)	0.6534
	LNG-IUD V4		392321 (169677, 614965)		5.3243 (0.1022)	0.01430 (0.1186)	0.9050
Cu-IUD V2	Cu-IUD V3	302525 (186951, 418098)	331270 (183332, 479209)	5.2277 (0.1058)	5.3061 (0.09463)	0.07844 (0.1353)	0.5668
	Cu-IUD V4		502972 (260935, 745008)		5.4937 (0.08163)	0.2661 (0.1088)	0.0218
**EVMS**							
ETG-CI V2	ETG-CI V3	356099 (168188, 544010)	173299 (52614, 293985)	5.2768 (0.1680)	5.0243 (0.1245)	-0.2525 (0.1936)	0.2143
	ETG-CI V4		122374 (50886, 193862)		4.9460 (0.08984)	-0.3309 (0.1524)	0.0491
DMPA-SC V2	DMPA-SC V3	315482 (44846, 586118)	205181 (48922, 361440)	5.1911 (0.1666)	5.0909 (0.1296)	-0.1003 (0.1842)	0.5971

	DMPA-SC V4		221414 (137308, 305520)		5.2743 (0.07504)	0.08314 (0.1719)	0.6381
LNG-IUD V2	LNG-IUD V3	426384 (202051, 650718)	317251 (117921, 516581)	5.3207 (0.2150)	5.1666 (0.1718)	-0.1541 (0.2078)	0.4725
	LNG-IUD V4		303429 (93613, 513245)		5.3027 (0.1450)	-0.01802 (0.1777)	0.9209
Cu-IUD V2	Cu-IUD V3	306371 (84049, 528693)	502923 (140925, 864921)	5.1556 (0.1813)	5.4640 (0.1613)	0.3085 (0.2151)	0.1779
	Cu-IUD V4		324773 (176510, 473037)		5.4072 (0.09631)	0.2517 (0.1939)	0.2198
**KEMRI**							
ETG-CI V2	ETG-CI V3	464853 (225249, 704458)	294999 (28350, 561647)	5.4345 (0.1438)	5.0713 (0.1787)	-0.3632 (0.1976)	0.0891
	ETG-CI V4		397503 (60906, 734101)		5.1898 (0.2211)	-0.2447 (0.1989)	0.2449
DMPA-SC V2	DMPA-SC V3	390736 (56960, 724512)	122096 (74817, 169375)	5.2352 (0.1500)	4.9651 (0.09170)	-0.2702 (0.1489)	0.0911
	DMPA-SC V4		252186 (148094, 356279)		5.2808 (0.1005)	0.04558 (0.1878)	0.8124
LNG-IUD V2	LNG-IUD V3	253233 (159153, 347314)	374282 (163083, 585481)	5.3006 (0.08761)	5.3375 (0.1347)	0.03686 (0.1191)	0.7615
	LNG-IUD V4		455815 (80790, 830840)		5.3413 (0.1429)	0.04062 (0.1600)	0.8034
Cu-IUD V2	Cu-IUD V3	299191 (168981, 429402)	216836 (137545, 296126)	5.2902 (0.1236)	5.1982 (0.1106)	-0.09196 (0.1686)	0.5941
	Cu-IUD V4		642984 (215062, 1070906)		5.5890 (0.1232)	0.2988 (0.1211)	0.0275
**Outcome = [β-Defensin-2] (pg/mL)**							
**EVMS and KEMRI**							
ETG-CI V2	ETG-CI V3	123364 (30995, 215733)	280828 (-122179, 683836)	4.447 (0.1850)	4.4352 (0.1822)	-0.01180 (0.2536)	0.9632
	ETG-CI V4		95401 (29109, 161692)		4.4205 (0.1576)	-0.02655 (0.2030)	0.8969

DMPA-SC V2	DMPA-SC V3	155104 (18546, 291662)	121869 (21684, 222054)	4.2303 (0.2040)	4.3873 (0.1657)	0.1569 (0.2068)	0.4547
	DMPA-SC V4		83037 (11818, 154256)		4.2518 (0.1530)	0.02145 (0.1495)	0.8870
LNG-IUD V2	LNG-IUD V3	58644 (2021, 115267)	139894 (8211, 271576)	4.0738 (0.1719)	4.2848 (0.2073)	0.2109 (0.2067)	0.3166
	LNG-IUD V4		147381 (22103, 272659)		4.4566 (0.1875)	0.3828 (0.2108)	0.0811
Cu-IUD V2	Cu-IUD V3	68754 (26637, 110870)	41628 (18973, 64283)	4.2313 (0.1761)	4.2621 (0.1423)	0.03081 (0.2040)	0.8812
	Cu-IUD V4		75110 (15575, 134646)		4.3995 (0.1490)	0.1682 (0.1979)	0.4032
**EVMS**							
ETG-CI V2	ETG-CI V3	145187 (-38746, 329120)	529252 (-349781, 1408285)	4.3471 (0.3087)	4.5860 (0.3190)	0.2389 (0.3997)	0.5605
	ETG-CI V4		60071 (11275, 108868)		4.3922 (0.1645)	0.04513 (0.2733)	0.8714
DMPA-SC V2	DMPA-SC V3	194968 (15487, 374449)	194115 (-24579, 412809)	4.5274 (0.3057)	4.6411 (0.2523)	0.1137 (0.3231)	0.7315
	DMPA-SC V4		130872 (-28101, 289846)		4.4260 (0.2394)	-0.1014 (0.1984)	0.6196
LNG-IUD V2	LNG-IUD V3	103253 (-22182, 228687)	121148 (7570, 234726)	4.3331 (0.2835)	4.4715 (0.2571)	0.1383 (0.2812)	0.6315
	LNG-IUD V4		73281 (-12011, 158572)		4.0433 (0.3025)	-0.2898 (0.3161)	0.3793
Cu-IUD V2	Cu-IUD V3	86125 (864, 171387)	26096 (10451, 41741)	4.2634 (0.2739)	4.1970 (0.1660)	-0.06638 (0.2708)	0.8110
	Cu-IUD V4		36370 (15608, 57131)		4.3386 (0.1823)	0.07525 (0.3348)	0.8263
**KEMRI**							
ETG-CI V2	ETG-CI V3	101542 (30015, 173068)	50149 (16007, 84292)	4.5470 (0.2128)	4.3052 (0.1962)	-0.2418 (0.3193)	0.4623
	ETG-CI V4		136618 (-4511, 277747)		4.4485 (0.2948)	-0.09854 (0.3280)	0.7690
DMPA-SC V2	DMPA-SC V3	123212 (-95625, 342050)	64071 (-10177, 138320)	3.9927 (0.2669)	4.1842 (0.2125)	0.1915 (0.2797)	0.5048
	DMPA-SC V4		42035 (10972, 73099)		4.1190 (0.1977)	0.1263 (0.2256)	0.5846

LNG-IUD V2	LNG-IUD V3	19983 (4879, 35087)	156140 (-86216, 398497)	3.8491 (0.1974)	4.1230 (0.3187)	0.2739 (0.3098)	0.3916
	LNG-IUD V4		200310 (-14680, 415299)		4.7466 (0.2085)	0.8975 (0.2107)	0.0009
Cu-IUD V2	Cu-IUD V3	53698 (13896, 93500)	51982 (14549, 89415)	4.2035 (0.2362)	4.3000 (0.2144)	0.09652 (0.2944)	0.7479
	Cu-IUD V4		105550 (-3088, 214187)		4.4776 (0.2274)	0.2741 (0.2461)	0.2844

LSM: Least Squares Mean. *LSM were calculated using $\log_{10}$mean values for each analyte. [§]Median and Interquartile Range reported instead of LSM and S.E.

Supplementary Table S7. Immunohistochemical analysis of vaginal epithelium: comparison between trial arms.

Reference visit	Comparator visit	Mean Reference (95% CI)	Mean Comparator (95% CI)	LSM Reference (S.E.)	LSM Comparator (S.E.)	LSMs Difference (S.E.)	p-value
**Outcome = Number of epithelial layers***							
**EVMS and KEMRI**							
ETG-CI V2	ETG-CI V3	32.51 (30.35, 34.67)	27.24 (25.12, 29.37)	32.09 (1.04)	26.77 (1.04)	0.84 (1.05)	0.001
	ETG-CI V4		33.68 (30.55, 36.82)		32.83 (1.04)	1.05 (1.05)	0.723
DMPA-SC V2	DMPA-SC V3	30.95 (27.53, 34.38)	30.47 (27.7, 33.25)	29.78 (1.04)	29.71 (1.04)	1.01 (1.05)	0.258
	DMPA-SC V4		33.73 (30.23, 37.23)		32.66 (1.05)	1.10 (1.05)	0.320
LNG-IUD V2	LNG-IUD V3	30.81 (27.73, 33.89)	32.62 (29.64, 35.59)	29.87 (1.04)	31.72 (1.04)	1.06 (1.05)	0.261
	LNG-IUD V4		33.03 (30.3, 35.76)		32.47 (1.05)	1.05 (1.05)	0.295
Cu-IUD V2	Cu-IUD V3	28.96 (26.23, 31.69)	29.58 (27.41, 31.76)	28.03 (1.04)	29.10 (1.04)	1.05 (1.05)	0.499
	Cu-IUD V4		31.92 (30.05, 33.79)		31.62 (1.05)	1.10 (1.05)	0.048
**EVMS**							
ETG-CI V2	ETG-CI V3	34.5 (30.8, 38.1)	30.4 (27.2, 33.6)	34.00 (1.06)	30.03 (1.06)	0.88 (1.07)	0.113
	ETG-CI V4		36.4 (31.6, 41.2)		35.42 (1.06)	1.10 (1.08)	0.499
DMPA-SC V2	DMPA-SC V3	32.9 (27.6, 38.3)	34.9 (31.4, 38.3)	32.01 (1.07)	34.53 (1.07)	1.10 (1.07)	0.600
	DMPA-SC V4		36.7 (30.5, 42.8)		35.72 (1.07)	1.18 (1.09)	0.405
LNG-IUD V2	LNG-IUD V3	32.4 (26.6, 38.3)	35.4 (29.6, 41.3)	31.06 (1.07)	34.21 (1.07)	1.08 (1.08)	0.357
	LNG-IUD V4		35.3 (30.2, 40.5)		34.76 (1.07)	1.03 (1.09)	0.759
Cu-IUD V2	Cu-IUD V3	28.6 (24.3, 33)	29.7 (26.4, 33)	27.56 (1.07)	29.40 (1.07)	1.10 (1.08)	0.363
	Cu-IUD V4		32.3 (28.4, 36.3)		31.89 (1.07)	1.12 (1.09)	0.133
**KEMRI**							
ETG-CI V2	ETG-CI V3	30.54 (28.23, 32.84)	24.29 (22.24, 26.33)	30.29 (1.06)	24.06 (1.06)	0.79 (1.07)	0.001
	ETG-CI V4		30.54 (26.8, 34.28)		30.05 (1.06)	0.99 (1.06)	0.759
DMPA-SC V2	DMPA-SC V3	29.26 (24.37, 34.15)	27.25 (23.79, 30.71)	27.99 (1.06)	26.60 (1.05)	0.96 (1.07)	0.495
	DMPA-SC V4		31.64 (27.22, 36.07)		30.64 (1.06)	1.05 (1.06)	0.508
LNG-IUD V2	LNG-IUD V3	29.53 (25.94, 33.12)	30.54 (27.42, 33.66)	28.95 (1.05)	30.00 (1.05)	1.04 (1.07)	0.403
	LNG-IUD V4		31.39 (28.16, 34.63)		30.92 (1.06)	1.07 (1.06)	0.273
Cu-IUD V2	Cu-IUD V3	29.24 (25.29, 33.19)	29.49 (26.28, 32.71)	28.41 (1.05)	28.91 (1.05)	1.02 (1.07)	0.796
	Cu-IUD V4		31.62 (29.5, 33.74)		31.43 (1.06)	1.08 (1.05)	0.421
**Outcome = Epithelial thickness (µm)***							
**EVMS and KEMRI**							
ETG-CI V2	ETG-CI V3	341.8 (298.8, 384.8)	269.9 (239.1, 300.7)	326.24 (1.07)	258.11 (1.07)	0.81 (1.08)	0.021

	ETG-CI V4		371.8 (325.1, 418.5)		354.50 (1.07)	1.09 (1.08)	0.352
DMPA-SC V2	DMPA-SC V3	346.9 (298.8, 395.0.0)	313.8 (267.8, 359.8)	324.92 (1.07)	295.64 (1.07)	0.92 (1.08)	0.324
	DMPA-SC V4		330.2 (283.4, 376.9)		311.76 (1.07)	1.00 (1.08)	0.655
LNG-IUD V2	LNG-IUD V3	331.6 (270.2, 393.0)	350.8 (304.4, 397.1)	301.52 (1.07)	331.41 (1.07)	1.07 (1.08)	0.371
	LNG-IUD V4		348.8 (304.3, 393.2)		336.19 (1.07)	1.07 (1.08)	0.564
Cu-IUD V2	Cu-IUD V3	283.0 (247.1, 318.9)	283.9 (250.9, 316.9)	264.20 (1.07)	273.14 (1.07)	1.03 (1.08)	0.805
	Cu-IUD V4		313.9 (277.5, 350.2)		302.91 (1.07)	1.12 (1.08)	0.407
**EVMS**							
ETG-CI V2	ETG-CI V3	378.6 (316.8, 440.3)	316.4 (281.5, 351.3)	367.06 (1.09)	311.16 (1.09)	0.87 (1.11)	0.219
	ETG-CI V4		422.8 (355.6, 490.0)		407.88 (1.09)	1.19 (1.10)	0.229
DMPA-SC V2	DMPA-SC V3	407.8 (339.2, 476.4)	390.4 (309.3, 471.5)	395.31 (1.10)	374.86 (1.10)	0.96 (1.12)	0.645
	DMPA-SC V4		365.8 (291.1, 440.6)		352.71 (1.11)	0.95 (1.11)	0.391
LNG-IUD V2	LNG-IUD V3	386.5 (260.3, 512.7)	392.6 (319.8, 465.3)	341.73 (1.10)	379.71 (1.10)	1.01 (1.13)	0.219
	LNG-IUD V4		378.7 (286.1, 471.2)		363.10 (1.10)	0.93 (1.11)	0.229
Cu-IUD V2	Cu-IUD V3	289.6 (229.4, 349.9)	286.0 (226.5, 345.4)	266.86 (1.10)	276.60 (1.11)	1.04 (1.13)	0.833
	Cu-IUD V4		341.4 (265.6, 417.1)		325.64 (1.11)	1.17 (1.11)	0.322
**KEMRI**							
ETG-CI V2	ETG-CI V3	305.1 (244.0, 366.2)	226.7 (186.5, 266.9)	289.95 (1.09)	216.98 (1.09)	0.73 (1.11)	0.063
	ETG-CI V4		312.4 (258.3, 366.5)		300.99 (1.10)	0.97 (1.12)	0.672
DMPA-SC V2	DMPA-SC V3	294.7 (233.9, 355.4)	257.7 (219.5, 295.9)	274.66 (1.09)	248.40 (1.09)	0.92 (1.11)	0.418
	DMPA-SC V4		304.7 (240.3, 369.1)		285.46 (1.09)	1.06 (1.11)	0.851
LNG-IUD V2	LNG-IUD V3	287.7 (234.9, 340.5)	320.1 (257.5, 382.7)	272.78 (1.09)	299.94 (1.09)	1.11 (1.10)	0.375
	LNG-IUD V4		327.4 (279.6, 375.2)		318.19 (1.09)	1.19 (1.11)	0.298
Cu-IUD V2	Cu-IUD V3	277.3 (228.0, 326.6)	282.5 (237.8, 327.1)	262.09 (1.09)	270.86 (1.09)	1.03 (1.10)	0.848
	Cu-IUD V4		294.3 (256.2, 332.3)		287.66 (1.09)	1.07 (1.11)	0.745
**Outcome = $CD45^{+}$cells/mm^{2}***							
**EVMS and KEMRI**							
ETG-CI V2	ETG-CI V3	137.2 (113.1, 161.3)	179.8 (133.8, 225.8)	124.81 (1.10)	157.73 (1.10)	1.27 (1.11)	0.151
	ETG-CI V4		127.6 (106.4, 148.8)		118.09 (1.10)	0.95 (1.22)	0.646
DMPA-SC V2	DMPA-SC V3	124.4 (92.85, 156.0)	140.5 (120.1, 161)	105.65 (1.10)	133.39 (1.10)	1.23 (1.11)	0.050
	DMPA-SC V4		148.0 (119.1, 176.9)		136.23 (1.10)	1.13 (1.12)	0.117
LNG-IUD V2	LNG-IUD V3	126.1 (101.0, 151.1)	110.5 (94.32, 126.7)	111.50 (1.10)	102.71 (1.10)	0.92 (1.11)	0.689

	LNG-IUD V4		132.5 (99.11, 165.9)		114.17 (1.10)	0.98 (1.12)	0.783
Cu-IUD V2	Cu-IUD V3	159.2 (118.0, 200.4)	149.9 (109.4, 190.3)	135.23 (1.10)	127.10 (1.10)	1.00 (1.11)	0.759
	Cu-IUD V4		134.1 (107.6, 160.6)		122.21 (1.10)	0.88 (1.12)	0.044
**EVMS**							
ETG-CI V2	ETG-CI V3	127.9 (89.85, 165.9)	143.7 (112.4, 175.0)	114.15 (1.13)	135.64 (1.13)	1.20 (1.14)	0.339
	ETG-CI V4		117.3 (85.52, 149.0)		108.02 (1.13)	0.89 (1.18)	0.658
DMPA-SC V2	DMPA-SC V3	104.2 (79.47, 128.8)	136.4 (101.5, 171.3)	98.45 (1.14)	128.73 (1.15)	1.27 (1.15)	0.089
	DMPA-SC V4		161.8 (105.9, 217.6)		148.16 (1.15)	1.36 (1.21)	0.035
LNG-IUD V2	LNG-IUD V3	126.7 (81.15, 172.2)	112.3 (80.45, 144.2)	108.81 (1.14)	101.19 (1.15)	0.93 (1.16)	0.825
	LNG-IUD V4		116.9 (88.82, 144.9)		109.04 (1.15)	0.96 (1.21)	0.844
Cu-IUD V2	Cu-IUD V3	176.1 (97.92, 254.3)	161.4 (117.6, 205.2)	150.55 (1.14)	151.28 (1.15)	1.15 (1.16)	0.782
	Cu-IUD V4		145.2 (89.16, 201.3)		130.54 (1.15)	0.85 (1.21)	0.663
**KEMRI**							
ETG-CI V2	ETG-CI V3	146.5 (112.4, 180.6)	213.3 (127.5, 299.1)	114.15 (1.13)	135.64 (1.13)	1.34 (1.18)	0.208
	ETG-CI V4		139.7 (108.7, 170.6)		108.02 (1.13)	1.05 (1.18)	0.850
DMPA-SC V2	DMPA-SC V3	156.1 (92.74, 219.4)	143.6 (115.1, 172.1)	112.24 (1.15)	136.91 (1.14)	1.18 (1.18)	0.309
	DMPA-SC V4		138.2 (102.6, 173.9)		128.31 (1.15)	0.95 (1.17)	0.790
LNG-IUD V2	LNG-IUD V3	132.4 (98.89, 166)	109.2 (89.53, 128.9)	113.70 (1.14)	103.83 (1.14)	0.92 (1.17)	0.663
	LNG-IUD V4		143.7 (86.86, 200.5)		117.98 (1.15)	0.97 (1.16)	0.806
Cu-IUD V2	Cu-IUD V3	144.5 (98.44, 190.6)	142.2 (76.94, 207.5)	124.11 (1.14)	113.17 (1.14)	0.91 (1.17)	0.576
	Cu-IUD V4		126.2 (97.05, 155.3)		116.59 (1.15)	0.91 (1.16)	0.729
**Outcome = $CD3^{+}$cells/mm^{2*}**							
**EVMS and KEMRI**							
ETG-CI V2	ETG-CI V3	102.4 (83.01, 121.8)	130.1 (94.26, 165.9)	92.09 (1.11)	112.30 (1.11)	1.21 (1.12)	0.293
	ETG-CI V4		96.73 (78.55, 114.9)		87.49 (1.11)	0.93 (1.14)	0.710
DMPA-SC V2	DMPA-SC V3	91.35 (66.91, 115.8)	111.8 (93.45, 130.2)	74.99 (1.11)	104.33 (1.11)	1.32 (1.13)	0.049
	DMPA-SC V4		108.3 (82.84, 133.7)		95.78 (1.12)	1.09 (1.14)	0.337
LNG-IUD V2	LNG-IUD V3	92.78 (73.53, 112.0)	81.16 (66.66, 95.66)	80.67 (1.11)	72.84 (1.11)	0.90 (1.13)	0.619
	LNG-IUD V4		97.04 (70.76, 123.3)		80.51 (1.12)	0.93 (1.14)	0.838
Cu-IUD V2	Cu-IUD V3	120.5 (84.74, 156.2)	111.7 (85.51, 137.9)	98.56 (1.11)	94.60 (1.11)	1.05 (1.13)	0.846
	Cu-IUD V4		96.12 (73.05, 119.2)		84.89 (1.12)	0.85 (1.14)	0.413
**EVMS**							
ETG-CI V2	ETG-CI V3	96.21 (62.15, 130.3)	105.4 (78.57, 132.2)	83.25 (1.15)	97.37 (1.16)	1.18 (1.17)	0.509

	ETG-CI V4		91.99 (63.0, 121.0)		82.15 (1.15)	0.91 (1.21)	0.799
DMPA-SC V2	DMPA-SC V3	79.25 (53.84, 104.7)	109.2 (75.44, 142.9)	71.39 (1.17)	100.67 (1.18)	1.26 (1.17)	0.126
	DMPA-SC V4		125.1 (72.07, 178.1)		108.43 (1.19)	1.30 (1.24)	0.112
LNG-IUD V2	LNG-IUD V3	102.2 (64.34, 140.1)	89.33 (58.34, 120.3)	84.86 (1.17)	76.51 (1.18)	0.89 (1.19)	0.733
	LNG-IUD V4		89.23 (62.45, 116.0)		80.46 (1.19)	0.86 (1.24)	0.732
Cu-IUD V2	Cu-IUD V3	145.7 (72.14, 219.3)	124.9 (89.49, 160.3)	118.67 (1.17)	115.63 (1.19)	1.17 (1.18)	0.820
	Cu-IUD V4		112.2 (61.94, 162.5)		98.04 (1.19)	0.83 (1.23)	0.508
**KEMRI**							
ETG-CI V2	ETG-CI V3	108.6 (85.28, 131.8)	153.1 (86.17, 219.9)	101.88 (1.16)	128.20 (1.16)	1.26 (1.20)	0.339
	ETG-CI V4		102.3 (77.14, 127.4)		94.15 (1.17)	1.01 (1.20)	0.785
DMPA-SC V2	DMPA-SC V3	101.7 (59.14, 144.3)	113.8 (89.71, 137.8)	78.21 (1.16)	107.10 (1.15)	1.33 (1.19)	0.257
	DMPA-SC V4		96.22 (68.96, 123.5)		87.65 (1.16)	0.92 (1.84)	0.841
LNG-IUD V2	LNG-IUD V3	85.25 (63.99, 106.5)	75.17 (60.62, 89.73)	77.46 (1.15)	70.26 (1.15)	0.91 (1.18)	0.697
	LNG-IUD V4		102.6 (58.64, 146.6)		80.54 (1.16)	0.97 (1.18)	0.737
Cu-IUD V2	Cu-IUD V3	98.60 (70.51, 126.7)	102.9 (63.29, 142.4)	84.95 (1.15)	82.76 (1.15)	0.97 (1.18)	0.768
	Cu-IUD V4		84.60 (62.13, 107.1)		76.59 (1.16)	0.87 (1.17)	0.519
**Outcome = CD1a$^+$cells/mm^2***							
**EVMS and KEMRI**							
ETG-CI V2	ETG-CI V3	40.55 (32.93, 48.17)	41.89 (34.81, 48.96)	36.05 (1.10)	38.36 (1.10)	1.09 (1.12)	0.772
	ETG-CI V4		29.20 (23.08, 35.31)		26.06 (1.10)	0.76 (1.11)	0.019
DMPA-SC V2	DMPA-SC V3	38.73 (30.16, 47.3)	33.82 (26.91, 40.73)	33.76 (1.10)	30.02 (1.10)	0.85 (1.12)	0.539
	DMPA-SC V4		35.67 (29.34, 41.99)		32.71 (1.10)	0.88 (1.11)	0.826
LNG-IUD V2	LNG-IUD V3	33.52 (28.42, 38.63)	30.83 (23.44, 38.23)	31.09 (1.10)	26.47 (1.10)	0.87 (1.12)	0.213
	LNG-IUD V4		33.31 (26.29, 40.34)		29.07 (1.10)	0.99 (1.10)	0.735
Cu-IUD V2	Cu-IUD V3	37.42 (30.54, 44.29)	41.10 (33.38, 48.81)	35.63 (1.10)	37.64 (1.10)	1.07 (1.12)	0.652
	Cu-IUD V4		39.01 (31.81, 46.21)		35.65 (1.10)	0.96 (1.11)	0.813
**EVMS**							
ETG-CI V2	ETG-CI V3	38.53 (29.94, 47.11)	33.79 (26.62, 40.97)	36.19 (1.14)	31.82 (1.14)	0.90 (1.18)	0.440
	ETG-CI V4		24.84 (18.88, 30.79)		22.83 (1.13)	0.62 (1.14)	0.010
DMPA-SC V2	DMPA-SC V3	35.89 (25.11, 46.67)	29.61 (21.36, 37.86)	32.70 (1.15)	27.17 (1.15)	0.75 (1.18)	0.468
	DMPA-SC V4		35.00 (24.83, 45.16)		32.57 (1.16)	0.93 (1.16)	0.835
LNG-IUD V2	LNG-IUD V3	34.75 (24.32, 45.17)	27.25 (18.17, 36.34)	31.07 (1.14)	23.63 (1.15)	0.82 (1.19)	0.297

	LNG-IUD V4		29.36 (22.47, 36.26)		27.13 (1.16)	1.00 (1.15)	0.699
Cu-IUD V2	Cu-IUD V3	38.30 (25.21, 51.38)	48.33 (32.83, 63.83)	37.75 (1.15)	44.01 (1.17)	1.21 (1.19)	0.434
	Cu-IUD V4		37.75 (22.97, 52.52)		33.65 (1.17)	0.85 (1.16)	0.775
**KEMRI**							
ETG-CI V2	ETG-CI V3	42.42 (28.91, 55.94)	49.41 (38.11, 60.71)	35.92 (1.14)	45.63 (1.14)	1.29 (1.17)	0.398
	ETG-CI V4		34.28 (22.53, 46.03)		30.40 (1.15)	0.92 (1.17)	0.415
DMPA-SC V2	DMPA-SC V3	40.96 (26.98, 54.94)	36.91 (25.96, 47.85)	34.62 (1.14)	32.30 (1.14)	0.91 (1.16)	0.763
	DMPA-SC V4		36.14 (26.89, 45.4)		32.81 (1.14)	0.86 (1.16)	0.815
LNG-IUD V2	LNG-IUD V3	32.55 (27.12, 37.97)	33.46 (21.71, 45.21)	31.10 (1.14)	28.77 (1.14)	0.92 (1.16)	0.508
	LNG-IUD V4		36.14 (24.52, 47.75)		30.54 (1.14)	1.00 (1.15)	0.865
Cu-IUD V2	Cu-IUD V3	36.71 (28.38, 45.05)	36.75 (27.8, 45.71)	34.14 (1.14)	34.27 (1.13)	1.00 (1.15)	0.869
	Cu-IUD V4		39.82 (30.89, 48.76)		36.99 (1.14)	0.85 (1.16)	0.629
**Outcome = $CD8^+$cells/mm^2***							
**EVMS and KEMRI**							
ETG-CI V2	ETG-CI V3	79.08 (62.87, 95.3)	97.35 (68.93, 125.8)	69.63 (1.12)	81.79 (1.12)	1.14 (1.14)	0.450
	ETG-CI V4		73.18 (59.01, 87.36)		65.39 (1.12)	0.92 (1.14)	0.666
DMPA-SC V2	DMPA-SC V3	67.81 (49.19, 86.42)	84.84 (69.32, 100.4)	54.08 (1.12)	77.14 (1.12)	1.36 (1.14)	0.078
	DMPA-SC V4		83.76 (64.92, 102.6)		74.17 (1.12)	1.18 (1.14)	0.208
LNG-IUD V2	LNG-IUD V3	68.51 (53.28, 83.73)	59.58 (48.92, 70.23)	58.40 (1.12)	53.63 (1.12)	0.95 (1.14)	0.603
	LNG-IUD V4		71.00 (50.78, 91.23)		59.32 (1.13)	0.95 (1.14)	0.853
Cu-IUD V2	Cu-IUD V3	93.76 (64.23, 123.3)	82.22 (59.93, 104.5)	74.26 (1.12)	65.48 (1.12)	0.96 (1.14)	0.689
	Cu-IUD V4		67.03 (47.88, 86.17)		57.02 (1.13)	0.75 (1.14)	0.162
**EVMS**							
ETG-CI V2	ETG-CI V3	73.77 (44.16, 103.4)	75.18 (53.14, 97.23)	60.97 (1.17)	67.80 (1.18)	1.10 (1.17)	0.653
	ETG-CI V4		67.42 (44.74, 90.1)		58.65 (1.17)	0.89 (1.22)	0.764
DMPA-SC V2	DMPA-SC V3	55.19 (35.3, 75.08)	88.61 (57.42, 119.8)	47.34 (1.18)	78.46 (1.19)	1.53 (1.17)	0.095
	DMPA-SC V4		95.90 (57.33, 134.5)		82.62 (1.20)	1.60 (1.25)	0.096
LNG-IUD V2	LNG-IUD V3	79.58 (50.98, 108.2)	65.63 (45.33, 85.93)	67.68 (1.18)	58.13 (1.19)	0.90 (1.19)	0.588
	LNG-IUD V4		63.91 (46.05, 81.78)		59.44 (1.20)	0.79 (1.25)	0.524
Cu-IUD V2	Cu-IUD V3	115.6 (55.17, 176.1)	92.51 (61.1, 123.9)	91.03 (1.18)	82.55 (1.20)	1.08 (1.18)	0.812
	Cu-IUD V4		82.50 (38.2, 126.8)		68.04 (1.20)	0.73 (1.25)	0.352
**KEMRI**							
ETG-CI V2	ETG-CI V3	84.39 (66.67, 102.1)	117.9 (65.81, 170.1)	79.52 (1.17)	97.34 (1.17)	1.20 (1.22)	0.386

	ETG-CI V4		79.91 (61, 98.81)		74.25 (1.18)	0.98 (1.19)	0.755
DMPA-SC V2	DMPA-SC V3	78.62 (47.09, 110.1)	82.07 (63.9, 100.2)	60.63 (1.17)	76.18 (1.16)	1.22 (1.22)	0.502
	DMPA-SC V4		75.10 (54.15, 96.05)		68.67 (1.17)	0.93 (1.17)	0.847
LNG-IUD V2	LNG-IUD V3	59.65 (42.41, 76.88)	55.14 (42.26, 68.02)	51.89 (1.16)	50.55 (1.16)	0.99 (1.21)	0.779
	LNG-IUD V4		76.06 (41.61, 110.5)		59.24 (1.17)	1.08 (1.17)	0.618
Cu-IUD V2	Cu-IUD V3	74.82 (51.44, 98.2)	75.36 (41.97, 108.7)	63.10 (1.16)	56.11 (1.16)	0.87 (1.21)	0.713
	Cu-IUD V4		55.97 (41.07, 70.87)		50.26 (1.17)	0.76 (1.16)	0.413
**Outcome = HLA-DR$^+$cells/mm^2***							
**EVMS and KEMRI**							
ETG-CI V2	ETG-CI V3	43.78 (36.97, 50.59)	48.39 (39.37, 57.41)	40.02 (1.09)	43.63 (1.09)	1.09 (1.12)	0.675
	ETG-CI V4		35.59 (30.41, 40.76)		33.32 (1.09)	0.83 (1.12)	0.055
DMPA-SC V2	DMPA-SC V3	41.85 (33.23, 50.47)	38.27 (32.42, 44.12)	37.13 (1.09)	35.69 (1.09)	0.97 (1.12)	0.707
	DMPA-SC V4		44.56 (34.82, 54.31)		40.03 (1.09)	0.97 (1.12)	0.713
LNG-IUD V2	LNG-IUD V3	40.83 (33.18, 48.49)	32.75 (27.67, 37.84)	37.24 (1.09)	30.23 (1.09)	0.82 (1.12)	0.167
	LNG-IUD V4		32.96 (27.37, 38.56)		30.23 (1.09)	0.82 (1.12)	0.225
Cu-IUD V2	Cu-IUD V3	44.59 (36.03, 53.15)	43.88 (33.54, 54.22)	40.35 (1.09)	38.15 (1.09)	0.98 (1.12)	0.716
	Cu-IUD V4		33.05 (26.22, 39.88)		29.88 (1.09)	0.73 (1.12)	0.048
**EVMS**							
ETG-CI V2	ETG-CI V3	37.33 (29.56, 45.1)	41.18 (31.05, 51.3)	34.85 (1.12)	37.59 (1.12)	1.06 (1.17)	0.602
	ETG-CI V4		31.69 (25.01, 38.36)		29.58 (1.12)	0.80 (1.14)	0.377
DMPA-SC V2	DMPA-SC V3	39.52 (28.26, 50.77)	40.14 (31.31, 48.96)	37.00 (1.13)	38.22 (1.13)	0.99 (1.17)	0.623
	DMPA-SC V4		51.14 (31.8, 70.48)		46.33 (1.14)	1.20 (1.15)	0.157
LNG-IUD V2	LNG-IUD V3	48.55 (32.31, 64.79)	33.01 (24.8, 41.21)	43.08 (1.13)	30.94 (1.13)	0.73 (1.19)	0.212
	LNG-IUD V4		36.13 (27.69, 44.57)		34.24 (1.14)	0.86 (1.15)	0.468
Cu-IUD V2	Cu-IUD V3	46.38 (30.95, 61.8)	49.55 (35.2, 63.9)	42.06 (1.13)	46.14 (1.14)	1.20 (1.18)	0.559
	Cu-IUD V4		33.42 (19.72, 47.12)		29.35 (1.14)	0.67 (1.15)	0.141
**KEMRI**							
ETG-CI V2	ETG-CI V3	50.23 (39.12, 61.33)	55.09 (40.04, 70.15)	45.95 (1.13)	50.10 (1.13)	1.13 (1.18)	0.875
	ETG-CI V4		40.14 (31.79, 48.5)		38.27 (1.14)	0.88 (1.20)	0.100
DMPA-SC V2	DMPA-SC V3	43.85 (29.68, 58.02)	36.90 (28.21, 45.59)	37.24 (1.13)	33.95 (1.13)	0.94 (1.17)	0.509
	DMPA-SC V4		39.86 (28.61, 51.12)		36.06 (1.13)	0.83 (1.19)	0.643
LNG-IUD V2	LNG-IUD V3	34.66 (29.38, 39.94)	32.57 (25.21, 39.92)	33.16 (1.13)	30.31 (1.13)	0.90 (1.17)	0.483

	LNG-IUD V4		30.70 (22.51, 38.89)		27.66 (1.13)	0.80 (1.18)	0.352
Cu-IUD V2	Cu-IUD V3	43.05 (32.31, 53.78)	40.11 (24.65, 55.56)	39.03 (1.13)	33.60 (1.13)	0.86 (1.17)	0.406
	Cu-IUD V4		32.79 (24.43, 41.14)		30.26 (1.13)	0.77 (1.18)	0.242
**Outcome = e-Cadherin score§**							
**EVMS and KEMRI**							
ETG-CI V2	ETG-CI V3	3.786 (3.592, 3.979)	3.926 (3.82, 4.032)	4 (4, 4)	4 (4, 4)	0 (0, 0)	0.045
	ETG-CI V4		3.800 (3.631, 3.969)		4 (4, 4)	0 (0, 0)	0.045
DMPA-SC V2	DMPA-SC V3	3.654 (3.427, 3.881)	3.667 (3.421, 3.912)	4 (3, 4)	4 (3, 4)	0 (0, 0)	0.999
	DMPA-SC V4		3.958 (3.872, 4.045)		4 (4, 4)	0 (0, 0)	0.098
LNG-IUD V2	LNG-IUD V3	3.926 (3.82, 4.032)	3.692 (3.471, 3.914)	4 (4 -4)	4 (3, 4)	0 (0, 0)	0.025
	LNG-IUD V4		3.875 (3.732, 4.018)		4 (4 -4)	0 (0, 0)	0.654
Cu-IUD V2	Cu-IUD V3	3.778 (3.549, 4.006)	3.680 (3.45, 3.91)	4 (4, 4)	4 (3, 4)	0 (0, 0)	0.414
	Cu-IUD V4		3.542 (3.213, 3.871)		4 (3, 4)	0 (-0.5, 0)	0.439
**EVMS**							
ETG-CI V2	ETG-CI V3	3.643 (3.277, 4.009)	3.846 (3.619, 4.073)	4 (3, 4)	4 (4, 4)	0 (0, 0)	0.083
	ETG-CI V4		3.692 (3.402, 3.983)		4 (3, 4)	0 (0, 0)	0.999
DMPA-SC V2	DMPA-SC V3	3.500 (3.072, 3.928)	3.583 (3.159, 4.008)	4 (3, 4)	4 (3, 4)	0 (-0.5, 0.5)	0.898
	DMPA-SC V4		3.900 (3.674, 4.126)		4 (4, 4)	0 (0, 1)	0.292
LNG-IUD V2	LNG-IUD V3	3.833 (3.586, 4.081)	3.364 (2.911, 3.817)	4 (4, 4)	3 (3, 4)	0 (-1, 0)	0.045
	LNG-IUD V4		3.800 (3.498, 4.102)		4 (4, 4)	0 (0, 0)	0.999
Cu-IUD V2	Cu-IUD V3	3.667 (3.172, 4.161)	3.600 (3.231, 3.969)	4 (4, 4)	4 (3, 4)	0 (0, 0)	0.999
	Cu-IUD V4		3.200 (2.543, 3.857)		3.5 (2, 4)	0 (-2, 0)	0.135
**KEMRI**							
ETG-CI V2	ETG-CI V3	3.929 (3.774, 4.083)	4.0 (4.0, 4.0)	4 (4, 4)	4 (4, 4)	0 (0, 0)	0.317
	ETG-CI V4		3.917 (3.733, 4.1)		4 (4, 4)	0 (0, 0)	0.999
DMPA-SC V2	DMPA-SC V3	3.786 (3.54, 4.032)	3.733 (3.405, 4.062)	4 (4, 4)	4 (4, 4)	0 (0, 0)	0.915
	DMPA-SC V4		4.0 (4.0, 4.0)		4 (4, 4)	0 (0, 0)	0.157
LNG-IUD V2	LNG-IUD V3	4.0 (4.0, 4.0)	3.933 (3.79, 4.076)	4 (4, 4)	4 (4, 4)	0 (0, 0)	0.317
	LNG-IUD V4		3.929 (3.774, 4.083)		4 (4, 4)	0 (0, 0)	0.317
Cu-IUD V2	Cu-IUD V3	3.867 (3.672, 4.062)	3.733 (3.405, 4.062)	4 (4 -4)	4 (4, 4)	0 (0, 0)	0.157
	Cu-IUD V4		3.786 (3.451, 4.12)		4 (4, 4)	0 (0, 0)	0.563

LSM: Least Squares Mean. *LSM were calculated using log_{10}mean values for each analyte. §Median and Interquartile Range reported instead of LSM and S.E.

Supplementary Table S8. Immunohistochemical analysis of vaginal lamina propria: comparison between trial arms.

Reference visit	Comparator visit	Mean Reference (95% CI)	Mean Comparator (95% CI)	LSM Reference (S.E.)	LSM Comparator (S.E.)	LSMs Difference (S.E.)	p-value
**Outcome = CD45⁺cells/mm²***							
**EVMS and KEMRI**							
ETG-CI V2	ETG-CI V3	100.2 (82.04, 118.3)	78.13 (69.08,87.17)	91.51 (1.10)	73.47 (1.10)	0.80 (1.13)	0.086
	ETG-CI V4		96.39 (78.02,114.8)		87.31 (1.10)	0.95 (1.12)	0.707
DMPA-SC V2	DMPA-SC V3	80.04 (59.89, 100.2)	86.98 (66.04, 107.9)	65.45 (1.10)	75.97 (1.10)	1.19 (1.13)	0.616
	DMPA-SC V4		112.4 (87.07, 137.7)		99.87 (1.11)	1.37 (1.13)	0.026
LNG-IUD V2	LNG-IUD V3	74.84 (62.4, 87.28)	91.45 (71.04, 111.9)	68.62 (1.10)	84.96 (1.10)	1.18 (1.13)	0.190
	LNG-IUD V4		89.43 (73.12, 105.7)		81.76 (1.11)	1.13 (1.12)	0.191
Cu-IUD V2	Cu-IUD V3	107.7 (80.05, 135.4)	80.51 (61.53, 99.5)	88.27 (1.10)	67.47 (1.10)	0.76 (1.13)	0.092
	Cu-IUD V4		71.38 (60.81, 81.94)		66.86 (1.10)	0.75 (1.12)	0.124
**EVMS**							
ETG-CI V2	ETG-CI V3	87.92 (67.92, 107.9)	77.46 (66.07, 88.86)	81.58 (1.14)	72.77 (1.15)	0.89 (1.20)	0.535
	ETG-CI V4		92.4 (71.02, 113.8)		85.38 (1.14)	1.05 (1.19)	0.789
DMPA-SC V2	DMPA-SC V3	90.87 (60.67, 121.1)	96.09 (49.2, 143)	78.42 (1.16)	78.14 (1.16)	1.02 (1.21)	0.803
	DMPA-SC V4		135.6 (79.12, 192.1)		116.97 (1.17)	1.19 (1.23)	0.246
LNG-IUD V2	LNG-IUD V3	73.25 (56.99, 89.51)	106.4 (61.75, 151.1)	69.42 (1.15)	105.10 (1.17)	1.36 (1.23)	0.088
	LNG-IUD V4		84.56 (66.12, 103)		80.69 (1.18)	1.12 (1.21)	0.362
Cu-IUD V2	Cu-IUD V3	106 (58.85, 153.1)	93.83 (62.39, 125.3)	81.58 (1.15)	81.32 (1.17)	1.04 (1.22)	0.609
	Cu-IUD V4		83.26 (69.21, 97.32)		80.98 (1.16)	1.02 (1.21)	0.669
**KEMRI**							
ETG-CI V2	ETG-CI V3	111.8 (82.4, 141.1)	78.01 (63.4, 92.63)	102.64 (1.14)	74.12 (1.14)	0.71 (1.17)	0.104
	ETG-CI V4		99.22 (67.09, 131.4)		89.62 (1.15)	0.89 (1.17)	0.488
DMPA-SC V2	DMPA-SC V3	75.73 (46.73, 104.7)	79.61 (61.66, 97.55)	56.78 (1.14)	74.41 (1.13)	1.35 (1.17)	0.304
	DMPA-SC V4		96.33 (76.86, 115.8)		89.21 (1.14)	1.25 (1.16)	0.037
LNG-IUD V2	LNG-IUD V3	76.12 (56.01, 96.23)	80.65 (59.21, 102.1)	67.99 (1.13)	73.72 (1.13)	1.09 (1.16)	0.692
	LNG-IUD V4		92.56 (66.58, 118.6)		82.46 (1.14)	1.13 (1.15)	0.402
Cu-IUD V2	Cu-IUD V3	108.7 (75.34, 142.1)	71.49 (47.77, 95.22)	94.02 (1.13)	59.57 (1.13)	0.63 (1.16)	0.036
	Cu-IUD V4		62.7 (48.93,76.48)		57.51 (1.14)	0.59 (1.15)	0.034
**Outcome = CD3⁺cells/mm² ***							

			**EVMS and KEMRI**				
ETG-CI V2	ETG-CI V3	67.77 (54.53, 81.01)	50.41 (42.75, 58.07)	60.48 (1.11)	45.28 (1.12)	0.74 (1.13)	0.085
	ETG-CI V4		67.02 (50.92, 83.11)		58.20 (1.12)	0.97 (1.15)	0.727
DMPA-SC V2	DMPA-SC V3	48.8 (36.11, 61.49)	56.93 (38.14, 75.72)	39.68 (1.12)	46.31 (1.12)	1.22 (1.14)	0.709
	DMPA-SC V4		75.96 (53.53, 98.4)		62.28 (1.12)	1.39 (1.15)	0.042
LNG-IUD V2	LNG-IUD V3	48.02 (38.86, 57.18)	65.19 (46.83, 83.55)	43.03 (1.12)	53.50 (1.12)	1.21 (1.14)	0.259
	LNG-IUD V4		58.43 (44.22, 72.63)		50.86 (1.13)	1.13 (1.15)	0.399
Cu-IUD V2	Cu-IUD V3	76.48 (51.31, 101.6)	52.6 (37.71, 67.5)	58.32 (1.12)	41.76 (1.12)	0.74 (1.14)	0.164
	Cu-IUD V4		44.36 (36.02, 52.7)		39.59 (1.12)	0.67 (1.14)	0.099
			**EVMS**				
ETG-CI V2	ETG-CI V3	64.97 (46.21, 83.74)	50.89 (39.87, 61.92)	58.13 (1.16)	44.96 (1.17)	0.74 (1.22)	0.351
	ETG-CI V4		66.72 (47.11, 86.33)		59.27 (1.16)	1.01 (1.22)	0.839
DMPA-SC V2	DMPA-SC V3	53.1 (34.76, 71.44)	69.86 (26.56, 113.2)	45.87 (1.18)	51.88 (1.18)	1.16 (1.23)	0.813
	DMPA-SC V4		96.72 (47.07, 146.4)		76.23 (1.19)	1.28 (1.27)	0.288
LNG-IUD V2	LNG-IUD V3	51.05 (38.41, 63.69)	83.41 (45.97, 120.9)	47.84 (1.18)	70.48 (1.19)	1.38 (1.26)	0.134
	LNG-IUD V4		55.08 (38.96, 71.19)		50.68 (1.21)	1.06 (1.25)	0.689
Cu-IUD V2	Cu-IUD V3	83.22 (35.5, 130.9)	67.26 (39.84, 94.67)	60.42 (1.18)	56.80 (1.19)	1.03 (1.25)	0.811
	Cu-IUD V4		54.42 (43.39, 65.45)		52.28 (1.18)	0.89 (1.24)	0.534
			**KEMRI**				
ETG-CI V2	ETG-CI V3	70.56 (49.37, 91.76)	49.92 (37.73, 62.11)	62.93 (1.16)	45.58 (1.16)	0.72 (1.18)	0.200
	ETG-CI V4		67.36 (37.24, 97.47)		56.98 (1.18)	0.96 (1.22)	0.654
DMPA-SC V2	DMPA-SC V3	45.41 (25.89, 64.94)	47.44 (33.11, 61.77)	35.41 (1.16)	42.61 (1.16)	1.26 (18)	0.530
	DMPA-SC V4		61.14 (42.05, 80.22)		53.90 (1.16)	1.42 (1.20)	0.014
LNG-IUD V2	LNG-IUD V3	45.59 (31.26, 59.93)	53.05 (33.58, 72.51)	39.53 (1.16)	44.51 (1.16)	1.12 (1.17)	0.636
	LNG-IUD V4		60.58 (37.9, 83.26)		50.97 (1.16)	1.18 (1.19)	0.396
Cu-IUD V2	Cu-IUD V3	70.63 (41.86, 99.41)	40.88 (24.85, 56.91)	56.69 (1.16)	34.02 (1.16)	0.60 (1.17)	0.032
	Cu-IUD V4		36.46 (25, 47.91)		31.82 (1.16)	0.54 (1.19)	0.029
			**Outcome = $CD8^+$cells/mm^2 ***				
			**EVMS and KEMRI**				
ETG-CI V2	ETG-CI V3	47.19 (35.05, 59.33)	35.93 (28.33, 43.53)	39.34 (1.12)	31.03 (1.12)	0.79 (1.14)	0.171
	ETG-CI V4		41.72 (29.94, 53.5)		35.44 (1.13)	0.90 (1.16)	0.514
DMPA-SC V2	DMPA-SC V3	31.19 (22.52, 39.86)	34.59 (21.5, 47.67)	25.07 (1.13)	27.60 (1.13)	1.12 (1.15)	0.815
	DMPA-SC V4		49.86 (32.78, 66.94)		39.37 (1.13)	1.37 (1.17)	0.069

LNG-IUD V2	LNG-IUD V3	32.67 (24.71, 40.64)	43.04 (29.65, 56.44)	28.42 (1.12)	34.23 (1.13)	1.19 (1.15)	0.394
	LNG-IUD V4		37.78 (28.19, 47.37)		32.75 (1.14)	1.09 (1.16)	0.448
Cu-IUD V2	Cu-IUD V3	56.31 (35.3, 77.33)	35.48 (23.59, 47.37)	41.13 (1.12)	26.71 (1.13)	0.96 (1.15)	0.157
	Cu-IUD V4		29.67 (22.64, 36.69)		25.74 (1.13)	0.63 (1.16)	0.090
**EVMS**							
ETG-CI V2	ETG-CI V3	46.64 (25.58, 67.71)	34.92 (24.44, 45.4)	37.10 (1.18)	29.09 (1.19)	0.78 (1.24)	0.407
	ETG-CI V4		39.18 (26.98, 51.37)		34.71 (1.18)	0.87 (1.26)	0.742
DMPA-SC V2	DMPA-SC V3	29.24 (19.12, 39.35)	43.99 (12.4, 75.58)	25.41 (1.21)	32.05 (1.21)	1.26 (1.25)	0.779
	DMPA-SC V4		57.78 (16.69, 98.87)		39.95 (1.22)	1.21 (1.32)	0.502
LNG-IUD V2	LNG-IUD V3	38.46 (22.66, 54.25)	55.2 (29.43, 80.97)	33.21 (1.20)	46.92 (1.22)	1.35 (1.28)	0.191
	LNG-IUD V4		34.87 (24.19, 45.54)		32.01 (1.23)	0.96 (1.30)	0.835
Cu-IUD V2	Cu-IUD V3	62.24 (21.56, 102.9)	44.58 (20.6, 68.57)	43.18 (1.20)	34.19 (1.21)	0.92 (1.27)	0.845
	Cu-IUD V4		34.15 (22.79, 45.52)		30.44 (1.21)	0.77 (1.28)	0.694
**KEMRI**							
ETG-CI V2	ETG-CI V3	47.73 (32.41, 63.05)	36.94 (24.47, 49.42)	41.72 (1.17)	32.95 (1.17)	0.78 (1.20)	0.309
	ETG-CI V4		44.68 (21.01, 68.35)		36.31 (1.19)	0.92 (1.23)	0.514
DMPA-SC V2	DMPA-SC V3	32.72 (18.17, 47.27)	27.69 (20.38, 35.01)	24.80 (1.17)	24.74 (1.17)	1.03 (1.19)	0.826
	DMPA-SC V4		44.2 (30.93, 57.47)		38.96 (1.17)	1.46 (1.21)	0.101
LNG-IUD V2	LNG-IUD V3	28.05 (19.91, 36.18)	34.94 (19.31, 50.57)	25.10 (1.17)	27.74 (1.17)	1.11 (1.19)	0.845
	LNG-IUD V4		39.65 (24.32, 54.98)		33.24 (1.17)	1.21 (1.20)	0.403
Cu-IUD V2	Cu-IUD V3	51.18 (28.22, 74.14)	28.2 (16.72, 39.68)	39.56 (1.17)	22.66 (1.17)	0.57 (1.18)	0.036
	Cu-IUD V4		26.14 (16.41, 35.87)		22.56 (1.17)	0.55 (1.20)	0.040
**Outcome = HLA-DR^{+}cells/mm^{2}***							
**EVMS and KEMRI**							
ETG-CI V2	ETG-CI V3	40.38 (31.17, 49.58)	29.6 (25.56, 33.64)	36.28 (1.09)	27.80 (1.09)	0.78 (1.10)	0.029
	ETG-CI V4		36.45 (29.82, 43.08)		33.28 (1.09)	0.88 (1.11)	0.562
DMPA-SC V2	DMPA-SC V3	38.75 (31.75, 45.75)	33.48 (26.28, 40.69)	34.85 (1.09)	30.18 (1.09)	0.87 (1.11)	0.156
	DMPA-SC V4		40.14 (31.98, 48.29)		36.42 (1.09)	0.99 (1.12)	0.865
LNG-IUD V2	LNG-IUD V3	32.47 (25.88, 39.07)	33.22 (28.14, 38.3)	29.59 (1.09)	30.40 (1.09)	1.02 (1.11)	0.628
	LNG-IUD V4		32.42 (26.94, 37.9)		29.44 (1.09)	1.01 (1.11)	0.782
Cu-IUD V2	Cu-IUD V3	41.18 (31.17, 51.19)	32.65 (24.38, 40.92)	35.81 (1.09)	29.28 (1.09)	0.87 (1.12)	0.266
	Cu-IUD V4		30.12 (24.5, 35.74)		27.28 (1.09)	0.77 (1.11)	0.125

**EVMS**							
ETG-CI V2	ETG-CI V3	45.05 (28.82, 61.28)	31.35 (24.96, 37.74)	40.06 (1.11)	29.13 (1.12)	0.72 (1.14)	0.095
	ETG-CI V4		38.31 (28.62, 48)		35.45 (1.11)	0.85 (1.15)	0.524
DMPA-SC V2	DMPA-SC V3	46.17 (38.23, 54.12)	45.7 (31.31, 60.09)	44.92 (1.13)	41.55 (1.13)	0.92 (1.15)	0.580
	DMPA-SC V4		52.11 (35.78, 68.44)		47.67 (1.14)	0.97 (1.18)	0.842
LNG-IUD V2	LNG-IUD V3	42.13 (28.74, 55.52)	40.64 (33.32, 47.96)	37.84 (1.12)	38.36 (1.14)	0.96 (1.17)	0.817
	LNG-IUD V4		35.07 (26.89, 43.25)		31.51 (1.14)	0.85 (1.17)	0.258
Cu-IUD V2	Cu-IUD V3	44.92 (28.45, 61.4)	40.93 (22.73, 59.12)	42.07 (1.12)	38.42 (1.14)	1.02 (1.16)	0.516
	Cu-IUD V4		34.47 (25.97, 42.97)		32.65 (1.13)	0.81 (1.16)	0.180
**KEMRI**							
ETG-CI V2	ETG-CI V3	35.7 (25.16, 46.24)	27.84 (22.18, 33.51)	32.85 (1.11)	26.62 (1.11)	0.82 (1.16)	0.225
	ETG-CI V4		34.27 (23.83, 44.7)		30.92 (1.12)	0.89 (1.19)	0.727
DMPA-SC V2	DMPA-SC V3	32.91 (22.26, 43.57)	24.53 (21.14, 27.91)	28.56 (1.11)	23.88 (1.11)	0.84 (1.16)	0.303
	DMPA-SC V4		31.59 (25.53, 37.64)		30.05 (1.11)	1.03 (1.17)	0.652
LNG-IUD V2	LNG-IUD V3	24.75 (22.1, 27.39)	27.79 (21.65, 33.92)	24.30 (1.11)	26.04 (1.11)	1.05 (1.15)	0.772
	LNG-IUD V4		30.53 (22.4, 38.66)		28.19 (1.11)	1.13 (1.17)	0.341
Cu-IUD V2	Cu-IUD V3	37.94 (24.12, 51.76)	26.03 (21.67, 30.39)	31.48 (1.11)	24.85 (1.11)	0.79 (1.15)	0.597
	Cu-IUD V4		26.71 (18.74, 34.68)		23.69 (1.11)	0.74 (1.16)	0.407

LSM: Least Squares Mean. *LSM were calculated using $\log_{10}$mean values for each analyte. [§]Median and Interquartile Range reported instead of LSM and S.E.

Supplementary Table S9. HIV-1$_{BaL}$ and HSV-2 strain G infection of cellular models in the presence of CVF: comparison between trial arms.

Reference visit	Comparator visit	Mean Reference (95% CI)	Mean Comparator (95% CI)	LSM Reference (S.E.)	LSM Comparator (S.E.)	LSMs Difference (S.E.)	p-value
**Outcome = HIV-1$_{BaL}$ infection of TZM-bl cells (%)[1]**							
**EVMS and KEMRI**							
ETG-CI V2	ETG-CI V3	117.6 (96.18, 139.1)	134.8 (102.2, 167.4)	117.64 (10.4604)	133.62 (15.7807)	15.9812 (13.8253)	0.2582
	ETG-CI V4		147.6 (107.5, 187.7)		145.42 (18.7400)	27.7759 (17.8859)	0.1325
DMPA-SC V2	DMPA-SC V3	121.2 (92.45, 149.9)	111.2 (78.86, 143.5)	121.18 (13.9204)	111.20 (15.6708)	-9.9799 (13.8795)	0.4791
	DMPA-SC V4		101 (74.74, 127.3)		101.30 (12.5262)	-19.8847 (12.1672)	0.1153
LNG-IUD V2	LNG-IUD V3	132.7 (109.5, 155.8)	139.3 (109.3, 169.3)	132.69 (11.2792)	139.30 (14.6083)	6.6113 (14.0945)	0.6428
	LNG-IUD V4		131.3 (100.2, 162.3)		124.34 (15.0136)	-8.3528 (14.2421)	0.5632
Cu-IUD V2	Cu-IUD V3	130.6 (101, 160.2)	145.2 (111.7, 178.6)	130.59 (14.4445)	147.80 (16.5104)	17.2082 (14.8213)	0.2573
	Cu-IUD V4		124.1 (101.6, 146.5)		123.98 (10.8968)	-6.6183 (17.6417)	0.7104
**EVMS**							
ETG-CI V2	ETG-CI V3	133 (93.22, 172.7)	137.5 (77.12, 198)	132.95 (18.3907)	133.23 (27.2128)	0.2793 (20.4514)	0.9893
	ETG-CI V4		163.1 (91.97, 234.1)		163.05 (32.9000)	30.1000 (30.5778)	0.3429
DMPA-SC V2	DMPA-SC V3	110.7 (61.5, 159.9)	106.1 (37.88, 174.4)	110.68 (22.3444)	106.13 (31.0051)	-4.5500 (18.3455)	0.8087
	DMPA-SC V4		99.88 (46.79, 153)		99.8833 (24.1213)	-10.7917 (16.6261)	0.5296
LNG-IUD V2	LNG-IUD V3	115.6 (77.39 ,153.7)	107.2 (76, 138.3)	115.56 (17.5177)	107.17 (14.3039)	-8.3923 (12.1070)	0.5014
	LNG-IUD V4		88.19 (56.62, 119.8)		79.9071 (13.4040)	-35.6545 (13.7176)	0.0253
Cu-IUD V2	Cu-IUD V3	113.9 (68.98, 158.8)	120.8 (48.63, 192.9)	113.87 (20.6037)	131.83 (36.1593)	17.9635 (34.7314)	0.6185
	Cu-IUD V4		107.6 (67, 148.2)		107.55 (18.4600)	-6.3177 (27.9166)	0.8247
**KEMRI**							
ETG-CI V2	ETG-CI V3	102.3 (82.98, 121.7)	132.3 (94.1, 170.4)	102.33 (8.9581)	132.25 (17.6606)	29.9180 (18.8240)	0.1360
	ETG-CI V4		129.5 (90.19, 168.8)		127.48 (16.6398)	25.1484 (18.6615)	0.2015
DMPA-SC V2	DMPA-SC V3	130.9 (92.74, 169)	115.9 (91.3, 140.5)	130.89 (17.5072)	115.89 (11.2815)	-14.9921 (21.2531)	0.4940
	DMPA-SC V4		102.1 (81.04, 123.2)		102.17 (9.5749)	-28.7189 (17.6219)	0.1285
LNG-IUD V2	LNG-IUD V3	147.5 (117.5, 177.6)	167.2 (119.3, 215)	147.53 (14.0049)	167.15 (22.2925)	19.6145 (24.1172)	0.4297
	LNG-IUD V4		162 (118.2, 205.8)		159.42 (20.3709)	11.8846 (20.6008)	0.5739
Cu-IUD V2	Cu-IUD V3	145.1 (102, 188.2)	161.5 (126.4, 196.5)	145.09 (20.0853)	161.47 (16.3511)	16.3755 (14.4383)	0.2758
	Cu-IUD V4		137 (109.8, 164.2)		136.96 (12.6591)	-8.1289 (23.7213)	0.7369

**Outcome = HSV-2 strain G infection of HEC-1A (%)[1]**							
**EVMS and KEMRI**							
ETG-CI V2	ETG-CI V3	33.69 (19.88, 47.51)	52.02 (12.28, 91.76)	33.5616 (6.6287)	34.8218 (7.1823)	1.2602 (6.1058)	0.8382
	ETG-CI V4		77.37 (21.03, 133.7)		42.9896 (8.7992)	9.8583 (6.7366)	0.1569
DMPA-SC V2	DMPA-SC V3	44.94 (21.76, 68.12)	45.86 (25.17, 66.56)	37.5081 (8.7754)	38.3736 (6.9485)	0.8655 (11.4442)	0.9404
	DMPA-SC V4		26.97 (15.5, 38.45)		26.9612 (5.5565)	-10.5451 (10.2352)	0.3131
LNG-IUD V2	LNG-IUD V3	31.5 (20.39, 42.62)	34.03 (16.54, 51.52)	31.5036 (5.4160)	33.8352 (8.4689)	2.3316 (8.0235)	0.7737
	LNG-IUD V4		29.23 (14.69, 43.77)		28.2935 (6.9140)	-3.2101 (7.5605)	0.6749
Cu-IUD V2	Cu-IUD V3	33.78 (19.6, 47.97)	23.37 (12.02, 34.72)	33.7823 (6.9126)	23.7529 (5.4677)	-10.0294 (7.4135)	0.1873
	Cu-IUD V4		36.43 (17.71, 55.15)		37.0346 (8.9242)	3.2523 (8.4779)	0.7045
**EVMS**							
ETG-CI V2	ETG-CI V3	28.85 (10.79, 46.92)	46.09 (19.8, 72.39)	28.8521 (8.3614)	44.2247 (11.9348)	15.3726 (9.7751)	0.1417
	ETG-CI V4		82.59 (-2.021, 167.2)		46.3204 (10.5357)	17.4683 (8.6880)	0.0672
DMPA-SC V2	DMPA-SC V3	38.42 (8.87, 67.96)	37.87 (19.75, 55.98)	38.4165 (13.4243)	37.8662 (8.2319)	-0.5504 (14.3365)	0.9701
	DMPA-SC V4		32.59 (11.18, 53.99)		32.5861 9.7239)	-5.8304 (16.0574)	0.7234
LNG-IUD V2	LNG-IUD V3	36.9 (15.3, 58.5)	50.39 (12.96, 87.82)	36.8984 (9.9122)	49.6335 (16.8891)	12.7351 (16.0129)	0.4431
	LNG-IUD V4		19.14 (4.932, 33.35)		16.1399 (5.5600)	-20.7585 (8.1148)	0.0247
Cu-IUD V2	Cu-IUD V3	37.23 (14.26, 60.2)	36.54 (10.44, 62.65)	37.2258 (10.5424)	37.7284 (11.2422)	0.5026 (12.2220)	0.9679
	Cu-IUD V4		48.3 (15.31, 81.28)		51.0935 (14.2053)	13.8677 (11.9205)	0.2712
**KEMRI**							
ETG-CI V2	ETG-CI V3	38.91 (15.3, 62.52)	57.52 (-20.73, 135.8)	39.3759 (10.1600)	24.6177 (6.6213)	-14.7582 (4.8919)	0.0109
	ETG-CI V4		71.28 (-15.78, 158.3)		38.6010 (14.8095)	0.7297 (10.6073)	0.9465
DMPA-SC V2	DMPA-SC V3	50.96 (11.61, 90.32)	53.25 (14.28, 92.22)	36.5997 (11.9026)	38.8811 (11.5799)	2.2814 (18.4753)	0.9040
	DMPA-SC V4		21.36 (9.626, 33.09)		21.3672 (5.3645)	-15.2122 (13.2210)	0.2728
LNG-IUD V2	LNG-IUD V3	26.83 (15.23, 38.43)	20.94 (8.661, 33.22)	26.8281 (5.4084)	20.9382 (5.7241)	-5.8899 (6.7906)	0.4004
	LNG-IUD V4		36.22 (12.62 ,59.81)		36.8720 (10.6679)	10.0439 (9.8127)	0.3261
Cu-IUD V2	Cu-IUD V3	30.8 (10.67, 50.93)	14.59 (6.12, 23.06)	30.7981 (9.3856)	14.5903 (3.9494)	-16.2078 (9.5516)	0.1118
	Cu-IUD V4		27.1 (3.084, 51.13)		26.6050 (11.0817)	-4.1930 (11.9094)	0.7302

LSM: Least Squares Mean. [1]Infection levels (%) were normalized vs internal assay control of infectivity in the absence of CVF.

Supplementary Table S10. p24 outcomes in ecto-cervical tissue and PBMCs from EVMS site: comparison between trial arms.

Reference visit	Comparator visit	Mean Reference (95% CI)	Mean Comparator (95% CI)	LSM Reference (S.E.)	LSM Comparator (S.E.)	LSMs Difference (S.E.)	p-value
**Outcome = [$p24_{d21}$] (pg/mL) in ecto-cervical biopsies***							
ETG-CI V2	ETG-CI V3	866.3 (-958.3, 2691)	263.9 (-208.7, 736.5)	1.2168 (0.2495)	1.1936 (0.2654)	-1.1936 (0.2654)	0.9503
	ETG-CI V4		580.6 (-331.9, 1493)		1.5487 (0.2794)	0.3320 (0.2878)	0.2694
DMPA-SC V2	DMPA-SC V3	831 (-854.2, 2516)	18.06 (-5.423, 41.54)	1.2150 (0.3026)	0.9381 (0.2287)	-0.2769 (0.3234)	0.3983
	DMPA-SC V4		96.87 (-4.526, 198.3)		1.3368 (0.2382)	0.1218 (0.3750)	0.7512
LNG-IUD V2	LNG-IUD V3	13948 (168.9, 27727)	6204 (-2957, 15365)	2.5172 (0.4774)	1.8461 (0.4407)	-0.6711 (0.5988)	0.2710
	LNG-IUD V4		352.3 (-331.1, 1036)		1.4415 (0.2927)	-1.0757 (0.6958)	0.1483
Cu-IUD V2	Cu-IUD V3	7121 (-521.4, 14763)	2268 (-948, 5485)	2.1567 (0.4559)	1.6610 (0.4697)	-0.4957 (0.6248)	0.4336
	Cu-IUD V4		1437 (-558.2, 3432)		1.6380 (0.3877)	-0.5187 (0.4652)	0.2867
**Outcome = $p24_{AUCd3\text{-}d21}$ (pg*d/mL) in ecto-cervical biopsies***							
ETG-CI V2	ETG-CI V3	4540 (-4706 ,13787)	1165 (-380.8, 2710)	2.4978 (0.1888)	2.5994 (0.1829)	0.1015 (0.2541)	0.6916
	ETG-CI V4		1533 (-56.26, 3123)		2.6403 (0.1860)	0.1424 (0.1972)	0.4830
DMPA-SC V2	DMPA-SC V3	5805 (-6191, 17800)	245.9 (152, 339.8)	2.5497 (0.2297)	2.3205 (0.1513)	-0.2292 (0.2140)	0.2922
	DMPA-SC V4		367.7 (163, 572.3)		2.4485 (0.1001)	-0.1012 (0.2691)	0.7139
LNG-IUD V2	LNG-IUD V3	82634 (-17866, 183133)	19666 (-12344, 51675)	3.5393 (0.3872)	2.9761 (0.3337)	-0.5632 (0.4534)	0.2235
	LNG-IUD V4		980.2 (-610, 2570)		2.4904 (0.1867)	-1.0489 (0.4741)	0.0471
Cu-IUD V2	Cu-IUD V3	29347 (-4980, 63674)	15159 (-11194, 41511)	3.2615 (0.3373)	2.8290 (0.3577)	-0.4324 (0.4758)	0.3705
	Cu-IUD V4		7667 (-5975, 21310)		2.6774 (0.2917)	-0.5841 (0.3239)	0.0968
**Outcome = [$p24_{d14}$] (pg/mL) in PBMCs***							
ETG-CI V2	ETG-CI V4	35401 (9402, 61400)	11931 (4013, 19848)	3.8240 (0.3725)	3.1018 (0.4227)	-0.7223 (0.3489)	0.0589
DMPA-SC V2	DMPA-SC V4	34339 (13474, 55204)	8909 (5108, 12709)	3.5963 (0.5002)	3.4023 (0.3706)	-0.1940 (0.5101)	0.7110
LNG-IUD V2	LNG-IUD V4	50158 (6378, 93939)	8767 (934.4, 16600)	3.8585 (0.4137)	2.8299 (0.5285)	-1.0286 (0.6677)	0.1505
Cu-IUD V2	Cu-IUD V4	39203 (15550, 62857)	14107 (3265, 24949)	3.9917 (0.3653)	2.9237 (0.5923)	-1.0680 (0.6748)	0.1430
**Outcome = $p24_{AUCd3\text{-}d21}$ (pg*d/mL) in PBMCs***							

ETG-CI V2	ETG-CI V4	121109 (47243, 194974)	38594 (13673, 63514)	4.5008 (0.3016)	3.8655 (0.3154)	-0.6354 (0.2532)	0.0261
DMPA-SC V2	DMPA-SC V4	110808 (45650, 175965)	45577 (19608, 71546)	4.3420 (0.3860)	4.1141 (0.3261)	-0.2279 (0.4030)	0.5830
LNG-IUD V2	LNG-IUD V4	168354 (13094, 323614)	29608 (3011, 56206)	4.4936 (0.3204)	3.6856 (0.3892)	-0.8079 (0.4937)	0.1287
Cu-IUD V2	Cu-IUD V4	124371 (40381, 208361)	55463 (13571, 97355)	4.6723 (0.2420)	3.8295 (0.4697)	-0.8429 (0.4882)	0.1161

LSM: Least Squares Mean. *LSM were calculated using log_{10}mean values for each analyte.

Supplementary Table S11. Correlation between endpoints at each visit for all trial arms combined.

		V2		V3		V4	
**Reference endpoint**	**Comparator endpoint**	***r***	**p-value**	***r***	**p-value**	***r***	**p-value**
pH	Nugent score	0.4173	<0.0001	0.2458	0.0115	0.3902	0.0001
	L-lactic acid	-0.2934	0.002	-0.2078	0.0334	-0.3616	0.0002
	D-lactic acid	-0.3471	0.0002	-0.2428	0.0126	-0.2959	0.0028
	Glycogen	-0.2147	0.0256	-0.2678	0.006	-0.2778	0.0056
	RANTES	0.3481	0.0002	0.247	0.0111	0.1549	0.1239
	SLPI	-0.1264	0.1902	-0.0961	0.3293	0.1608	0.11
	HBD2	-0.1676	0.0815	-0.0673	0.4953	-0.0276	0.7852
	Epithetial thickness	-0.1335	0.1706	-0.208	0.0359	-0.0324	0.7527
	Epithelial layers	-0.0894	0.3599	-0.1563	0.1167	0.0272	0.7912
	Epithelial CD45	0.1134	0.245	0.054	0.5898	0.0145	0.8881
	Epithelial CD3	0.052	0.5951	0.0388	0.6983	-0.006	0.9531
	Epithelial CD1a	-0.022	0.8243	-0.0071	0.9439	-0.0441	0.6699
	Epithelial CD8	0.0667	0.4948	0.0215	0.83	-0.0117	0.9096
	Epithelial HLA-DR	-0.0223	0.82	0.01	0.9204	-0.1624	0.112
	E-Cadherin	0.1008	0.304	0.076	0.4457	-0.0844	0.4134
	Lamina propria CD45	0.141	0.1494	-0.2857	0.0038	-0.1397	0.1722
	Lamina propria CD3	0.0859	0.3815	-0.2876	0.0035	-0.0924	0.368
	Lamina propria CD8	0.0318	0.7461	-0.1568	0.1173	0.0043	0.9666
	Lamina propria HLA-DR	-0.0975	0.3198	-0.196	0.0495	-0.036	0.7261
	Serum E2	-0.22	0.0215	-0.1296	0.1877	0.1323	0.1894
	Serum P4	-0.2466	0.0097	0.0046	0.9631	-0.0783	0.4436
	HIV-1 infection	0.4259	<0.0001	0.196	0.0451	0.2387	0.0173
	HSV-2 infection	0.0867	0.3725	0.0459	0.6434	0.0821	0.4239
Nugent score	L-lactic acid	-0.2874	0.0024	-0.4054	<0.0001	-0.5023	<0.0001
	D-lactic acid	-0.2436	0.0107	-0.32	0.0009	-0.2898	0.0035
	Glycogen	-0.2381	0.0131	-0.2985	0.0021	-0.2847	0.0045
	RANTES	0.0901	0.3514	0.0286	0.7722	-0.0367	0.7173
	SLPI	0.0628	0.5165	-0.1716	0.08	-0.1813	0.071

	HBD2	-0.2454	0.0101	-0.0276	0.7798	0.0135	0.8939
	Epithetial thickness	0.1265	0.1941	0.2201	0.0262	0.1918	0.0599
	Epithelial layers	0.0959	0.3259	0.1675	0.0924	0.2307	0.023
	Epithelial CD45	0.0941	0.3348	-0.063	0.5292	-0.0444	0.6661
	Epithelial CD3	0.0717	0.4627	-0.0487	0.6268	-0.0315	0.7593
	Epithelial CD1a	-0.1886	0.0552	-0.261	0.0084	-0.2265	0.0265
	Epithelial CD8	0.0818	0.4023	-0.0289	0.7732	-0.0272	0.7913
	Epithelial HLA-DR	-0.0832	0.3943	-0.1837	0.0646	-0.1094	0.286
	E-Cadherin	-0.0424	0.6661	-0.0705	0.4791	-0.1226	0.234
	Lamina propria CD45	-0.0222	0.8209	-0.021	0.835	0.0167	0.8707
	Lamina propria CD3	0.0066	0.9468	-0.006	0.9521	0.0328	0.75
	Lamina propria CD8	-0.0073	0.9405	0.0281	0.7806	0.0247	0.8105
	Lamina propria HLA-DR	-0.0794	0.4187	0.0784	0.4359	0.1616	0.1139
	Serum E2	-0.0113	0.9073	0.0979	0.3202	-0.0256	0.8003
	Serum P4	-0.1047	0.2787	-0.0138	0.8888	-0.038	0.7101
	HIV-1 infection	0.4132	<0.0001	0.3717	0.0001	0.4575	<0.0001
	HSV-2 infection	0.0749	0.4411	0.1449	0.1423	0.2104	0.0386
L-lactic acid	D-lactic acid	0.2475	0.0095	0.2353	0.0157	0.4686	<0.0001
	Glycogen	0.4707	<0.0001	0.3474	0.0003	0.4678	<0.0001
	RANTES	-0.0638	0.5096	0.0836	0.3967	-0.0602	0.5517
	SLPI	0.2686	0.0047	0.3974	<0.0001	0.3768	0.0001
	HBD2	0.1925	0.0449	0.1879	0.055	0.3259	0.0009
	Epithetial thickness	0.0233	0.8118	0.0575	0.5659	-0.0221	0.8299
	Epithelial layers	-0.0001	0.9993	-0.0066	0.9476	-0.0067	0.9479
	Epithelial CD45	0.1041	0.2858	-0.1515	0.1286	0.0161	0.8759
	Epithelial CD3	0.1454	0.1351	-0.1727	0.0827	0.0065	0.9496
	Epithelial CD1a	0.0998	0.3137	-0.0573	0.5692	0.242	0.0175
	Epithelial CD8	0.1667	0.0862	-0.1589	0.1106	0.0047	0.9635
	Epithelial HLA-DR	0.0388	0.6917	-0.0599	0.55	0.0914	0.3735
	E-Cadherin	0.1051	0.2837	0.1403	0.1574	0.1055	0.3062
	Lamina propria CD45	0.0156	0.8738	-0.0573	0.5691	-0.0416	0.686
	Lamina propria CD3	0.0165	0.8668	-0.0738	0.4634	-0.08	0.4362
	Lamina propria CD8	0.0179	0.8552	-0.0864	0.3903	-0.0669	0.5148
	Lamina propria HLA-DR	0.0274	0.7802	-0.0967	0.3362	-0.1248	0.2233

	Serum E2	0.611	<0.0001	0.0805	0.414	-0.0827	0.4134
	Serum P4	0.2604	0.0063	-0.0179	0.8562	0.0493	0.6299
	HIV-1 infection	-0.2697	0.0046	-0.2177	0.0257	-0.3098	0.0018
	HSV-2 infection	-0.2143	0.0259	-0.06	0.5454	-0.1002	0.3288
D-lactic acid	Glycogen	0.2508	0.0089	0.4781	<0.0001	0.3769	0.0001
	RANTES	-0.0622	0.5207	-0.0798	0.4187	-0.0857	0.3963
	SLPI	0.182	0.0582	0.5218	<0.0001	0.3163	0.0013
	HBD2	0.3653	0.0001	-0.009	0.9272	0.2462	0.0135
	Epithetial thickness	-0.0273	0.7805	0.0781	0.4352	0.0914	0.3732
	Epithelial layers	0.0466	0.6337	0.1396	0.1618	0.0178	0.8628
	Epithelial CD45	-0.0502	0.6078	0.2938	0.0027	-0.0347	0.7357
	Epithelial CD3	-0.039	0.6902	0.3114	0.0014	-0.0337	0.7429
	Epithelial CD1a	0.0872	0.3788	0.0957	0.3413	-0.0288	0.7804
	Epithelial CD8	-0.0345	0.7242	0.3436	0.0004	-0.0673	0.5122
	Epithelial HLA-DR	-0.0016	0.9869	0.1783	0.073	-0.08	0.436
	E-Cadherin	-0.0848	0.3877	0.0142	0.887	0.0117	0.9097
	Lamina propria CD45	-0.028	0.7761	0.078	0.438	-0.0619	0.5469
	Lamina propria CD3	0.003	0.976	0.0506	0.6154	-0.0809	0.4311
	Lamina propria CD8	0.014	0.8867	0.0633	0.5293	-0.1331	0.1936
	Lamina propria HLA-DR	0.0449	0.648	0.1217	0.2254	-0.1057	0.3028
	Serum E2	0.0519	0.592	0.0673	0.495	-0.1025	0.3102
	Serum P4	0.3132	0.0009	-0.0431	0.6625	0.0416	0.6842
	HIV-1 infection	-0.2282	0.017	-0.2099	0.0316	-0.2963	0.0029
	HSV-2 infection	-0.1321	0.173	0.1164	0.2395	-0.1121	0.2742
Glycogen	RANTES	-0.0857	0.3776	-0.0857	0.3871	-0.0691	0.4991
	SLPI	0.1448	0.1349	0.1754	0.075	0.1239	0.2241
	HBD2	0.0341	0.7259	-0.057	0.5653	0.1155	0.2573
	Epithetial thickness	-0.0022	0.9823	0.044	0.6619	0.0209	0.8406
	Epithelial layers	-0.1025	0.2957	-0.0067	0.947	0.0059	0.9551
	Epithelial CD45	0.1224	0.2111	0.1206	0.2297	0.002	0.9849
	Epithelial CD3	0.0996	0.3098	0.1378	0.1695	0.0036	0.9721
	Epithelial CD1a	-0.0516	0.605	-0.0512	0.6129	0.1278	0.2196

	Epithelial CD8	0.1157	0.2376	0.2051	0.0397	0.0274	0.7921
	Epithelial HLA-DR	0.0376	0.7022	-0.0293	0.7711	0.1894	0.066
	E-Cadherin	0.1103	0.2605	0.1249	0.211	0.1379	0.185
	Lamina propria CD45	0.0629	0.5237	-0.1011	0.317	-0.0865	0.4045
	Lamina propria CD3	0.077	0.4348	-0.1115	0.2695	-0.092	0.3754
	Lamina propria CD8	0.0887	0.3682	-0.1013	0.316	-0.1147	0.2682
	Lamina propria HLA-DR	0.1089	0.2688	-0.0746	0.4605	0.0089	0.9316
	Serum E2	-0.0149	0.8784	-0.0546	0.5818	-0.0977	0.3388
	Serum P4	0.1975	0.0405	-0.0686	0.4888	-0.0757	0.4635
	HIV-1 infection	-0.1939	0.0443	-0.2484	0.011	-0.2544	0.0119
	HSV-2 infection	-0.1899	0.0501	-0.0717	0.4715	-0.0333	0.7486
RANTES	SLPI	-0.0546	0.5727	0.1495	0.128	-0.0387	0.7024
	HBD2	-0.1107	0.2517	-0.0763	0.4392	-0.0793	0.4326
	Epithelial thickness	-0.0848	0.3853	-0.1829	0.0658	-0.0951	0.3543
	Epithelial layers	0.0479	0.6239	-0.1448	0.1466	-0.1036	0.3124
	Epithelial CD45	-0.008	0.935	0.2848	0.0037	0.0435	0.6725
	Epithelial CD3	-0.037	0.7051	0.1943	0.0504	0.0365	0.7229
	Epithelial CD1a	0.0787	0.4269	0.2415	0.015	0.0633	0.5399
	Epithelial CD8	0.0074	0.9401	0.2062	0.0376	0.0757	0.4609
	Epithelial HLA-DR	-0.0197	0.8407	0.3392	0.0005	0.0576	0.5752
	E-Cadherin	-0.1619	0.0972	0.0559	0.5748	0.0616	0.5509
	Lamina propria CD45	-0.0532	0.5881	0.0976	0.3314	0.0268	0.7941
	Lamina propria CD3	-0.0669	0.4959	0.0529	0.5996	0.0037	0.9712
	Lamina propria CD8	-0.0758	0.4397	0.0368	0.7145	0.0332	0.7472
	Lamina propria HLA-DR	-0.0942	0.3368	-0.0692	0.4918	0.0239	0.8165
	Serum E2	-0.1452	0.1318	-0.1344	0.1716	-0.0558	0.5811
	Serum P4	-0.0886	0.3593	-0.0668	0.4982	-0.0383	0.7084
	HIV-1 infection	0.0745	0.4415	0.1968	0.0442	-0.0864	0.3949
	HSV-2 infection	-0.1472	0.1283	-0.0392	0.6931	-0.0668	0.5153
SLPI	HBD2	0.0737	0.4464	0.213	0.0292	0.3458	0.0004
	Epithelial thickness	0.0199	0.8387	0.0969	0.3327	-0.0677	0.51
	Epithelial layers	0.112	0.2506	0.0865	0.3871	-0.0546	0.5955
	Epithelial CD45	-0.0964	0.3232	0.2038	0.04	0.0315	0.7597
	Epithelial CD3	-0.0697	0.4759	0.2256	0.0226	-0.0174	0.8655

	Epithelial CD1a	-0.026	0.7934	0.0225	0.8231	0.1295	0.2087
	Epithelial CD8	-0.0527	0.59	0.2333	0.0183	-0.0092	0.9286
	Epithelial HLA-DR	-0.0222	0.8208	0.1028	0.3039	-0.0941	0.3591
	E-Cadherin	0.1121	0.2527	0.0854	0.3911	0.1095	0.288
	Lamia propria CD45	-0.0734	0.4545	0.116	0.248	-0.08	0.4361
	Lamia propria CD3	-0.1243	0.2041	0.0819	0.4155	-0.1334	0.1928
	Lamia propria CD8	-0.0924	0.3459	0.0774	0.4416	-0.106	0.3013
	Lamia propria HLA-DR	-0.0342	0.7282	0.219	0.0278	-0.2259	0.0261
	Serum E2	0.3893	<0.0001	0.2692	0.0055	0.2066	0.0392
	Serum P4	0.095	0.3256	0.0137	0.8897	0.0054	0.9583
	HIV-1 infection	-0.0526	0.5872	-0.1005	0.3076	-0.1243	0.2202
	HSV-2 infection	-0.0677	0.4865	-0.0016	0.9868	0.1385	0.176
HBD2	Epithelial thickness	0.0649	0.5067	0.0489	0.6258	0.0597	0.5612
	Epithelial layers	0.0075	0.9385	0.0233	0.8161	0.1182	0.2488
	Epithelial CD45	-0.1074	0.271	0.0657	0.5118	0.2266	0.0256
	Epithelial CD3	-0.0821	0.4003	0.0847	0.3974	0.2024	0.0467
	Epithelial CD1a	0.031	0.7547	0.0435	0.666	0.2014	0.0491
	Epithelial CD8	-0.1024	0.2937	0.0771	0.441	0.1705	0.0949
	Epithelial HLA-DR	-0.107	0.2726	-0.0038	0.9697	0.0344	0.7379
	E-Cadherin	-0.0806	0.4113	0.0337	0.7356	0.1388	0.1776
	Lamina propria CD45	-0.0572	0.5602	0.0908	0.3667	0.228	0.0247
	Lamina propria CD3	-0.0577	0.5566	0.1034	0.3035	0.152	0.1371
	Lamina propria CD8	-0.0473	0.6302	0.0512	0.6114	0.1073	0.2956
	Lamina propria HLA-DR	0.0112	0.909	-0.004	0.9683	0.1005	0.3273
	Serum E2	0.1471	0.127	0.0442	0.6542	-0.0987	0.3287
	Serum P4	0.2616	0.006	-0.0251	0.7998	-0.0391	0.7026
	HIV-1 infection	-0.0246	0.7995	-0.0012	0.9903	0.0723	0.4768
	HSV-2 infection	0.0228	0.815	-0.0356	0.7196	0.0445	0.665
Epithelial thickness	Epithelial layers	0.7395	<0.0001	0.8269	<0.0001	0.8804	<0.0001
	Epithelial CD45	-0.3627	0.0001	-0.4765	<0.0001	-0.487	<0.0001
	Epithelial CD3	-0.2932	0.0022	-0.4416	<0.0001	-0.3858	0.0001
	Epithelial CD1a	-0.4656	<0.0001	-0.4023	<0.0001	-0.4115	<0.0001

	Epithelial CD8	-0.2123	0.0281	-0.3835	0.0001	-0.2905	0.0039
	Epithelial HLA-DR	-0.3763	0.0001	-0.4525	<0.0001	-0.3543	0.0004
	E-Cadherin	-0.2702	0.0051	-0.1494	0.1339	0.0092	0.9292
	Lamina propria CD45	0.0309	0.7535	0.1655	0.0999	-0.0164	0.8739
	Lamina propria CD3	0.0507	0.6055	0.1898	0.0585	0.0083	0.9363
	Lamina propria CD8	0.1051	0.2835	0.2254	0.0241	0.0076	0.9418
	Lamina propria HLA-DR	0.4045	<0.0001	0.3401	0.0005	0.1946	0.0575
	Serum E2	0.1555	0.1097	0.4244	<0.0001	0.1519	0.1375
	Serum P4	0.1386	0.1544	-0.008	0.9366	0.0058	0.9555
	HIV-1 infection	0.0482	0.6221	0.1019	0.3082	0.1228	0.2331
	HSV-2 infection	0.1944	0.0458	0.0683	0.4954	0.0568	0.5867
Epithelial layers	Epithelial CD45	-0.3518	0.0002	-0.3715	0.0001	-0.4453	<0.0001
	Epithelial CD3	-0.3019	0.0016	-0.337	0.0005	-0.3705	0.0002
	Epithelial CD1a	-0.349	0.0003	-0.3167	0.0013	-0.3295	0.001
	Epithelial CD8	-0.2169	0.0248	-0.2811	0.0042	-0.2858	0.0045
	Epithelial HLA-DR	-0.3116	0.0011	-0.326	0.0008	-0.3251	0.0012
	E-Cadherin	-0.2146	0.0271	-0.1748	0.0789	-0.0184	0.8589
	Lamina propria CD45	-0.0638	0.5161	0.086	0.3951	0.0025	0.9806
	Lamina propria CD3	-0.0578	0.5563	0.1083	0.2834	0.0232	0.8223
	Lamina propria CD8	-0.0039	0.9684	0.1727	0.0858	0.0621	0.5475
	Lamina propria HLA-DR	0.2889	0.0027	0.2854	0.004	0.1763	0.0858
	Serum E2	0.1155	0.2363	0.3916	<0.0001	0.1556	0.128
	Serum P4	0.01	0.9182	0.0643	0.5211	-0.0244	0.8146
	HIV-1 infection	-0.0333	0.7331	0.1238	0.2151	0.1756	0.087
	HSV-2 infection	0.1666	0.0879	0.1376	0.1677	0.0426	0.6837
Epithelial CD45	Epithelial CD3	0.9671	<0.0001	0.9673	<0.0001	0.974	<0.0001
	Epithelial CD1a	0.3612	0.0002	0.3609	0.0002	0.4191	<0.0001
	Epithelial CD8	0.9252	<0.0001	0.9503	<0.0001	0.9184	<0.0001
	Epithelial HLA-DR	0.5826	<0.0001	0.6892	<0.0001	0.6117	<0.0001
	E-Cadherin	0.167	0.0871	0.2324	0.0188	0.1232	0.2319
	Lamina propria CD45	0.5657	<0.0001	0.1854	0.0648	0.4347	<0.0001
	Lamina propria CD3	0.5875	<0.0001	0.1407	0.1625	0.4322	<0.0001
	Lamina propria CD8	0.5263	<0.0001	0.1621	0.1071	0.374	0.0002
	Lamina propria HLA-DR	0.1278	0.1916	-0.0394	0.6973	0.3032	0.0027

	Serum E2	0.0483	0.6214	-0.2807	0.0043	-0.2285	0.0244
	Serum P4	0.0262	0.7892	-0.0654	0.514	-0.0461	0.6575
	HIV-1 infection	0.1081	0.2679	0.0377	0.7071	-0.029	0.7793
	HSV-2 infection	-0.0182	0.8532	-0.0001	0.9988	0.0941	0.3669
Epithelial CD3	Epithelial CD1a	0.3787	0.0001	0.336	0.0006	0.4017	<0.0001
	Epithelial CD8	0.9707	<0.0001	0.9718	<0.0001	0.9503	<0.0001
	Epithelial HLA-DR	0.5844	<0.0001	0.6467	<0.0001	0.6145	<0.0001
	E-Cadherin	0.1516	0.1209	0.2269	0.0219	0.1218	0.2371
	Lamina propria CD45	0.5674	<0.0001	0.1723	0.0864	0.4733	<0.0001
	Lamina propria CD3	0.6209	<0.0001	0.1419	0.159	0.4861	<0.0001
	Lamina propria CD8	0.5815	<0.0001	0.1617	0.108	0.4236	<0.0001
	Lamina propria HLA-DR	0.187	0.0549	-0.0139	0.8907	0.3622	0.0003
	Serum E2	0.1011	0.3002	-0.2628	0.0076	-0.2364	0.0198
	Serum P4	0.0465	0.6343	-0.0635	0.526	-0.0376	0.7172
	HIV-1 infection	0.1246	0.2009	-0.002	0.9838	-0.0543	0.5991
	HSV-2 infection	0.0181	0.8543	0.0181	0.8566	0.1363	0.1902
Epithelial CD1a	Epithelial CD8	0.3495	0.0003	0.3423	0.0005	0.3776	0.0001
	Epithelial HLA-DR	0.5781	<0.0001	0.526	<0.0001	0.5363	<0.0001
	E-Cadherin	0.1987	0.0442	0.2093	0.0357	0.1117	0.281
	Lamina propria CD45	0.3041	0.0018	0.0049	0.9614	0.1858	0.0714
	Lamina propria CD3	0.3135	0.0013	0.0451	0.6577	0.1473	0.1543
	Lamina propria CD8	0.2444	0.0129	0.044	0.6652	0.1605	0.1203
	Lamina propria HLA-DR	0.0678	0.4962	0.0622	0.5406	0.1394	0.1779
	Serum E2	0.017	0.8643	-0.23	0.0207	-0.0856	0.4068
	Serum P4	-0.0091	0.927	-0.0618	0.5392	0.0021	0.9836
	HIV-1 infection	-0.0967	0.3286	-0.0402	0.69	-0.2066	0.0446
	HSV-2 infection	-0.0342	0.7317	-0.0594	0.5549	-0.1545	0.1392
Epithelial CD8	Epithelial HLA-DR	0.5507	<0.0001	0.6598	<0.0001	0.5985	<0.0001
	E-Cadherin	0.1432	0.1431	0.239	0.0155	0.1813	0.077
	Lamina propria CD45	0.5725	<0.0001	0.1823	0.0694	0.4417	<0.0001
	Lamina propria CD3	0.6396	<0.0001	0.1505	0.135	0.4457	<0.0001
	Lamina propria CD8	0.6217	<0.0001	0.2101	0.0359	0.4167	<0.0001

	Lamina propria HLA-DR	0.2428	0.0122	0.0643	0.525	0.3928	0.0001
	Serum E2	0.1399	0.1506	-0.2372	0.0164	-0.2127	0.0365
	Serum P4	0.0557	0.5686	-0.0472	0.6374	-0.1207	0.244
	HIV-1 infection	0.1522	0.1175	0.0177	0.8596	-0.018	0.8617
	HSV-2 infection	0.0155	0.875	-0.0179	0.8583	0.1057	0.3106
Epithelial HLA-DR	E-Cadherin	0.1872	0.0547	0.2304	0.0198	0.2485	0.0146
	Lamina propria CD45	0.4928	<0.0001	0.1932	0.0541	0.3147	0.0018
	Lamina propria CD3	0.5235	<0.0001	0.1735	0.0843	0.3008	0.0029
	Lamina propria CD8	0.4785	<0.0001	0.1681	0.0946	0.266	0.0088
	Lamina propria HLA-DR	0.2983	0.0019	0.1728	0.0857	0.4698	<0.0001
	Serum E2	-0.0287	0.7689	-0.2525	0.0104	-0.0781	0.4468
	Serum P4	-0.0588	0.5476	-0.0908	0.364	-0.0959	0.3551
	HIV-1 infection	-0.0094	0.9231	-0.0326	0.7451	-0.0927	0.369
	HSV-2 infection	-0.0416	0.6718	-0.0736	0.462	-0.0845	0.4179
E-Cadherin	Lamina propria CD45	0.0842	0.3932	-0.0235	0.8153	0.1144	0.2694
	Lamina propria CD3	0.0918	0.3518	-0.0816	0.4174	0.0801	0.4405
	Lamina propria CD8	0.1111	0.2592	-0.0487	0.6286	0.109	0.2931
	Lamina propria HLA-DR	-0.003	0.9754	-0.0523	0.6038	-0.0094	0.9278
	Serum E2	0.0395	0.688	-0.2802	0.0042	0.0862	0.4039
	Serum P4	-0.1342	0.1701	-0.1856	0.0605	-0.3864	0.0001
	HIV-1 infection	0.0258	0.7929	0.013	0.8965	-0.0157	0.8792
	HSV-2 infection	0.0167	0.8654	0.0065	0.9482	-0.0414	0.6917
Lamina propria CD45	Lamina propria CD3	0.9472	<0.0001	0.9535	<0.0001	0.9566	<0.0001
	Lamina propria CD8	0.869	<0.0001	0.798	<0.0001	0.8741	<0.0001
	Lamina propria HLA-DR	0.6443	<0.0001	0.4866	<0.0001	0.4855	<0.0001
	Serum E2	0.1189	0.2246	0.0277	0.7833	-0.1272	0.2142
	Serum P4	0.057	0.5617	-0.0574	0.5686	0.0015	0.9885
	HIV-1 infection	0.0974	0.3206	-0.1787	0.0738	-0.1104	0.2842
	HSV-2 infection	0.0704	0.4752	0.0289	0.7742	0.2148	0.0376
Lamina propria CD3	Lamina propria CD8	0.9495	<0.0001	0.8598	<0.0001	0.8955	<0.0001
	Lamina propria HLA-DR	0.681	<0.0001	0.5102	<0.0001	0.521	<0.0001
	Serum E2	0.0953	0.3312	0.0627	0.5334	-0.1158	0.2586
	Serum P4	0.0831	0.3969	-0.0477	0.636	0.0396	0.7035

	HIV-1 infection	0.1183	0.2273	-0.1732	0.0832	-0.0744	0.4712
	HSV-2 infection	0.077	0.435	0.0195	0.8462	0.2741	0.0075
Lamina propria CD8	Lamina propria HLA-DR	0.7383	<0.0001	0.4862	<0.0001	0.4499	<0.0001
	Serum E2	0.0785	0.4235	0.1087	0.2793	-0.0009	0.9928
	Serum P4	0.0519	0.5974	-0.0165	0.8696	-0.0148	0.8867
	HIV-1 infection	0.135	0.1677	-0.1622	0.1052	-0.0464	0.6532
	HSV-2 infection	0.1142	0.2462	0.0337	0.7379	0.2281	0.027
Lamina propria HLA-DR	Serum E2	0.0799	0.4158	0.2188	0.0279	-0.0592	0.5648
	Serum P4	0.0427	0.6638	0.032	0.7507	0.0789	0.4472
	HIV-1 infection	-0.0589	0.549	-0.2227	0.0252	0.1013	0.326
	HSV-2 infection	0.019	0.8478	-0.0619	0.5385	0.1254	0.2285
Serum E2	Serum P4	0.1823	0.0578	0.2185	0.0251	0.1178	0.2479
	HIV-1 infection	-0.0562	0.5619	0.0898	0.3622	0.0344	0.7351
	HSV-2 infection	-0.1129	0.2447	0.0375	0.7054	0.1145	0.2642
Serum P4	HIV-1 infection	-0.0955	0.3235	-0.1178	0.2313	-0.1207	0.2366
	HSV-2 infection	0.0001	0.9994	-0.0245	0.8048	0.0094	0.9276
HIV-1 infection	HSV-2 infection	0.1327	0.1711	-0.0557	0.5745	0.0905	0.3781
